# Tradeoff between robustness and elaboration in carotenoid networks produces cycles of avian color diversification

**DOI:** 10.1186/s13062-015-0073-6

**Published:** 2015-08-20

**Authors:** Alexander V. Badyaev, Erin S. Morrison, Virginia Belloni, Michael J. Sanderson

**Affiliations:** Department of Ecology and Evolutionary Biology, University of Arizona, Tucson, AZ 85721 USA

**Keywords:** Metabolic networks, Robustness, Metabolic distance, Diversification

## Abstract

**Background:**

Resolution of the link between micro- and macroevolution calls for comparing both processes on the same deterministic landscape, such as genomic, metabolic or fitness networks. We apply this perspective to the evolution of carotenoid pigmentation that produces spectacular diversity in avian colors and show that basic structural properties of the underlying carotenoid metabolic network are reflected in global patterns of elaboration and diversification in color displays. Birds color themselves by consuming and metabolizing several dietary carotenoids from the environment. Such fundamental dependency on the most upstream external compounds should intrinsically constrain sustained evolutionary elongation of multi-step metabolic pathways needed for color elaboration unless the metabolic network gains robustness – the ability to synthesize the same carotenoid from an additional dietary starting point.

**Results:**

We found that gains and losses of metabolic robustness were associated with evolutionary cycles of elaboration and stasis in expressed carotenoids in birds. Lack of metabolic robustness constrained lineage’s metabolic explorations to the immediate biochemical vicinity of their ecologically distinct dietary carotenoids, whereas gains of robustness repeatedly resulted in sustained elongation of metabolic pathways on evolutionary time scales and corresponding color elaboration.

**Conclusions:**

The structural link between length and robustness in metabolic pathways may explain periodic convergence of phylogenetically distant and ecologically distinct species in expressed carotenoid pigmentation; account for stasis in carotenoid colors in some ecological lineages; and show how the connectivity of the underlying metabolic network provides a mechanistic link between microevolutionary elaboration and macroevolutionary diversification.

**Reviewers:**

This article was reviewed by Junhyong Kim, Eugene Koonin, and Fyodor Kondrashov. For complete reports, see the Reviewers’ reports section.

**Electronic supplementary material:**

The online version of this article (doi:10.1186/s13062-015-0073-6) contains supplementary material, which is available to authorized users.

## Background

What determines evolutionary pathways of phenotypic diversification? Historically, the emphasis was placed on whether structural consideration of trait development or environment of trait functioning are more influential at predicting and directing evolutionary change [1]. One insightful attempt to integrate these perspectives showed that the patterns of connectivity among elements of the deterministic landscape (e.g., gene networks) that underlie trait variation allows reconciliation of the “structuralist” and “functionalist” perspectives [2]: the patterns of connectivity that are formed by historical association of such elements delineate the pathways available for future evolution [3–6]. In light of recent empirical findings that the age of many genes (and most other components of current adaptations) vastly exceeds the duration of their current genomic and physiological associations (i.e., current functions), such a framework also reconciles historical contingency and contemporary evolutionary dynamics of adaptations [4, 7, 8]. However empirical tests of this framework are rare and require demonstration that historical associations among elements of deterministic network are not themselves modified by recent evolution. A particularly clear example would come from deterministic networks whose origin clearly predates their subsequent use.

A combination of known biochemical reactions among naturally occurring carotenoids (here denoted “global network” of carotenoid biosynthesis) is a connected network (Additional file 1: Appendix S1 and the references therein). The majority of enzymatic reactions of this network appears to have evolved in the context of bacterial evolution [9–16], such that most carotenoid-producing enzymatic networks in other taxa studied to date (e.g., fungi, plants, animals) are subsets of this global network (Additional file 1: Appendix S1). Importantly, carotenoids are commonly used for coloration by taxa that cannot themselves produce carotenoids from non-carotenoids (e.g., most animals) [17]. We therefore hypothesized that the evolutionary diversification in carotenoid-based coloration in these taxa should be a reflection of the structure and patterns of enzymatic connectivity of the underlying “global network” of carotenoid biosynthesis.

**Fig. 1:**
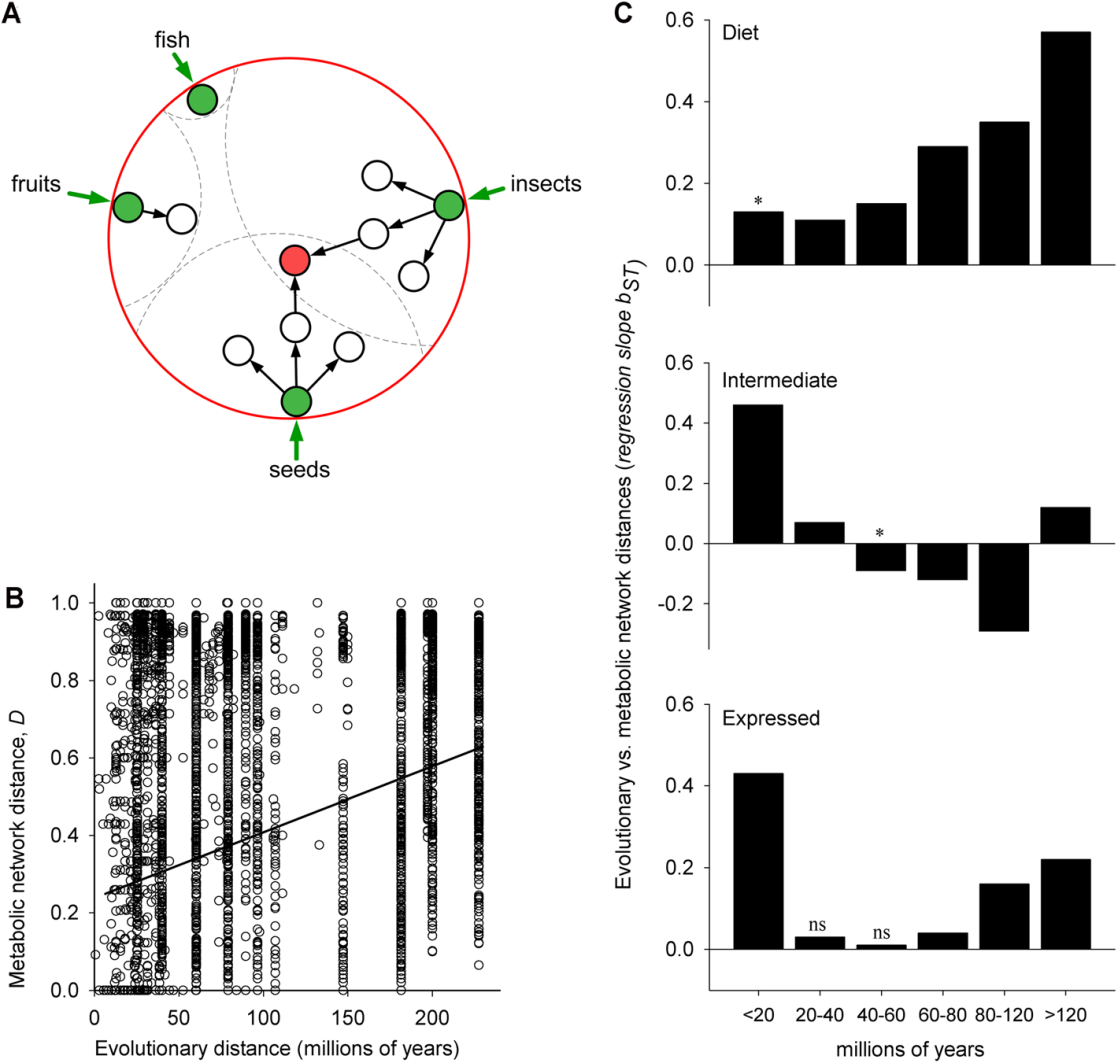
Metabolic pathway elongation and robustness are structurally linked in avian carotenoid-producing networks. **a** Metabolic elongation (*reactions shown as arrows*) necessarily starts with dietary carotenoids (*green circles*) and over evolutionary time can invade the biochemical domain of another dietary carotenoid (i.e., nodes reachable from another dietary compound; dashed line), resulting in dietary robustness of a shared compound (*red circle*). **b** Metabolic distance between species increases with their evolutionary distance (patristic distance). **c** Relationship between metabolic and evolutionary divergence (slopes from Fig. 1b: standardized regression coefficient, b_ST_, in standard deviations (SD)) in carotenoid-producing pathways over different time scales calculated separately for subnetworks containing only dietary, intermediate, or plumage-expressed compounds. Across time periods, divergence in dietary carotenoids increases with evolutionary distance (*upper*), whereas divergence in intermediate (metabolized, but not expressed) compounds (*middle*) lessened with evolutionary distance as a result of greater occupancy of shared metabolic space among distinct dietary (external) starting points (Fig. 1a). Concordance between metabolic and evolutionary distances was particularly strong in most recently diverged species (<20 my) and was largely produced by differential expression of compounds (*lower panel*) and their metabolic evolution (*middle panel*) and not by switching between dietary starting points in metabolic pathways (*upper panel*). *– indicates slopes different from zero at *P* = 0.05, *ns* marks two slopes that did not differ from zero, all other slopes differ from zero at *P* < 0.01

For example, despite the extraordinary diversity of carotenoid colors they express, birds occupy a small and insular group of nodes in the global carotenoid biochemical network, expressing fewer than 5 % of all known carotenoids in their plumage (e.g., [9]). Like most other animals, birds cannot produce carotenoids from non-carotenoids [18, 19], and, therefore, the evolution of their carotenoid-based coloration should be delineated by the structure of enzymatic pathways in the vicinity of dietary “entry-points” – ecologically-specific carotenoid compounds that birds ingest as metabolic precursors of plumage carotenoids (Fig. 1a). The interplay between fundamental dependency of avian carotenoid metabolism on external precursors *and* the biochemical connectivity among these precursors exposes mechanistic links between micro- and macroevolutionary aspects of avian color evolution (Fig. 1a). For example, differences in connectivity of the biochemical network in the vicinity of external dietary points (starting points of carotenoid metabolism in all birds) may influence the potential for carotenoid-based color diversification among ecologically distinct avian lineages. Similarly, elongation of metabolic pathways, commonly associated with color elaboration, depends on fluctuations in availability of the external compounds over evolutionary time [20, 21], implying that lineages utilizing more than one external dietary carotenoid for their internal carotenoid metabolism may have greater potential to evolve elaborate carotenoid displays than lineages depending on a single dietary precursor. Further, the connected nature of carotenoid biochemical network, where most compounds can be reached from any point (Additional file 2: Figure S2), implies that metabolic elongation starting from one dietary entry has the potential, over evolutionary time, to invade the biochemical vicinity of another ecologically distinct dietary entry (Fig. 1a). Such potential connectivity gives us an opportunity to empirically evaluate the relative contribution of ecological (e.g., switching among external dietary compounds) and biochemical (e.g., internal metabolic diversification) aspects to evolutionary diversification in avian colors. Finally, the study of expressed enzymatic pathways in birds in relation to topology and connectivity of the global carotenoid networks (of which avian networks are a small part) enables us to examine structural characteristics of elements or reactions that are most likely to be retained or lost in avian evolution.

Carotenoids play a multitude of functions in birds, and carotenoid-based pigmentation of plumage has evolved multiple times (e.g., [22, 23]). Here we specifically focused on species that express carotenoids in their plumage because these species are most likely to be under selection for metabolic elongation or metabolic efficiency of production of their expressed carotenoids – the amount of carotenoids needed for coloration is typically far larger than required for other carotenoid functions, such as trans-membrane transport or antioxidization. First, we build metabolic networks for plumage carotenoids for 159 species that have been studied to May 2014 (Additional file 1: Appendix S1 and Additional file 3: Appendix S2). Distribution of these species on avian phylogeny gives us insight into 113.5 million years of avian evolution (Additional file 4: Figure S1, Methods). We then compared the metabolic pathway-based phylogeny and the molecular-based supertree for these species and compared evolutionary divergence for upstream and downstream elements of the carotenoid-producing networks. In birds, closely related species often diverge in carotenoid networks, whereas evolutionary distant and ecologically distinct taxa can be highly convergent in their expressed carotenoids [24, 25]. Here we hypothesize that these patterns occur because diversification of avian carotenoid-producing pathways occurs on a limited biochemical space; such that over evolutionary time many species of birds repeatedly occupied shared metabolic space of the global carotenoid network.

Second, we examine whether diversification in carotenoid metabolism in general, and periodic species convergence in particular, are quantitatively predictable from the structure of biochemical network on which birds diversify. We present evidence for strong structural trade-off between robustness – ability to sustain gain or loss of compounds without extinction – and elongation of carotenoid metabolic pathways in species that depend on external (dietary) starting points for their carotenoid production. We test empirical predictions of this trade-off in extant bird species, explore its likely evolutionary trajectories, and use it to calibrate potential for metabolic diversification across avian taxa that utilize diverse dietary carotenoids.

## Results and discussion

### Metabolic and phylogenetic distances

**Fig. 2:**
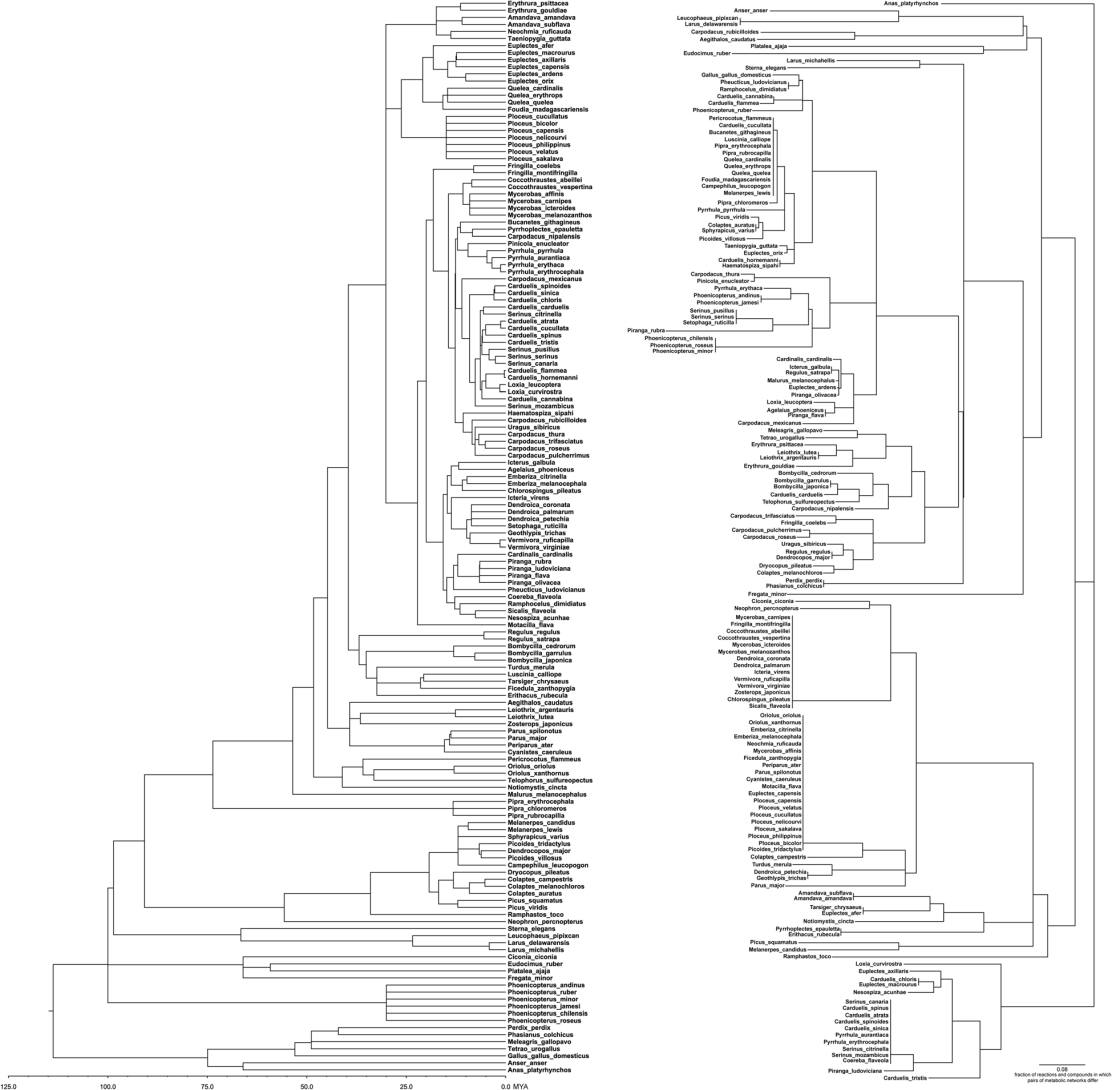
Distinct topologies of molecular-based supertree (*left*) and the metabolic pathway-based supertree (*right*) for species under this study. Scale bar on metabolic pathway phylogeny shows fraction of reactions and compounds in which pair of carotenoid-producing metabolic networks differ

Metabolic divergence (the fraction of reactions and compounds in which networks differ; Methods) increased with the phylogenetic distance between species (Fig. 1b, Spearman’s *r* = 0.18, *P* < 10^−4^), although the relationship was weakened by frequent convergence of distantly related taxa in plumage carotenoids (Fig. 2, see below). The topologies of the metabolic pathway-based phylogeny and molecular supertree strongly differed (Fig. 2, Additional file 5: Figure S3).

We thus calculated the extent to which evolutionary divergence correlated with metabolic divergence separately for elements and reactions comprised only of “dietary”, “intermediate” (metabolized, but not expressed), and “expressed” carotenoids and partitioned these correlations by periods of 20 million years (Fig. 1c). We found that diversification in expressed carotenoids was the greatest in recently diverged species (<20 my in lower panel of Fig. 1c) and was produced by metabolic diversification (middle panel, Fig. 1b) and not by shifts in dietary (external) starting points (upper panel, Fig. 1c). Surprisingly, over longer evolutionary time, as species’ diets continued to diverge, intermediate and plumage compounds and reactions became increasingly similar (Fig. 1c), suggesting increasing occupancy of shared metabolic space by longer metabolic pathways originating from different dietary starting points (Fig. 1a). Such recurrent occupancy of shared metabolic space reveals a structural basis for previously unexplained, but often documented evolutionary convergence in plumage carotenoids among distant species and also puzzlingly rapid divergence of closely related species in expressed carotenoids (e.g., [22, 25, 26]) (Fig. 2).

For example, Red-headed quelea (*Quelea erythrops*) and Cream-backed woodpecker (*Campephilus leucopogon*) have identical carotenoids in their plumage (lutein, zeaxanthin,α-doradexanthin, astaxanthin, adonirubin, canthaxanthin) despite more than 90 million years since divergence (Additional file 4: Figure S1, Additional file 3: Appendix S2). In contrast, rapid metabolic diversification had led to highly distinct plumage carotenoids between recently diverged and ecologically similar taxa, such as bullfinches *Pyrrhula pyrrhula* and *P. aurantiaca* that have diverged less than 5 million years ago or cotingas *Rupicola rupicola* and *R. peruviana* that have diverged less than 4 million years ago, with neither sister groups sharing the expressed carotenoids (Additional file 3: Appendix S2 and references therein).

### Structure of global carotenoid network predicts avian color diversification

**Fig. 3:**
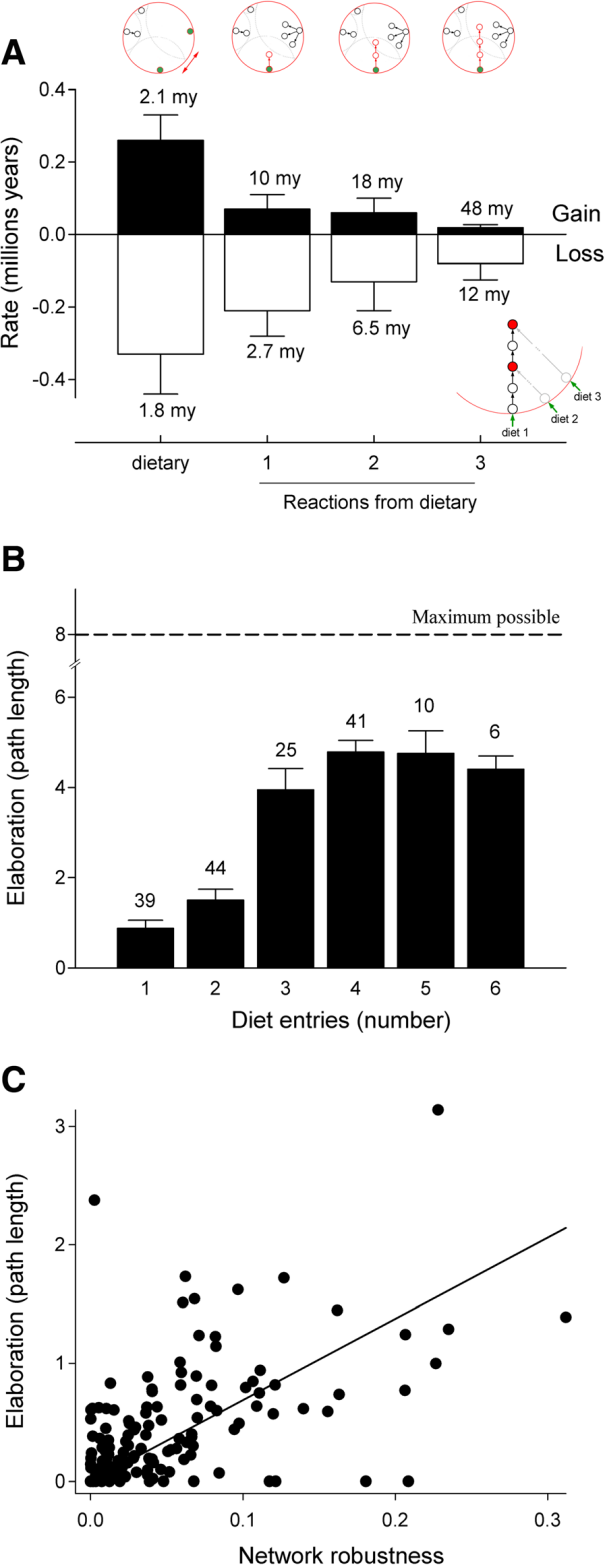
Structure of carotenoid metabolic network establishes rules of avian carotenoid diversification. **a** Gains and losses (in millions of years ± 1SE) of plumage carotenoids during avian evolution in relation to their distance (in reactions) from dietary starting points. Numbers above bars are average frequency in millions of years. Insert (*lower right*): Hypothesis that observed metabolic elongation is sustained by recurrent acquisition of a new pathway linked to an additional dietary (external) compound. **b** Metabolic elongation (longest path from dietary to plumage carotenoids, in reactions) is only achieved by species utilizing >2 different dietary carotenoids (*F*_1,165_ = 35.9, *P* < 10^−4^). Numbers above bars are numbers of species in each category. Species with only direct (non-metabolized) deposition of dietary carotenoids into plumage have path length = 0. “Maximum possible elaboration” (*dashed line*) is the longest metabolic pathway structurally possible in connected avian carotenoid network (Fig. 6a) that can be accomplished from most dietary starting points. **c** Network robustness (an average ratio of compounds that retain their expression in the plumage when any one enzymatic reaction in the network is deleted – a measure of redundancy) enables metabolic elongation. This metric excludes dietary compounds (Additional file 8: Table S1). Independent phylogenetic linear contrasts are shown

Capitalizing on evolutionary lability of carotenoid compounds and enzymatic reactions across phylogeny of our study species (Additional file 4: Figure S1 and Additional file 6: Figure S4, Methods) we categorized gains and losses of compounds (Additional file 7: Table S2) in relation to their topological position within the biochemical network of carotenoids (Additional file 5: Figure S3). These data showed that frequency of losses exceeded that of gains for all but dietary compounds and that structure of the network makes sustained metabolic elongation from a single dietary starting point unlikely (Fig. 3a, Methods). For example, the combined time required for an evolving lineage to gain a compound three metabolic reactions downstream from a single dietary entry was 48 million years (Fig. 3a), whereas time required for evolution of four reaction-long linear metabolic pathway from a single dietary entrance is comparable to the age of birds. These structural data are in clear contrast with empirical observations – elongations of three or four reactions away from dietary starting compound are common in birds and can evolve fast (Additional file 3: Appendix S2). An alternative route to metabolic elongation, suggested by these data is, instead, to acquire a new pathway from a different dietary compound every 2-3 metabolic steps (i.e., <10–15 my, Fig. 3a insert); that is to acquire redundancy of pathways by which a compound expressed in the plumage can be reached from dietary carotenoids (Fig. 1a).

These structural inferences are strongly corroborated by empirical data: only the species with three or more distinct dietary starting points accomplished significant elongation of their carotenoid-producing pathways (Fig. 3b), whereas metabolic pathways of 83 extant species that depended on less than two dietary carotenoids (Additional file 3: Appendix S2) was strictly confined to a single metabolic step around dietary entries. Even when we controlled statistically for variation in dietary (most upstream) starting points ([27], Additional file 8: Table S1]), network robustness was still associated with pathway elongation across all species (Fig. 3c, “maximum” network: *b*_*ST*_ = 0.73, *t* = 23.53, *P* < 10^−3^; “minimum” network: *b*_*ST*_ = 0.48, *t* = 17.53, *P* < 10^−3^), likely by lessening the probability of compound’s loss during evolution of long metabolic pathways (Fig. 3a). Comparison of these empirical data with the “maximum possible elaboration” – the longest metabolic pathway structurally possible in the connected avian carotenoid network (Fig. 3b, Additional file 5: Figure S3) – suggests that dietary robustness enables metabolic pathway elongation by extending time available for its evolution.

### Compound’s network topology and representation in avian evolution

**Fig. 4:**
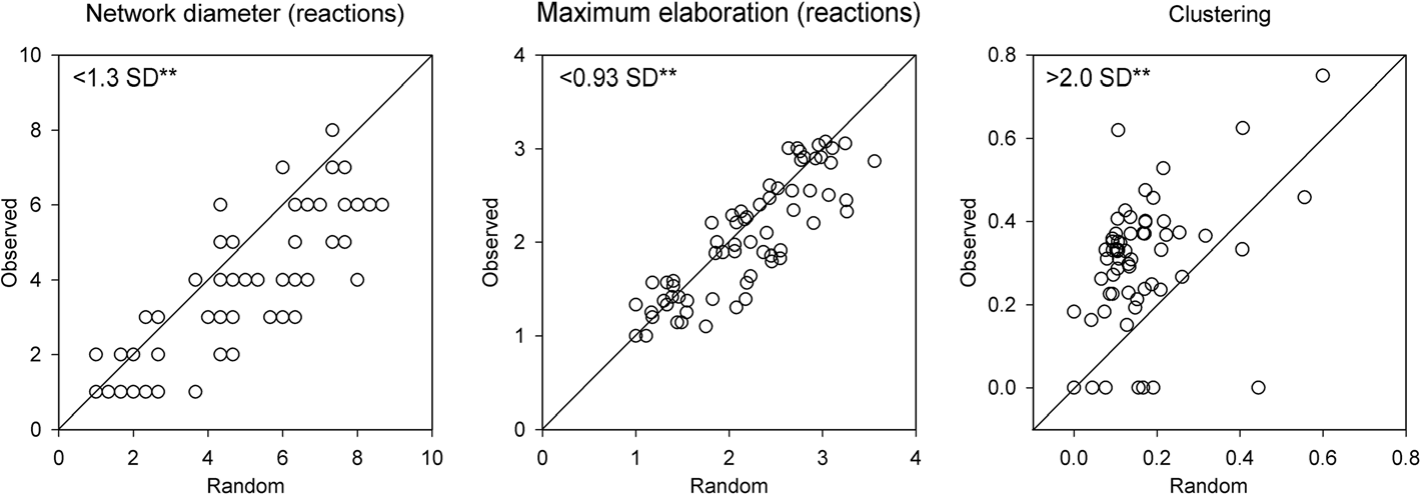
Birds have shorter and more interconnected enzymatic pathways from dietary to plumage carotenoids than is expected by degree-preserving Erdos-Reyni randomization. Points on diagonal would indicate no difference between random and observed network characteristics. Data points are species network characteristics for either observed (*y-axis*) or randomized (under degree-preserving rewiring, *x-axis*) networks. SD is standard deviation for comparison of observed and randomized species networks. **indicates *P* < 0.01

For each species, we compared the structure of observed carotenoid network with that expected under degree-preserving random rewiring of the same number of carotenoid compounds and reactions (Fig. 4; Erdos-Reyni randomization; [28]). We found that the length of observed metabolic pathways from dietary to expressed carotenoids was 0.93 standard deviations (SD) shorter, network modularity 2.4 SD greater, and average pathway length (network diameter) 1.3 SD smaller than that expected under a null model (Fig. 4). Evolving highly redundant and modular carotenoid networks is ubiquitous across birds [29], despite great diversity in elements of these networks (Additional file 6: Figure S4 and Additional file 3: Appendix S2), metabolizing from 1 to 20 carotenoids and synthesizing compounds as far as eight enzymatic steps from a dietary starting point. Despite such diversity, the shared feature of these networks was their high “vulnerability” [30] – the propensity for disruption by deletion of any one of the existing compounds. This measure excluded variation in dietary compounds (Additional file 8: Table S1), because all avian species lose plumage carotenoids in absence of dietary carotenoids, but instead reflects a particular patterns of wiring of avian metabolic networks – their unusual modularity and shortness of pathways for the number of compounds that they contain (Fig. 4).

**Fig. 5:**
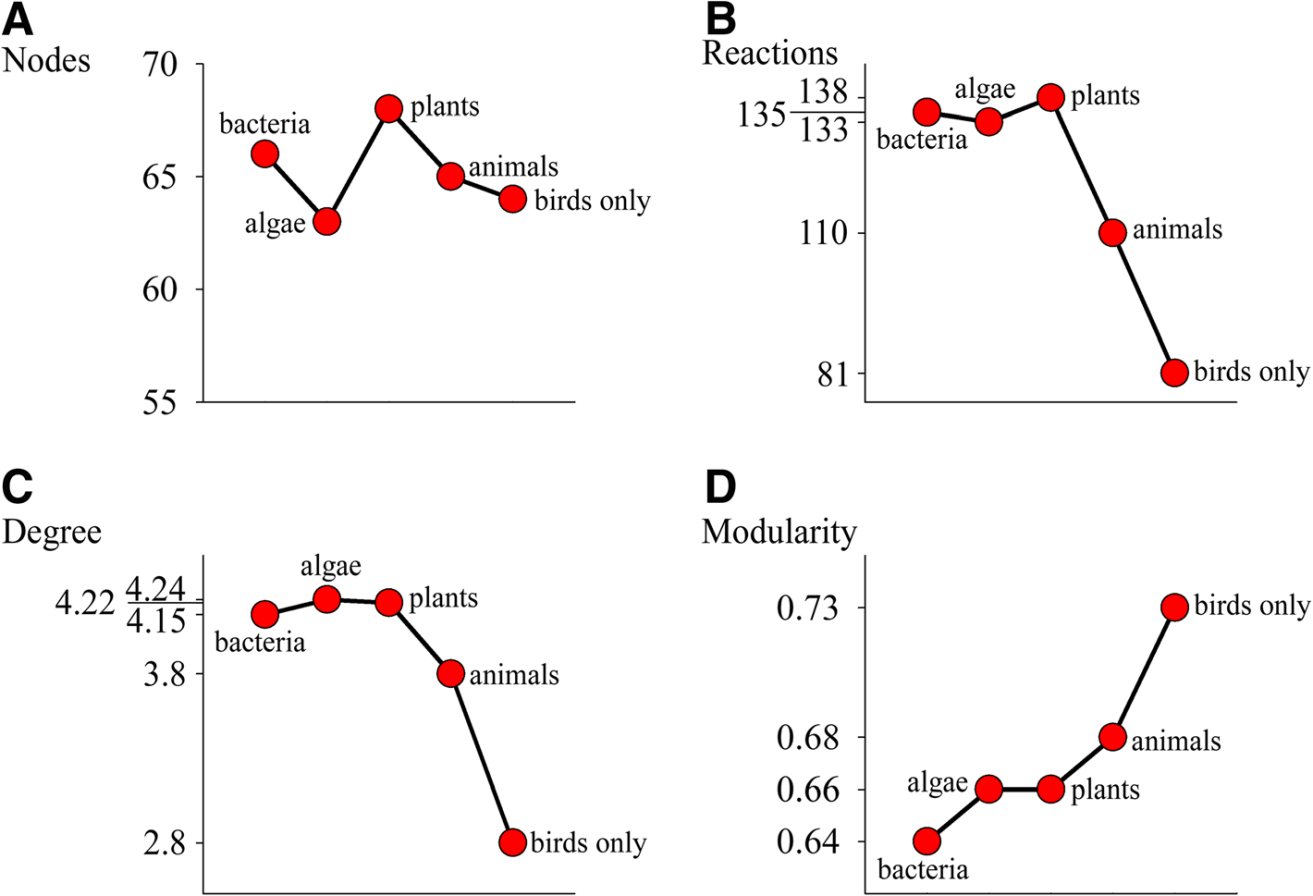
Characteristics of the same subset of global carotenoid network (Additional file 4: Figure S1) as expressed in combined “bacterial”, “algae”, “plants”, “animals”, and “birds only” networks. For the similar number of nodes in the subset of global network (**a**), birds have less connected (**b**, **c**) and more modular (**d**) network (data in Additional file 1: Appendix S1)

Recurrent evolution of linearity and modularity in avian carotenoid pathways might explain strong prevalence of losses over gains in carotenoid networks during avian evolution (Fig. 3a) – birds seem to gradually lose access to modules (metabolic “islands”, Additional file 5: Figure S3) that evolve within lineages (see also [29]). This could account for a general “pruning” of the avian subset of global carotenoid network over evolutionary time; comparison of the same area of the global carotenoid network as it is expressed in other taxa revealed that, for the same number of nodes, combined avian network has lesser connectivity and greater modularity than does combined bacterial, algae, or plant networks (Fig. 5, based on Additional file 1: Appendix S1).

**Fig. 6:**
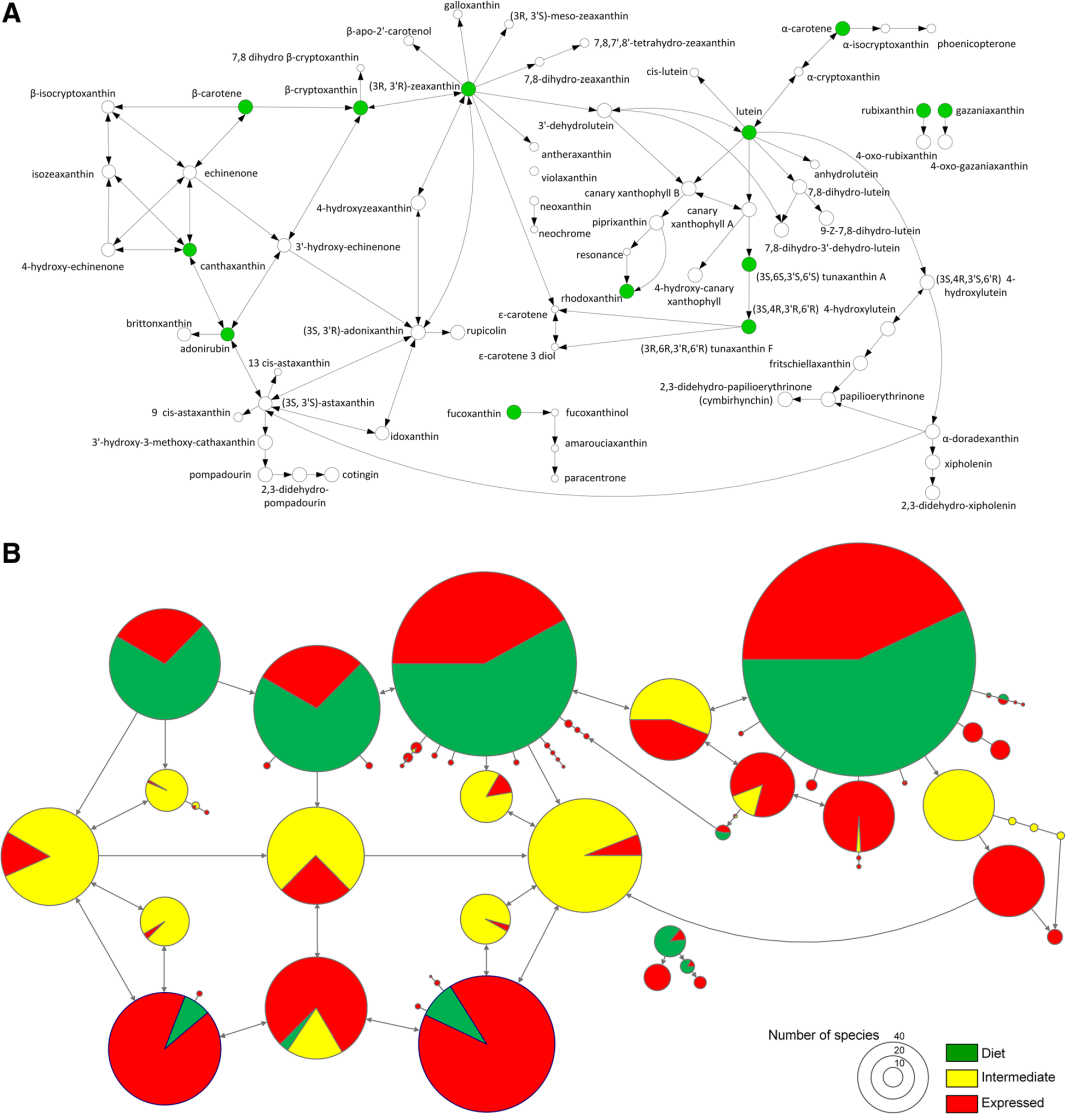
**a** Schematics of avian carotenoid network (66 compounds, 97 enzymatic reactions) in species under this study (Additional file 3: Appendix S2). Green circles show dietary carotenoids. **b** Evolutionary consequences of network vulnerability. Avian carotenoid network in which diameter of each node represents the number of species with that carotenoid compound. Compounds and reactions found in <5 species are not shown. Colors show the proportion of species in which a compound is dietary, intermediate or expressed in plumage. For dietary nodes (*those with some proportion of green*), frequency of plumage expression (*red*) shows the number of species that deposit this dietary compound unchanged, without metabolic conversion. Similar frequencies of dietary, intermediate and plumage compounds across species is most consistent with the “boom-bust” cycle of network use, where substantial occupation of network by species with elongated metabolic pathways is alternating with species that have only starting (dietary) nodes

**Fig. 7:**
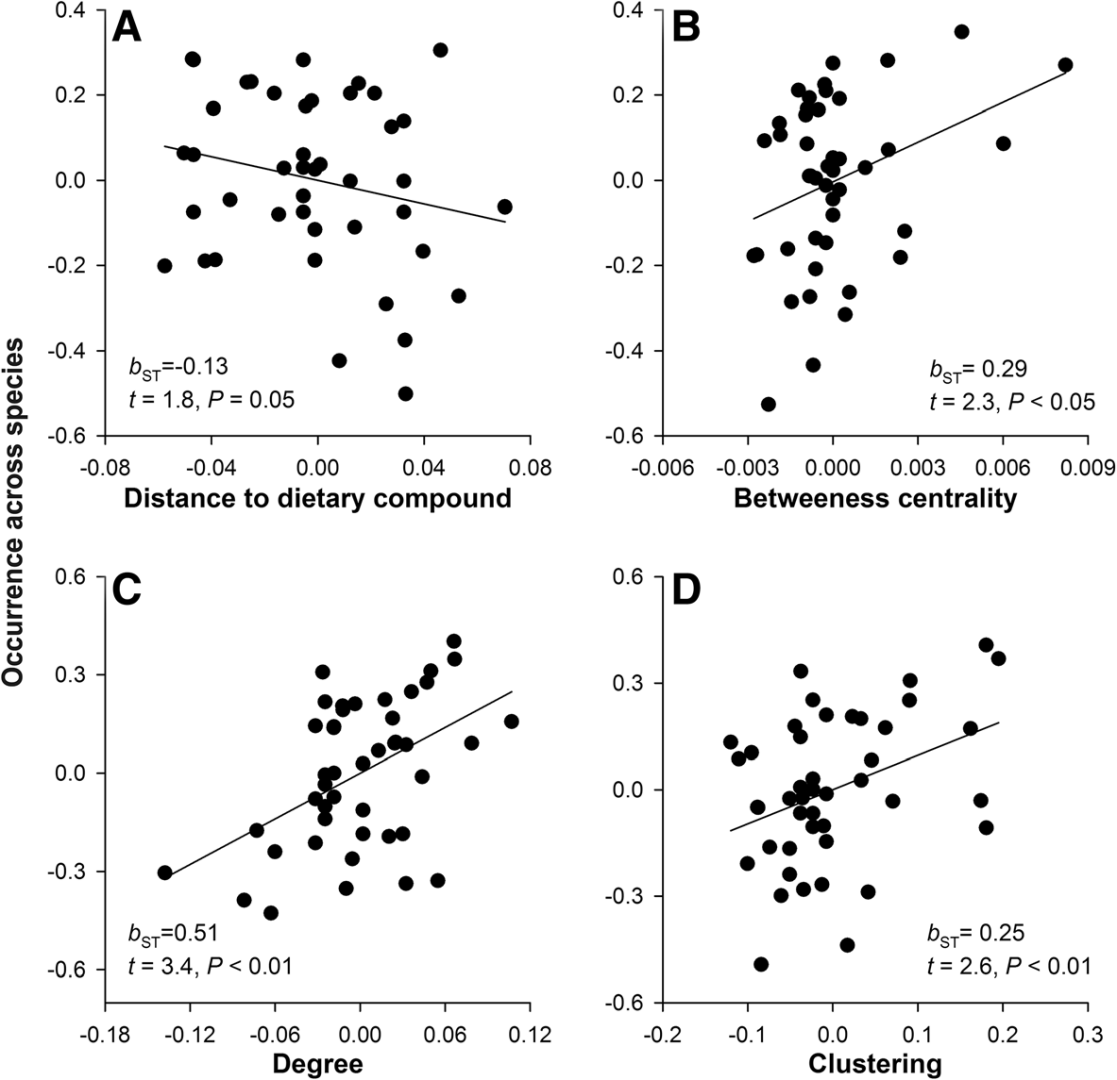
Carotenoid compounds that have fewer reactions from the dietary compound (**a**), have shorter (**b**) and more (**c**) enzymatic reactions to other compounds, and those that are parts of distinct clusters (**d**) are more common across bird species (occurrence across species is the node diameter in Fig. 4b). Shown are partial regression plots, *b*_*ST*_ is standardized regression coefficient (in SD)

Despite such apparent propensity for the loss, over the course of evolution, a majority of dietary and metabolized nodes in the combined avian carotenoid network were repeatedly occupied by many species (Fig. 6b). Estimated gains and losses of compounds in relation to length of metabolic pathway (Fig. 3a) and the role of dietary redundancy in sustaining elongation of metabolic pathways (Fig. 3b) both predict much more frequently occupied dietary nodes and limited diversification around these nodes. The pattern in Fig. 6b points to alternations of expansion and contraction in carotenoid networks across species [31, 32]. Indeed, occurrence of compounds across species was associated with their connectivity and metabolic distance to other compounds – carotenoids that had shorter pathways and more enzymatic reactions to other compounds and were fewer reactions away from additional dietary starting points (i.e., compounds with greater redundancy of enzymatic pathways by which they can be reached) were over-represented in birds (Fig. 7). Similarly, carotenoid compounds with greater enzymatic connectivity were the evolutionary “hotspots” of metabolic diversification in a sample of 330 bird species [29]. Importantly, the structural properties that predicted the compounds’ representation across bird species (and thus their presumed importance in avian evolution) derive from the compound’s topology on the ancient carotenoid network (Additional file 2: Figure S2, an abscissa in Fig. 7).

Metabolic network robustness is thus a central feature of avian carotenoid color elaboration (Fig. 3b and c) and such robustness is accomplished by acquisition of redundant dietary pathways.

### Evolution of association between metabolic elongation and dietary robustness

**Fig. 8:**
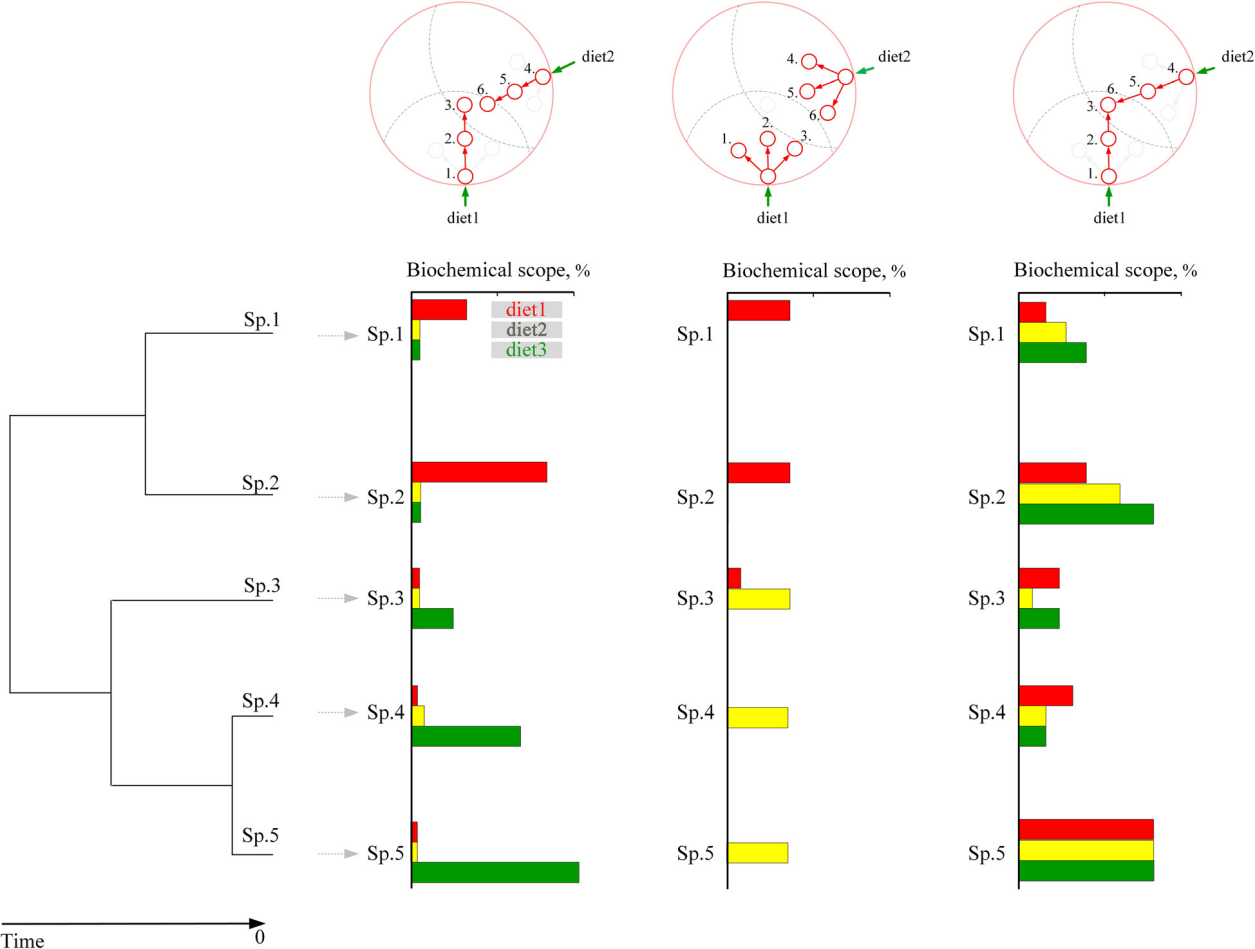
Hypothetical scenarios for historical association between elongation and robustness in carotenoid pathways. Biochemical scope is the total biochemical space (in compounds) potentially reachable from a dietary carotenoid based on its chemical connectivity, measured in the number of consecutive reactions (*numbered nodes in circles above*; Methods). *The metabolic elaboration* scenario (*left*) predicts stepwise colonization of biochemical scope and continuous elongation of metabolic pathway from *the same* dietary carotenoid within a lineage. Dietary entry points differ among lineages (Species 1-5), such that elaboration occurs within a biochemical scope of a particular dietary compound. *The exploration of dietary vicinity* scenario (*middle*) predicts limited elongation (small occupancy of biochemical scope) around dietary carotenoids. This scenario also predicts lineage-specific biochemical scope, but with less metabolic elongation. *The coevolution of elaboration and robustness* scenario (*right*) predicts that acquisition of additional dietary paths enables ongoing elaboration, such that within a lineage, longer pathways should be associated with sequentially gained paths to *new* dietary carotenoids, so that greater elongation is associated with greater diversity of biochemical scopes that contributes to it, even within a lineage

**Fig. 9:**
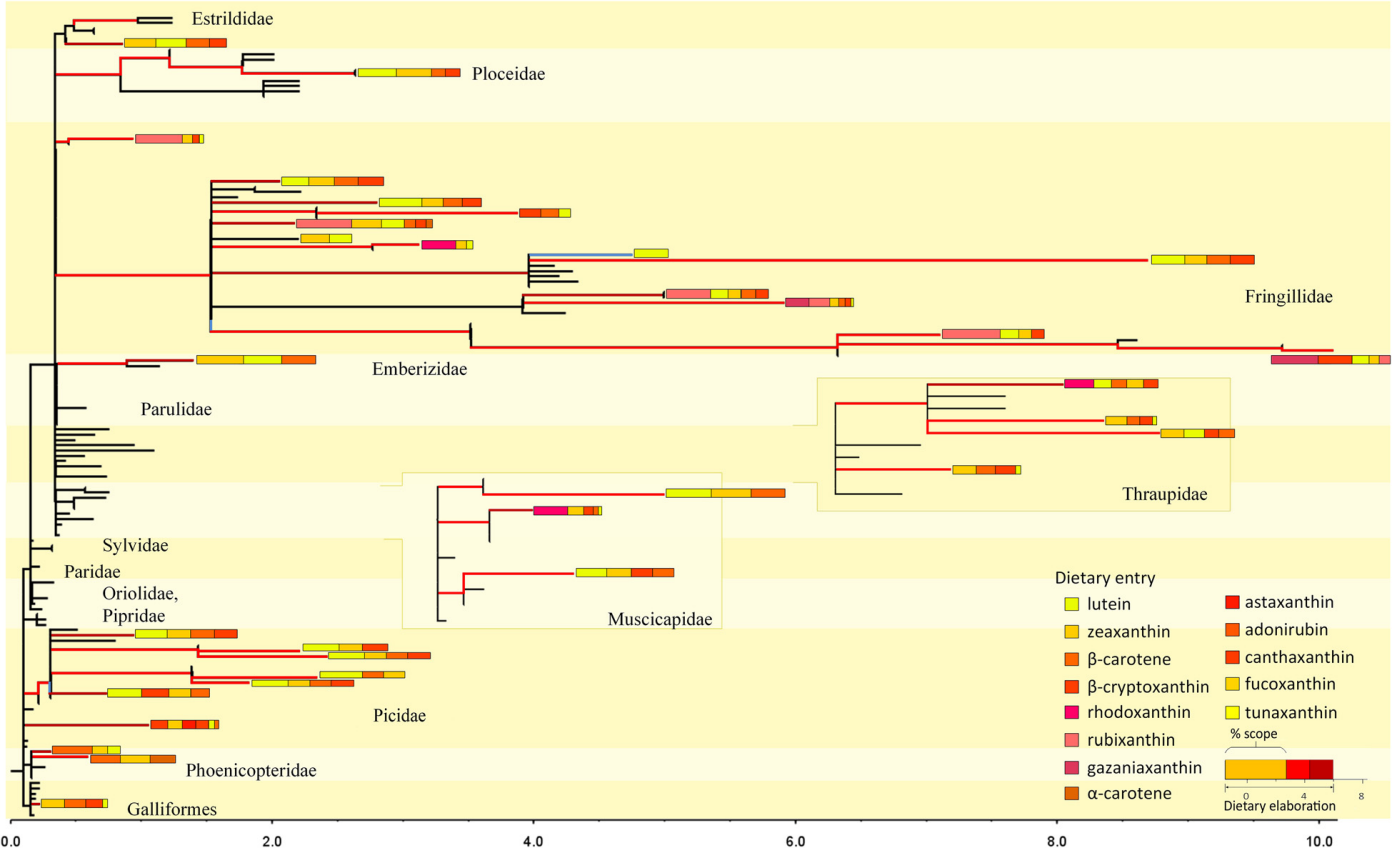
Sequential gains of new dietary paths “rescue” ongoing elaboration. Avian phylogenetic tree (Additional file 4: Figure S1) scaled to reflect the rate of carotenoid elaboration (reactions per million years). Most branches had negligible rates and are not shown. Branches colored according to the number of new dietary pathways gained (*red*), lost (*blue*), or retained the same (*black*) at each node. For species with accelerated metabolic evolution (*longer branch length*), the length of bar at tips indicates the longest path from dietary to plumage carotenoids (dietary elaboration, in number of reactions). These elaboration bars are partitioned by the exact ratios of biochemical scopes of distinct dietary compounds (shown by compound colors, legend) that contributed to dietary elaboration. Greater dietary elaboration was associated with greater number of similarly contributing dietary scopes, supporting the *coevolution of elaboration and robustness* scenario in Fig. 8, *right*). Thraupidae and Muscicapidae clades offset to shown details

Redundant dietary pathways are those that merge at a common internal node (Fig. 1a). We wanted to explore whether such merging influences the rate of carotenoid evolution, and whether it precedes or follows elongation of carotenoid-producing pathways within a lineage (Fig. 8). First, we calculated the rate of carotenoid elaboration across the avian phylogenetic tree (Fig. 9, Methods). Many branches within clades had negligible rates, reflecting either shrinkage of the metabolic network compared to more elaborate ancestors, or stasis. Nevertheless, in most clades, several species had strongly accelerated rates of metabolic evolution (in the upper 25 % of distribution, hereafter “highly elevated rates”). We then mapped gains and losses of *new* dietary (external) compounds within reconstructed networks at each phylogenetic internal node and found that a gain of a pathway from a new dietary compound subsequent to elongation (i.e., ancestral highly elevated rates) resulted in continuing elongation (i.e., descendant highly elevated rates), such that dietary gains, in essence, “rescue” ongoing elongation of pathways (Fig. 9; out of *n* = 33 transitions from ancestral highly elevated rates to descendent highly elevated rates, a new dietary compound was gained in *k* = 32 transitions, *z* = 5.22, *P* < 10^−4^). However, when a link to a novel dietary compound was not encountered, the descendant network either shrunk or remained static (i.e., descendant rates = 0) (Fig. 9, out of *n* = 49 transitions from ancestral highly elevated rate to descendant stasis or network reduction, all were associated with the lack of acquisition of an additional dietary link). Encounters of a compound that is connected to a new dietary entry point produced alternation of “boom” and “bust” events in the occupation of the avian network space (Fig. 9) – the pattern of recurrent occupancy of the combined avian carotenoid network noted above (Fig. 6b) *and* periodic convergence in plumage carotenoids (Fig. 2).

Thus, patterns of connectivity of carotenoid network are reflected in the likelihood of metabolic elongation. It follows, that when enzymatic connectivity in the vicinity of different dietary entry points vary, ecologically-distinct taxa should have different potential for color elaboration and diversification.

### Calibrating potential for metabolic pathway elongation across dietary entry points

Gains of dietary robustness (Fig. 9) can sustain ongoing metabolic elongation only when network structure enables the acquisition of a compound connected to a new dietary pathway in the smallest evolutionary step [2, 3, 5] (Fig. 1a). What is the maximum radius (in number of reactions) of the network space an evolving lineage can explore to encounter a beneficial compound whilst maintaining its current level of metabolic elongation? And do dietary carotenoids (and thus ecological groups of birds that utilize them) differ in potential of such gains?

**Fig. 10:**
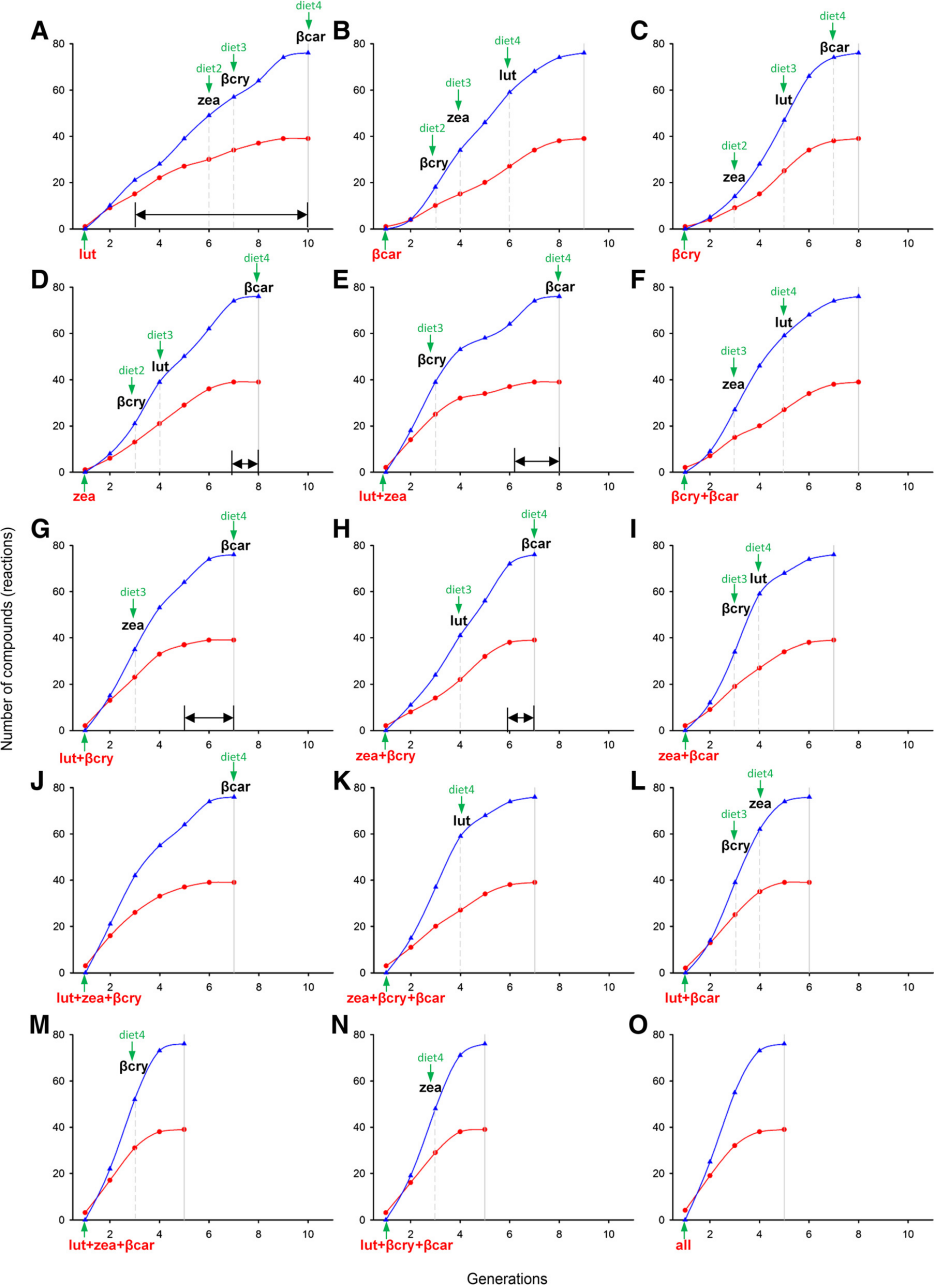
Biochemical scope [number of compounds (*red*) and reactions (*blue*)] for main dietary carotenoids and their combinations (*bottom, in red*). Dietary carotenoids (**a**–**o**) differ by which biochemical generation (simplest consecutive reactions) they reach an additional dietary compound (*green “diet” with arrow and dashed vertical line*) and the generation by which they encompass the entire avian carotenoid network space (*solid vertical line*). Horizontal double-headed arrows show areas that are inaccessible in avian evolution given the probability of losses for linear metabolic pathways (Fig. 3a). Main dietary compounds (lutein (lut), zeaxanthin (zea), β-carotene (βcar), β-cryptoxanthin (βcry) and their combinations are arranged, in ascending order, by the number of generations they require to encompass full avian network

To compare the potential for elaboration across external (dietary) compounds from purely structural considerations, we used a metric called “biochemical scope” [33] that reflects the number of simplest consecutive chemical reactions (called “biochemical generations”) needed to reach any compound in the network from a dietary starting point (Fig. 10). This measure reflects chemical connectivity of a focal compound [33].

For example, a sequence of chemical reactions starting with dietary lutein, will take ten biochemical generations to reach any compound contained in the combined avian carotenoid network (Fig. 10 and Additional file 2: Figure S2). However the first enzymatic pathway linked to additional external compound (to zeaxanthin in this case) is encountered only by generation seven (Fig. 10a), which means that the linear pathway has to be at least seven reactions long before it acquires dietary robustness (shown in red in Fig. 1a). In the context of avian evolution, such elaboration is highly unlikely given vulnerability rates of long linear pathways (Fig. 3a). Indeed, although 21 bird species in our dataset depended solely on lutein as source of plumage carotenoids (Additional file 3: Appendix S2), none had metabolic pathways longer than two reactions in their carotenoid networks (Fig. 3b). Such lack of enzymatic connectivity in the vicinity of lutein – the most common dietary precursor in birds (Additional file 3: Appendix S2; [29]) – might explain the overwhelming prevalence of yellow and greenish coloration across avian taxa. For avian species utilizing lutein as a *sole* dietary precursor (such as many warblers and sparrows, Additional file 3: Appendix S2), metabolic elaborations longer than three enzymatic reactions might be structurally inaccessible (Figs. 3b and 10a).

Among single dietary starting points, consuming zeaxanthin (Fig. 10d) enables the fastest and most sustainable colonization of avian carotenoid network: additional dietary compounds are encountered by 3rd and 4th generation). Only metabolic elongations beyond seven reactions are not accessible in the context of avian evolution. In species consuming two dietary compounds, a combination of lutein and β-carotene (Fig. 10l) enables reaching any compound in avian carotenoid network in only six generations, with encounters of additional dietary compounds by generations 3 and 4, enabling sustained metabolic elongation and diversification.

Applying the metric of biochemical scope across dietary entries and their combinations encompassing possible avian diets (Fig. 10) enabled us to explore in more details the relationship between dietary robustness and carotenoid elaboration (Fig. 9) across dietary entries that vary in enzymatic connectivity (Additional file 2: Figure S2).

We thus calculated the proportion of realized versus potential biochemical scope separately for each dietary carotenoid at each node of the phylogenetic tree (predictions on Fig. 8, partitioned bars at tips on Fig. 9). We did not find evidence for multi-node gains *within* a biochemical scope of a dietary entry (i.e., scenario 1 in Fig. 8), corroborating the finding that evolutionary diversification along carotenoid metabolic pathways is negligible in birds [29]. Exploration of a single-reaction radius of dietary entry (scenario 2 in Fig. 8) was commonly associated with stasis or network shrinkage, whereas in 32 out of 33 cases sustained elongation was associated with merging with an additional dietary pathway (scenario 3 in Fig. 8; results in Fig. 9). Strikingly, these patterns were observed across all dietary carotenoid domains (different combinations of dietary carotenoids at tip bars on Fig. 9) even though their enzymatic connectivity (e.g., compare Fig. 10a vs d) and likely color diversity (see below) strongly differed. These results, together with nearly identical outcomes of ancestral network reconstructions (Fig. 3a), and metabolic elongation in plumage carotenoids across study species (Fig. 3b), again show that network robustness is universally important in enabling carotenoid-based plumage elaboration starting from any dietary compounds (and thus, across diverse ecological groups of birds).

Interestingly, starting with several dietary entry points (versus sequentially gaining them) does not strongly accelerate metabolic evolution as evidenced by relatively short branches in some seed-eater finch lineages that start with several dietary compounds because of their carotenoid-diverse diet, but do not consistently gain new ones (Fig. 9, Additional file 3: Appendix S2). For example, starting carotenoid metabolism with β-cryptoxanthin, zeaxanthin, and lutein – a common combination in Cardueline finches (Additional file 3: Appendix S2) – enables particularly sustainable evolution of plumage elaboration and diversification (Fig. 10j). Indeed the two species in the dataset that accomplished the greatest elongation of carotenoid pathways (two reactions less than maximum theoretically possible distance from any dietary entry on the avian carotenoid network, Fig. 3b) occurred in this group (two longest branches in Fig. 9). Red Siskin (*Carduelis cucullata*) and Pallas’s Rosefinch (*Carpodacus roseus*) combine exceptional metabolic elongation (>6 metabolic steps from a dietary entry) with strong robustness (>4 different dietary pathways sustaining such elongation); reconstructing metabolic networks on internal phylogenetic nodes leading to these taxa revealed that these lineages consistently gained a new dietary compound at each of the last 4 and 5 phylogenetic transitions, correspondingly (Fig. 9).

Carotenoids are ancient compounds, involved in a multitude of biological functions in addition to coloration (e.g., [34]). Most elements of carotenoid enzymatic pathways have likely evolved in the context of bacterial evolution and were in place millions of years before animals and plants started to use them in coloration and other functions [9, 10, 35]. The central assumption of this study is that, in birds, the evolution of color-producing metabolic pathways involves cooption, duplication, and linkage of compounds already present throughout tissues, their transport, and their integration with feather growth [24, 36], rather than evolution of these specialized pathways de novo in each lineage (but see [24, 37]). There are no data to test this assumption currently, but it is corroborated by similar genomic profiles of carotenoid enzymes in tissues of differently colored sexes and species (e.g., [38, 39]) and by frequent and rapid convergence in complex carotenoid pathways among distantly-related taxa (Fig. 2, Additional file 5: Figure S3).

In this study we did not explore correspondence between carotenoid producing metabolic network and carotenoid-based color space in birds. Three factors can affect such correspondence. First, plumage colors and metabolic elongation can differ across network areas – in some enzymatic sequences a single reaction step produces a different color, whereas in others more steps are required for the same color change (e.g., [9]). Similarly, some metabolic neighborhoods can underlie greater color diversity than others. Second, metabolic pathways commonly differ in their efficiency (e.g., [40]); relative concentration of compounds produced by different pathways within species’ network and mixtures of different carotenoids and distribution of flux [41] further alter correspondence between metabolic and plumage color space. Third, plumage color is affected by integration of carotenoid compounds and feather keratin proteins and species differences in this integration [42, 43] changes correspondence between metabolic and color diversification space across species. However, the structural trade-off between metabolic robustness and elongation in carotenoid pathways identified in this study is central to most of the above-mentioned mechanisms as well. This is because robustness to fluctuations in external inputs enables carotenoid elaboration by extending time available for its evolution (Fig. 3a and b) and this equally applies to the evolution of metabolic pathways (as documented in this study, Fig. 9), their enzymatic efficiency (e.g., [44]), or the time required for the evolution of species-specific feather protein binding [45, 46]. Indeed, the evolution of carotenoid-protein associations in growing feathers was proportional to the time a lineage was coevolving with a particular carotenoid compound in a subset of species from this dataset (Badyaev, Potticary, Morrison, ms). Nevertheless, explicitly characterizing and contrasting the relative contribution of structural considerations identified in this study with metabolic flux and dynamic properties of avian carotenoid networks (including experimentally confirming assumed directionality of enzymatic reactions in avian networks) is a necessary next step in understanding the tempo and mode of avian carotenoid evolution.

## Conclusions and implications

Our results have several implications. First, the structure of underlying metabolic networks mechanistically links microevolutionary elaboration and macroevolutionary diversification in avian carotenoid coloration. Such linkages do not depend on specific evolutionary force: for example, metabolic elaboration within lineages can be driven by sexual selection [47, 48] whist lineage diversification can be due to random sampling of neighboring enzymatic reactions, or, conversely, ecological speciation (and associated diet specialization) can dictate lineage divergence [49–51], but subsequent within-lineage evolution can be driven by the cooption of enzymatic reactions available in the immediate biochemical vicinity of dietary compound (e.g., [29, 52]). Second, the evolutionary potential for carotenoid color elaboration and diversification should differ predictably among clades depending on the density of enzymatic reactions, overlap in biochemical domains of distinct dietary carotenoids, and diversity of dietary carotenoids in species’ metabolic networks (e.g., [53]). Third, placing ecological and biochemical diversification in the same phylogenetic framework provides an opportunity to directly compare their contribution to the evolution of carotenoid ornamentation in birds and contributes to the conceptual unification of functionalist and structuralists views of evolution.

## Methods

### Phylogenetic tree

We retrieved cytochrome *b* (cytB) sequences for 181 species in the dataset from GenBank [54] (Additional file 9: Table S3). Twenty five species without cytB sequences were represented by congeners with sequences, inserted at their respective topological positions in the supertree. Sequences of *Fregata minor* and *Zosterops japonicas* sequences were trimmed to include only cytB gene before alignment using MUSCLE [55] with default parameters. To estimate branch lengths on the supertree [56, 57], we fitted these cytB sequences to the supertree topology using maximum-likelihood in RAxML [58].

The tree (Additional file 4: Figure S1) was built by supertree methods by adding 24 molecular phylogenies containing the focal species (Additional file 10: Table S4) onto a backbone phylogeny [59]. Because no complete molecular phylogeny exists for Phoenicopteriformes, a phylogeny based on DNA-DNA hybridization [60] was inserted. A morphology based phylogeny [61] was used for species in the family Pipridae. Given the large number of species in the dataset in the order Passeriformes and the family Picidae, we inserted additional backbone molecular phylogenies of these groups [62, 63] into the overall supertree at the root of their respective clades.

A combination of seven molecular clock calibrations, four fossil calibrations, and one paleogeographic event calibration were used to convert the supertree into an ultrametric tree in PATHd8 [64]. We used as many available calibration points as possible because this is the most accurate method to determine the rate variation across trees that include distantly related taxa [65]. The basal split in Neoaves was fixed at 100 MYA; although this is one of the higher estimates from molecular clock studies [66], estimates range from about 65 MYA [67] to 123 MYA [66] and it has been used elsewhere as a calibration point [68]. Divergence estimates are sensitive to taxon sampling [69] contributing to uncertainly of divergence time among higher orders [70–72]. Three additional molecular calibration points were from the molecular clock analysis of complete mitochondrial genomes of Neoaves orders [73]. These points were assumed to be fixed in the analysis and include the divergence between the sister suborders of suboscines and oscines at 73.61 MYA, the divergence between Threskiornithidae and Pelecaniformes at 66.0 MYA, and the diversification of the Charadriiformes stem group at 66.57 MYA. The molecular clock estimation of 12.0 MYA was used as the fixed divergence time between the sister genera *Campephilus* and *Picoides* in the family Picidae [63]. A fixed point of 20 MYA from the molecular clock analysis [62] was used as the date of the most recent common ancestor of the genera *Fringilla*, *Emberiza*, *Thraupis*, *Cardinalis*, and *Icterus*. Finally, 13 MYA was assigned as the fixed diversification time of the *Oriolus* stem group, based on a molecular clock analysis [74]. The fossil calibration points consisted of a fixed point at 66 MYA for the age of the Anseriformes stem group [75], a range of 30-34 MYA for both the ages of the Pici crown group [76] and the diversification of the order Phoenicopteriformes stem group [77], and a fixed point at 53 MYA for the age of the Galliformes stem group [78]. In order to have more calibration points within the large Passeriformes clade, we used calibration points of 4.8–5.5 MYA as the range of dates of the Regulidae stem group, based on paleogeographic evidence [79, 80].

After the ultrametric tree was constructed, the congeners with cytB sequences that were inserted in the tree for species without cytB sequence were pared from the tree at the genus level and the focal taxa were inserted in their place so that the branch length of the genus’s root node was retained. Polytomies with branch length values of zero were constructed for genera that consisted of multiple focal taxa without cytB sequences. The final ultrametric supertree included 152 species (Additional file 4: Figure S1).

#### Comparison with Jetz et al. [81] avian supertree

To examine the similarity of our molecular supertree with a recently published global avian supertree [81], we compared the topology of both trees for the subset of species used in this study. A random sample of 1000 trees containing all of our study species was obtained from the Bayesian pseudo-posterior distribution of 10,000 time-calibrated trees from associated website (birdtree.org) [81] based on the backbone [59] with all 9,993 species. A 50 % majority rule consensus tree was generated from the sample of the 1000 trees from the pseudo-posterior distribution in R 3.1.0 [82] using the package APE (ver. 3.1-1) [83]. To identify which clades were supported in both [81] and our supertrees, we generated a strict consensus tree between the majority rule consensus tree of the Jetz et al. supertree and our supertree (Additional file 4: Figure S1) using the package APE (ver. 3.1-1). All higher-level (family or orders) and most within-family and within-genera relationships were retained in the strict consensus tree, signifying topological congruence in these clades in both [81] and our supertree. The only difference between the trees was a subset of 12 species within the Fringillidae, that collapses into a polytomy in the strict consensus tree.

To quantify the number of clades found in our supertree that are not found in the strict consensus tree based on both our supertree and the [81] supertree we calculated the Robinson-Foulds distance (RF) [84] between the strict consensus tree and our supertree with *phangorn* (ver. 1.00-7) [85]. The RF distance between our supertree (Additional file 4: Figure S1) and this strict consensus tree was 31 out of a maximum possible distance of 298 for 152 species, also indicating concordance of topologies.

Further, both trees are based on the same backbone phylogeny for avian orders [59] and the same backbone phylogeny for Passeriformes [62] – the majority of species used in this study. Additionally, both trees share two of the oldest fossil calibration points: at the stem Pici for the split between Ramphastidae, Picidae and Indicataoridae (30 MYA [76]), and at a basal node at the stem for Antidae (66 MYA [75]). Further, additional 10 calibration points on our supertree were specific to the clades under this study, thus giving us a more accurate divergence time estimates than would be available from the subsample of the study species in global avian supertree [81].

#### Comparison of the Hackett et al. [59] molecular phylogeny to the Jarvis et al. [67] genome-scale phylogeny of avian orders

To further assess the validity of the use of the Hackett et al. [59] phylogeny as a backbone tree in building our supertree, we examined the similarity between the relationships of avian orders on the backbone tree derived from 19 nuclear genes by Hackett et al. [59] to relationships of these orders on the recently published genome-scale phylogeny of all orders of Neoaves [67]. Analyses by Jarvis et al. [67] comparing the overall topology of both [59] tree and their own total evidence nucleotide tree (TENT inferred with ExaML) found that out of all of the previously published relationships in avian phylogenies the [59] phylogeny was the most congruent with the genomic scale phylogeny. They both recovered the same monophyletic clades of core landbirds and core waterbirds. The core landbirds were also divided into the same two monphyletic clades in both phylogenies. A comparison of the two trees only in relationship among the eight main orders present in our supertree (Anseriformes, Galliformes, Phoenicopteriformes, Pelecaniformes, Charadriiformes, Passeriformes, Piciformes, Accipitriformes) revealed that all of the relative relationships between the orders were identical except for the placement of Charadriiformes. In the Hackett et al. [59] tree, the order Charadriiformes is more derived than Pelecaniformes while in the Jarvis et al. [67] tree this relationship is reversed. Of our 152 taxa under our study, only seven (4.6 %) come from these orders. Given this minor difference and for the reasons specified above, we retained the Hackett et al. [59] phylogeny as the backbone tree for our molecular supertree.

### Carotenoid biosynthesis network

#### Global carotenoid biosynthesis network

Although complete and experimentally verified carotenoid biosynthetic networks are known for only a relatively small number of species (Additional file 1: Appendix S1 and references therein), these represent a subset of a global carotenoid network of known biochemical transitions among the compounds [17, 86]. Thus, as a first step, we constructed a global carotenoid biosynthesis network that included all confirmed enzymatic reactions that occur among naturally occurring carotenoids (Additional file 1: Appendix S1). This global carotenoid biosynthesis network delineated entire biochemical space and pathways available for carotenoid diversification based on biochemical properties of the compounds.

#### Avian plumage carotenoid biosynthesis network

As a second step, we collected an exhaustive list of all carotenoid compounds and reactions known in birds (*n* = 248 species, current to May 2014; for dataset updated to July 2015 see appendices in [29]), including compounds found in plumage, integument (bill, tarsi, skin), plasma, liver, fat, faeces, and seminal fluid, or known to be consumed (Additional file 3: Appendix S2). All these compounds and reactions were used to construct the “avian subset” of the global carotenoid network (Additional file 2: Figure S2, Figure 6a) – combined network of carotenoid reactions found in bird species.

As a third step, for the plumage carotenoid-producing pathway of each species, carotenoid compounds were classified as *dietary* if they are known to be consumed with diet, *intermediate* (metabolized) if (i) they are not expressed in the plumage, but found in plasma, liver, gut, fat, or (ii) known to be metabolized on the path from dietary to plumage compounds, and *expressed* if they are found in plumage (Additional file 1: Appendix S1). Dietary, intermediate, and expressed compounds, and enzymatic reactions among them were linked into a metabolic network for each species and then mapped on the global carotenoid network. When data were available, separate networks were built for male, female and juvenile of the species and the fullest networks for each species were used in subsequent analyses.

In the majority of species, known enzymatic pathways and their directionality inferred from global carotenoid network unambiguously linked identified compounds in carotenoid-producing pathways (Additional file 1: Appendix S1). In several cases (see below), we added an intermediate compound (i.e., a biochemical reaction step) to link observed dietary and expressed compounds when these were located on the same linear pathway or extended a path from an expressed compound to a dietary starting compound if both could be unambiguously linked by a reaction step. When more than one dietary compound was recorded for a species, these were not linked by direct enzymatic pathways (even when one existed on the global carotenoid network – e.g., in bacterial networks) unless such direct linkage was experimentally confirmed for the focal species.

To form a color-producing pathway, birds can either link dietary, intermediate, and expressed compounds present in each species in a linear, step-wise fashion [24, 87, 88], utilize the diversity of redundant pathways that link them [29, 40], or use a mixture of these strategies. Thus, to make an unbiased assessment of the effect of network structure on avian carotenoid diversification, we built two kinds of networks for each species – a “minimum” and “maximum” networks. The minimum network represented the minimum number of reactions required to produce the plumage compounds from dietary carotenoids of each species. To obtain the maximum network, we mapped compounds recorded for the species on the “avian space” of global carotenoid biosynthesis network (Additional file 2: Figure S2) and recorded all biochemically possible reactions that could produce the plumage compounds identified in each species from dietary and intermediate compounds for this species (Additional file 3: Appendix S2). We used both types of networks in the analyses and report when results differed. “Minimum” network was used in descriptive analyses and tests. Considering both minimum and maximum networks, with redundant pathways included, allowed for explicit tests of mode of evolution of avian carotenoid networks on known biochemical network. The use of maximum networks in ancestral reconstructions (see below) provides a particularly conservative test for the effect of network structure on color diversification. Limited genomic data suggest that species possess many more types of carotenoid compounds and enzymes in their tissues than those involved in their color-producing pathways [38, 39], such that evolution of developmental pathways by which feathers are colored likely involves coopting and linking compounds and reactions present in species throughout organismal tissues, a process approximated in this study by the use of the maximum network measure.

### Ancestral networks reconstruction

Ancestral network reconstruction in an explicitly phylogenetic context requires evaluations of structural relationships and biochemical rules by which the compounds and enzymes are linked in the networks of closely-related species and reconstruction of ancestral networks by either parsimony [89–91] or maximum likelihood approaches [92–94]. A previous approach to reconstruction of Bayesian networks [95] infers the most likely evolutionary scenario for each metabolic reaction present in the extant species, but only tracks the gain and loss of reactions and assumes a fixed number of network compounds. To overcome this limitation, we used a modified maximum likelihood approach [91] to test the fit of models of network evolution in the phylogeny and to track ancestral states of compounds and reactions simultaneously (Additional file 7: Table S2). We further extended this method to reconstructions of the ancestral states of reactions (and thus the phylogenetic changes in the overall topology) [96, 97]. Following the reconstruction of ancestral states, we built ancestral networks at each of the internal nodes on the phylogeny [90, 91, 95] based on the compounds and reactions present at this node, and then calculated their associated network parameters (Additional file 8: Table S1). The presence or absence of compounds and reactions was recorded in a character matrix for 159 species (Additional file 7: Table S2). We used *r8s* (version 1.8) [96, 97] to obtain maximum likelihood estimates of the rate parameters for compounds and reactions (Additional file 7: Table S2).

### Phylogenetic analysis of metabolic networks and pathways

#### Distance measures

To quantify structural distance between species’ metabolic networks we used a modified version of the metabolic distance [98] that measures the fraction of reactions and compounds in which any two networks differ. We calculated pairwise distances between each of the species’ metabolic networks and used the neighbor-joining algorithm [99] in PAUP* 4.0 [100] to build a phylogeny based on metabolic distance between species (Fig. 2). Evolutionary distance between the species was the sum of branch lengths on the shortest phylogenetic path between any the two species (patristic distance, in millions of years) [101].

#### Comparison of the metabolic pathway-based tree and molecular-based supertree

To determine the topological similarity between the metabolic pathway-based phylogeny and the molecular-based supertree we used the Robinson-Foulds metric (RF) [84, 102], in R 3.1.0 [82] using the package *phangorn* (version 1.99-7) [85]. Phylogenies with identical topologies have an RF distance of 0, and the maximum RF distance for a data set of *n* taxa is 2(*n*-3) [103]. The likelihood of the RF distance was simulated with 10,000 random stochastic birth-death trees for *n* = 152 species using the *phytools* (version 0.3–93) [104].

## Reviewers’ reports

### Reviewer 1: Junhyong Kim, University of Pennsylvania

In this paper, the authors address a classic question in evolutionary biology: if some distinct trait distribution of organisms is seen, how much of the pattern is due to the distribution of fitness peaks and historical contingency and how much is due to constraints like the set of developmentally reachable phenotypes. We might imagine a spread of people in a landscape of multiple hills and mountains, trying to climb up; but, the landscape also has cliffs and gorges that constrain travel routes. If we take a snapshot of the people, how much of their distribution pattern is due to the location of the hills, their ability to walk, and the location of the forbidden obstacle? Some authors might argue that excluding constraints of physics and chemistry, such questions are really a question of time scale. What is reachable by certain developmental processes can change as the mechanisms of development itself can evolve – that is, the constraints themselves can change over time. Nevertheless, the question, originally made famous by late S.J. Gould, is both conceptually interesting and significant for proper inference of empirical phenotypic distributions.

The current paper has some very strong points in its attempt to address evolutionary questions arising from the above framework: (1) it addresses a deep and important question in macroevolution using approaches that leverage a unique information set; (2) this information set, consisting of biochemical reaction network of carotenoid, is marvelously researched and it is truly fascinating in terms of both its appropriateness and relevance to an phenotype (coloration) of great interest; and (3) the many numerical methods applied throughout the paper are carried out with extremely high degree of technical competency and attention to detail. I believe this paper should be published but with some major revisions.

While the paper has many excellent points of strength, unfortunately, I found the paper very difficult to read and in many parts how the results tie to the models or hypotheses were cryptic. With respect to reading difficulty, I believe some of this is perhaps due to the manuscript addressing a specialized audience that might be already familiar with some terms or concepts but not a more general audience. For example, the paper uses the term “diversification” and “elaboration” – presumably in a specific manner, but to a general reader it is not clear what pattern of data would be considered diversification versus elaboration. I suspect such a reader would stumble at phrases like “…structural consideration of trait construction and development or selective environment of trait functioning over historical time.” It would greatly help the manuscript to revise the text for more general audience. I personally stumble on phrases like “components of current adaptations”, as I do not know whether “components” refers to a specific technical term or whether it simply means things other than genes. The manuscript has a large number of such ambiguous terms: e.g., “structural opportunities”, “elongation of pathways”, “metabolic elaboration”, “biochemical scope”, “biochemical space”, etc. I think each such term needs to be defined for the reader.

Authors’ response: *We took this opportunity to edit and clarify the text and eliminate excessive jargon. We made consistent the use of “metabolic elongation” and “plumage elaboration” and now define several terms in the text more explicitly and delete others. Established measures from network biology and biochemistry are listed in Additional file**8**: Table S1.*

As mentioned, some of the results are cryptic or it wasn’t entirely clear what hypothesis was being tested by the analyses. For example, on page 5, it states that the hypothesis being tested is whether the biochemical pathway context explains the patterns wherein “closely related species are often strongly diverged…distant and ecologically distinct taxa are often highly convergent.” I am not sure which of the results are directly addressing this problem. Some of this problem may again be due to the writing and my confusion.

Authors’ response: *We now rewrote this introductory paragraph to emphasize the general approach of the study: whereas rapid divergence among related species (and frequent convergence among distant species) in plumage carotenoids is commonly documented, here we proposed a novel explanation: recurrent occupancy (*i.e.*, sharing) of a small biochemical space. We specifically address the reason and calibrate the rate for such recurrence for different ecological groups.*

For example, referring to Fig. 1c, the text states “We found that diversification in plumage ….by frequent bouts of metabolic (internal) diversification… and not by ….starting points.” I do not understand from what parts of Fig. 1c I can also come to the same conclusion. That is, the figures need more explanation was to why such a figure was made and how the data in the figure leads to the conclusion suggested by the authors. I find conclusions drawn from Figs. 2c, 3a, 4a–d, 5, 6, and 7, similarly cryptic.

Authors’ response: *We now expanded both the figure legends and associated text explanation and more explicitly refer to comparisons of panels in Fig.*1*. The conclusion mentioned here refers to comparisons of the upper (dietary subnetwork), middle (metabolized subnetwork) and lower (expressed subnetwork) panel of Fig.*1*c for the same time period (common x-axis for all panels). Figure shows that network divergence of species separated by <20 million years is the largest in metabolized compounds (middle panel), but the smallest in dietary compounds (upper panel). We further clarified presentation of results and made it less abbreviated by simplifying Figs.*3*and*4*(panels are now presented as separate figures) and brining two figures from Additional Files to the main text to streamline the presentation.*

There are some concepts or measures that are not explained sufficiently: How is “robustness” defined? What is meant by robustness to fluctuations? How is evolutionary persistence defined?

Authors’ response: *Robustness is now defined in both the Background and also in figure legends. Evolutionary persistence was referring to occurrence across distant species and is now rephrased and deleted from figures.*

How does Additional file 5: Figure S3 and Fig. 3a result in the conclusion that there were “periodic alternations of expansion and contraction … in frequencies broadly consistent with both estimated gains and losses … and with the role of dietary redundancy …” I think this is an important model and it seems to make sense. However, it is difficult to see from the given results whether this is, in fact, a precise and quantitative statement. For example, what parts of the results suggest this model predicts frequencies consistent with estimated gains and losses?

Authors’ response: *We now show former Additional file**5**: Figure S3 and Fig.*3*a as panels to facilitate comparison. New Fig.*6*b shows that most nodes (not only dietary ones) are represented in many species and most nodes are occupied by species under this study. Frequencies of gain and loss in relation to distance from dietary entries in Fig.*3*a shows that long pathways are unlikely to persist unless, as predicted in Fig.*1*a and*3*a (insert), metabolic pathways acquire additional link to dietary compounds. When such evolutionary acquisition is possible, given local enzymatic connectivity, pathway can elongate and more nodes get inflated in Fig.*6*b. When such acquisition is not possible, nodes get smaller. Figure*6*b is necessarily a qualitative illustration of outcome of such expansion-contraction process. Quantitative results for the depicted process are presented in Figs.*8*and*9*.*

In sum, this is a paper of unusual depth, technical sophistication, and scholarly breadth. However, the presentation is difficult and at places the models and hypotheses are hard for the reader to derive from the presented results. That is, the conclusions drawn from each figure or results table may be obvious to the authors but a reader is likely to have much harder time. Given the potential impact of the paper it would be a great service to the readership to clarify the presentation. Lastly, given that the results from this paper is supposed to explain, at least in part, the spectacular diversity and distribution of bird coloration, it would really help draw interest to the paper to graphically show to the reader the implications from the models to the macrophenotype of popular interest.

Authors’ response: *We greatly appreciate Dr. Kim’s assessment of this work and did our best to clarify the presentation. Some of the results of this work lend themselves for extrapolation into actual color distribution in birds. For example we mention why yellow and green are the colors of stasis in avian pigmentation evolution and also an ancestral state in many lineages. Further, we suggest that prevalence of losses over gains in avian history can be related to a combination of selection for exaggeration of color displays (and associated selection for efficiency of pigment production and their modularity) and birds’ dependence on upstream carotenoids they cannot themselves convert from non-carotenoids. This combination leads to formation of isolated modules that are frequently lost in avian evolution. However, as we discuss in the paper, direct correspondence between enzymatic pathways and expressed plumage color varies across the network.*

In my previous review, I noted the significance of this paper and the unique and novel analyses applied to a singularly interesting dataset. The paper addresses a topic of great conceptual depth and overall conclusions seem to be supported by the data. However, I still had great trouble understanding individual pieces of the analyses and how the presented figures relate to the verbal descriptions given in the paper. Here, I list in more detail the particular problematic sections. I hope the authors will address and clarify these sections such that the reader can appreciate the results.

Line 125: “Metabolic divergence”—it would really help the reader here to state in a few words how the authors measured metabolic divergence rather than only in the methods section. Line 126: “Periodic convergence” – Fig. 2 is a large complex tree, side-by-side. As a reader, I cannot pick out where these convergence events is evident on these two trees and why such events would be “periodic.” Figure 1c: Axis labels are ambiguous. Is the Y-axis supposed to be the slope coefficient? That is, the slope of a subset of the points in 1B (different compound categories and within specific intervals of the X-axis of 1B?). Line 139: It is not clear to the reader how a “structural basis” for the phenomenon of “puzzlingly rapid divergence of closely related species in expressed carotenoids” is found in the results presented in Fig. 1c. The last panel seems to show high level of divergence in closely related pairs but it isn’t clear why the figure is supposed to show this “structural basis”. Line 157-158: Where in the figure can the reader see that “combined time required for evolving…four metabolic reactions” is comparable to the age of birds? Figure 5: The figure legend is very sparse and needs more explanation. What are each dot in the figure? Axes should also be explained in the legend. Line 190: Where is the information for “linearity” of the network? Line 202—205: Here, it is suggested that the Fig. 4b is consistent with alternations of expansion and contraction, etc. However, it is very difficult to see this. What is the expected pattern if some other hypotheses were posed? For example, if all species consistently expanded their network? Or, if there were only expansion for some species and contraction for others?

Line 221: The legend for Fig. 8 is difficult to understand and perhaps incomplete. (A typo in line 982 of the legend “biological scope” should be “biochemical scope”.) Line 985 of the legend states “significant elaboration is mostly produced within a biochemical scope.” After reading the description and seeing the inset diagrams in Fig. 8, it is still not clear to me what aspects of the figure is being called “elaboration” and “biochemical scope”. That is, I can see the diagram and understand that there are model differences but the translation of these scenes into phrases like “greater elongation is associated with greater diversity of biochemical scopes” is not clear to me. The concept of “biochemical scope” seems critical to understanding this result as well as the results in Fig. 9. Lastly, this figure contains additional information such as the tree and the bar graphs. The legend does not give information about these additional items.

Line 280: What is “proportion of gained biochemical scope”? Is this the proportion of the metabolic network realized versus reachable in X number of generations?

Authors’ response: *We now clarified our references to the figures and made necessary corrections.*

### Reviewer 2: Eugene Koonin, NCBI, NIH

Badyaev and colleagues explore a truly interesting evolutionary landscape where evolution of a metabolic network is intimately linked to the evolution of a highly visible phenotypic trait, the avian plumage coloration. The analysis is quite elaborate, and the conclusions, in particular, the explanation for convergent evolution of carotenoid pigmentation, seem important.

Yet, I have to confess that, at least to me, the manuscript is difficult to read, and I was unable to follow all the steps that led the authors to these conclusions. I wonder whether it might be possible to explain the connection between metabolic robustness and elaboration in simpler terms and/or in greater detail, perhaps using some diagrams to facilitate understanding. Also, it is not immediately clear to me how and where does this paper address the (dis)connect between microevolution and macroevolution.

Authors’ response: *The diagram showing how elongation of metabolic pathways is structurally linked to robustness is shown in Fig.*1*a. We now more explicitly state that it is birds’ dependence on dietary (*i.e.*, external) carotenoids to initiate their metabolism that results in this property. Importantly, the link between metabolic elaboration and dietary robustness does not depend crucially on fluctuations in availability of different dietary carotenoids in environment, but rather refers to the fact that these compounds are external to the organism and thus have different evolutionary dynamic.*

*The link between micro- and macroevolution in this study comes from bringing together the mechanisms behind changes in metabolic pathways within taxa or lineage (*e.g.*, sexual selection for more elaborate or efficiently produced carotenoid-based ornament) with the patterns of interspecific diversity in these pathways (*e.g.*, frequent convergence across distantly related species, alteration of elongation and stasis in these networks on avian phylogeny). We bring these micro- and macroevolutionary processes together by considering them on the same structural landscape – the enzymatic connectivity in global carotenoid network. Diversity of avian species, their long history, and the relatively small size of carotenoid network on which they diversify also enabled robust ancestral reconstruction of their metabolic networks. This enabled us to consider ecological and biochemical processes within the same phylogenetic framework; for example, comparing contribution of ecological and metabolic components to the evolution of avian carotenoid pigmentation (Fig.*1*c)*.

Furthermore, would it be possible to explain, in specific terms, how do metabolic networks expand in birds. Given that birds do not appear to be prone to horizontal gene transfer, I suppose that this expansion involves gene duplication followed by neofunctionalization. Is this correct? Would it be possible to present specific cases? I think this would clarify the entire story. The situation with losses is more obvious but again, a specific description at the level of genes and enzymes would be helpful.

In short this is quite a substantial paper on an interesting subject but I think it could gain a lot from a more complete and specific presentation.

Authors’ response: *It is very good point and this, indeed, would clarify the entire story. From a companion study involving larger sample size (n = 330 species,* [29]*) we know that, structurally, carotenoid-producing networks in birds expand at most connected enzymes – these nodes are the “hotspots” of phenotypic diversification in carotenoid network* [29]*. But there are currently no empirical data to test the correspondence between genomic and metabolic networks for carotenoid production across birds. Avian gene duplication rate* per se *seems to insufficient* [39] *to account for the fast rate of convergence in carotenoid enzymes across distantly related bird species documented in this study (Fig.*2*and Additional file**5**: Figure S3), although it is very likely that neofunctionalization at genomic or metabolic levels plays a major role. The finding of strongly reductive evolution of carotenoid-producing pathways (Fig.*5*) corroborates this assumption. The hypothesis advanced in this paper also explains the prevalence of reaction losses during evolution of avian lineages.*

### Reviewer 3: Fyodor Kondrashov, Center for Genomic Regulation and University of Pompeu Fabra

This reviewer provided no comments on this version for publication.

## Additional files

Additional file 1: Appendix S1. Confirmed enzymatic reactions in “avian space” of global carotenoid biosynthesis network in bacteria, plants, and animals. References for Appendix S1. (PDF 219 kb) Additional file 2: Figure S2. Avian “subset” of global biochemical network of carotenoid metabolism. (PDF 225 kb) Additional file 3: Appendix S2. Characteristic of carotenoid metabolic networks for species used in this study. References for Appendix S2. (PDF 205 kb) Additional file 4: Figure S1. Molecular supertree for bird species under this study. (PDF 196 kb) Additional file 5: Figure S3. Distribution of Robinson-Foulds distances between 10,000 random trees and the molecular-based supertree. (PDF 128 kb) Additional file 6: Figure S4. Diversity of characteristics of metabolic networks producing plumage carotenoid across bird species under this study. (PDF 110 kb) Additional file 7: Table S2. Estimated gains and losses of carotenoid compound in relation to their structural position in the network. (PDF 156 kb) Additional file 8: Table S1. Measures of carotenoid metabolic network used in the study. (PDF 157 kb) Additional file 9: Table S3. GenBank (GI) and taxonomy (TI) sequence identification numbers for study species. (PDF 80 kb) Additional file 10: Table S4. References to phylogenies used to hierarchically build the topology of the supertree. (PDF 150 kb)
