# Supplementary material for: Tradeoff between robustness and elaboration in carotenoid networks produces cycles of avian color diversification
Source: Biol Direct. 2015 Aug 20;10:45. doi: 10.1186/s13062-015-0073-6 (PMC4545997; doi:10.1186/s13062-015-0073-6)
Supplement: Additional file 1: Appendix S1. — Confirmed enzymatic reactions in “avian space” of global carotenoid biosynthesis network in bacteria, plants, and animals. References for Appendix S1. (PDF 219 kb) [file 13062_2015_73_MOESM1_ESM.pdf]

Appendix S1. Confirmed enzymatic reactions in "avian space" of global carotenoid biosynthesis network in bacteria, plants, and animals. Only nodes documented in birds (Appendix S2) are included.

			Bacteria					Algae					Plants					Animals				
**CAROTENOID**	**Origin node**	**Reaction**	**NODE**	**PATH**	**ENZ**	**ISO**	**References**	**NODE**	**PATH**	**ENZ**	**ISO**	**References**	**NODE**	**PATH**	**ENZ**	**ISO**	**References**	**NODE**	**PATH**	**ENZ**	**ISO**	**References**
**lutein**	1		?	.	.	.	70	Y	.	.	.	63, 70, 74	Y	.	.	.	63, 70, 74	Y	.	.	.	8, 9, 32, 33, 34, 37, 38, 39, 40, 41, 46, 86, 34, 91
	1	1-67	.	N	N	N	.	.	N	N	N	.	.	N	N	N	.		?	?	Y	46, 34, 93
	1	1-70	.	N	N	N	.	.	N	N	N	.	.	N	N	N	.	.	Y	Y	Y	8, 9, 33, 34, 42, 34, 107, 118
	1	1-52	.	N	N	N	.	.	N	N	N	.	.	?	N	Y	.	.	Y	N	Y	34, 91
	1	1-16	.	N	N	N	.	.	N	N	N	.	.	N	N	N	.	.	Y	Y	N	8, 10, 11, 33, 34, 39, 46, 34, 91, 93
	1	1-5	.	N	N	N	.	.	N	N	N	.	.	N	N	N	.	.	Y	Y	N	37, 41
	1	1-6	.	N	N	N	.	.	Y	Y	N	105	.	N	N	N	.	.	Y	Y	N	32, 38, 39, 40
	1	1-8	.	N	N	N	.	.	N	N	N	.	.	N	N	N	.	.	Y	Y	N	8, 10, 30, 51, 104, 123
	1	1-9	.	N	N	N	.	.	N	N	N	.	.	N	N	N	.	.	?	?	N	8, 30, 104
	1	1-13	.	N	N	N	.	.	N	N	N	.	.	N	N	N	.	.	Y	Y	N	9, 39, 42, 118
	1	1-51	.	N	N	N	.	.	N	N	N	.	.	N	N	N	.	.	Y	Y	n	9, 12, 39, 42, 118
	1	1-45	.	N	N	N	.	.	N	N	N	.	.	N	N	N	.	.	N	N	N	.
	1	1-46	.	Y	Y	N	67	.	N	N	N	.	.	Y	Y	N	67	.	.	.	N	.
**(3R, 3'R) zeaxanthin**	2		Y	.	.	.	2,14, 22, 62	Y	.	.	.	26, 62, 63, 70	Y	.	.	.	62, 44, 63, 74	Y	.	.	.	9,10, 11, 16, 29, 32, 38, 39, 44, 46, 54, 86
	2	2-69	.	N	N	N	.	.	N	N	N	.	.	?	?	N	70	.	Y	Y	N	46
	2	2-70	.	N	N	N	.	.	N	N	N	.	.	N	N	N	.	.	?	Y	Y	9, 33, 34, 42, 34
	2	2-68	.	N	N	N	.	.	N	N	N	.	.	N	N	N	.	.	Y	Y	N	46, 54, 86
	2	2-67	.	N	N	N	.	.	?	?	N	.	.	N	N	N	.	.	?	Y	Y	33, 44, 46, 54
	2	2-20	.	Y	Y	N	97	.	N	N	N	.	.	N	N	N	.	.	Y	Y	N	32, 35, 38, 39, 40, 71, 82
	2	2-16	.	N	N	N	.	.	N	N	N	.	.	N	N	N	.	.	Y	Y	N	9, 10, 32, 42, 46, 34
	2	2-21	.	N	N	N	.	.	Y	Y	N	26, 63	.	Y	Y	N	63	.	Y	Y	N	15
	2	2-31	.	N	N	N	.	.	N	N	N	.	.	N	N	N	.	.	Y	Y	N	9, 12, 42, 119, 120
	2	2-65	.	N	N	N	.	.	N	N	N	.	.	N	N	N	.	.	Y	Y	N	29, 46, 71
	2	2-32	.	Y	Y	N	2, 14	.	Y	Y	N	63	.	N	N	N	.	.	Y	Y	Y	9, 42, 70, 114
	2	2-4	.	N	N	N	.	.	N	N	N	.	.	N	N	N	.	.	Y	Y	N	54, 121
	2	2-18	?	?	?	N	.	.	?	?	N	.	.	N	N	N	.	.	?	?	N	8, 111, 119
**β-carotene**	3		Y	.	.	.	14, 22, 23, 59, 62, 75	Y	.	.	.	60, 61	Y	.	.	.	62, 63, 70	Y	.	.	.	3, 9, 16, 18, 28, 56, 57, 65, 64, 69, 71, 77, 78, 93
	3	3-4	.	Y	Y	N	2, 14, 22, 23, 59, 62, 75	.	Y	Y	N	20, 61, 63	.	Y	Y	N	63, 96		Y	Y	N	57, 65, 69, 93
	3	3-35	.	Y	Y	N	2, 14, 22, 23, 59, 62, 75	.	Y	Y	N	20, 61, 63	.	N	N	N	.	.	Y	Y	N	1, 12, 9, 57, 65, 93
	3	3-41		Y	Y	N	81		Y	Y	N	70, 98	.	Y	Y	N	96		Y	Y	N	5, 16, 18, 28, 64, 77, 78, 83
**β-cryptoxanthin**	4		Y	.	.	.	2,14, 22,23, 59, 62, 75	Y	.	.	.	22, 61, 62, 63, 70	Y	.	.	.	62, 70	Y	.	.	.	9, 10, 65, 69, 56, 57, 71
	4	4-2		Y	Y	N	2,14, 22, 23, 59, 62	.	Y	Y	N	22, 62	.	Y	Y	N	62, 63	.	Y	Y	N	69
	4	4-36	.	Y	Y	N	2,14, 22, 23, 59, 62	.	Y	Y	N	22, 62	.	N	N	N	.	.	Y	Y	N	57, 93
	4	4-30	.	Y	Y	N	97	.	?	?	N	.	.	N	N	N	.	.	Y	Y	N	32, 39, 40
**anhydrolutein**	5		N	.	.	.	.	N	.	.	.	.	N	.	.	.	.	Y	.	.	.	32, 37, 41
**7,8- dihydrolutein**	6		N	.	.	.	.	Y	.	.	.	105	N	.	.	.	.	Y	.	.	.	32, 39, 40, 82
	6	6-7	.	N	N	N	.		?	N	Y	105	.	N	N	Y	.	.	Y	N	Y	32, 39, 40, 82
	6	6-109	.	N	N	N	.	.	N	N	N	.	.	N	N	N	.	.	?	?	N	201
**9-Z-7,8-dihydrolutein**	7		N	.	.	.	.	?	.	.	.	.	N	.	.	.	.	Y	.	.	.	32, 39, 40, 82
	7	7-6	.	N	N	N	.	.	?	N	Y	105	.	N	N	Y	.	.	Y	N	Y	32, 39, 40, 82
**canary xanthophyll A**	8		N	.	.	.	.	N	.	.	.	.	N	.	.	.	.	Y	.	.	.	8, 9, 10, 11, 17, 32, 33, 46, 71, 91, 34, 104
	8	8-10	.	N	N	N	.	.	N	N	N	.	.	N	N	N	.	.	Y	Y	N	9, 11, 32, 33, 39, 34
	8	8-16	.	N	N	N	.	.	N	N	N	.	.	N	N	N	.	.	Y	Y	N	34, 91
	8	8-9	.	N	N	N	.	.	N	N	N	.	.	N	N	N	.	.	Y	Y	N	9, 10, 11, 32, 33, 39, 34, 104, 123
	8	8-110	.	N	N	N	.	.	N	N	N	.	.	N	N	N	.	.	?	?	N	202
**canary xanthophyll B**	9		N	.	.	.	.	N	.	.	.	.	N	.	.	.	74	Y	.	.	.	8, 9, 11, 10, 17, 32, 33, 46, 71, 104
	9	9-17	.	N	N	N	.	.	N	N	N	.	.	N	N	N	.	.	?	?	N	36
	9	9-8	.	N	N	N	.	.	N	N	N	.	.	N	N	N	.	.	Y	Y	N	9, 11
**(3S, 6S,3'S, 6'S) tunaxanthin A**	10		N	.	.	.	.	N	.	.	.	.	?	.	.	.	.	Y	.	.	.	8, 11, 32, 33, 34, 39, 71, 34
	10	10-11	.	N	N	N	.	.	N	N	N	.	.	?	.	.	.		Y	N	Y	8, 11, 39, 71, 108, 111
**(3R, 6R,3'R, 6'R) tunaxanthin F**	11		N	.	.	.	.	N	.	.	.	.	Y	.	.	.	47, 48	Y	.	.	.	8, 91
	11	11-66	.	N	N	N	.	.	N	N	N	.	.	N	N	N	.	.	?	?	N	8
	11	11-65	.	N	N	N	.	.	N	N	N	.	.	N	N	N	.	.	?	?	N	8
**α-doradexanthin**	12		N	.	.	.	.	Y	.	.	.	70	N	.	.	.	.	Y	.	.	.	39, 42, 45, 70
	12	12-14	.	N	N	N	.	.	N	N	N	.	.	N	N	N	.	.	N	N	N	130
	12	12-15	.	N	N	N	.	.	N	N	N	.	.	N	N	N	.	.	Y	Y	N	45, 111
	12	12-32	.	N	N	N	.	.	N	N	N	.	.	N	N	N	.	.	Y	Y	N	71, 117
	12	12-100	.	N	N	N	.	.	N	N	N	.	.	N	N	N	.	.	?	?	N	200
**(3S,4R,3'R,6'R) 4-hydroxylutein**	13		N	.	.	.	.	N	.	.	.	.	N	.	.	.	.	Y	.	.	.	9, 39, 42
	13	13-51	.	N	N	N	.	.	N	N	N	.	.	N	N	N	.	.	Y	N	N	42, 111
	13	13-14	.	N	N	N	.	.	N	N	N	.	.	N	N	N	.	.	Y	?	N	9, 12, 39, 42, 111, 118, 130
**fritschiellaxanthin**	14		N	.	.	.	.	Y	.	.	.	70	N	.	.	.	.	Y	.	.	.	9, 39, 42, 70
	14	14-12	.	N	N	N	.	.	?	N	?	.	.	?	N	Y	.	.	N	N	N	.
	14	14-15	.	N	N	N	.	.	N	N	N	.	.	N	N	N	.	.	Y	?	N	9, 111
**papilioerythrinone**	15		N	.	.	.	.	Y	.	.	.	.	N	.	.	.	.	Y	.	.	.	45, 71
	15	15-108	.	N	N	N	.	.	N	N	N	.	.	N	N	N	.	.	?	?	N	201
**3'-dehydrolutein**	16		N	.	.	.	.	?	.	.	.	.	?	.	.	.	.	Y	.	.	.	8, 9, 10, 11, 33, 34, 35, 46, 71, 34, 91, 93
	16	16-1	.	N	N	N	.	.	?	.	.	.	.	N	N	N	.	.	Y	Y	N	9, 11, 34, 91
	16	16-8	.	N	N	N	.	.	N	N	N	.	.	N	N	N	74		N	Y	N	10, 11, 34, 92

	16	16-9	.	N	N	N	.	.	N	N	N	.	.	N	N	N	.	.	Y	Y	N	10, 11, 34
	16	16-70	.	N	N	N	.	.	N	N	N	.	.	N	N	N	.	.	Y	Y	N	33, 34, 91
	16	16-109	.	N	N	N	.	.	N	N	N	.	.	N	N	N	.	.	?	?	N	201
**piprixanthin**	17		N	.	.	.	.	N	.	.	.	.	N	.	.	.	.	Y	.	.	.	36
	17	17-71		N	N	N	.	.	N	N	N	.	.	N	N	N		.	?	?	N	36
	17	17-18		Y	N	N	.	.	?	N	N	.	.	N	N	N	.		Y	?	N	36
**rhodoxanthin**	18		Y	.	.	.	70	Y	.	.	.	.	Y	.	.	.	70	Y	.	.	.	36
**7,8,7',8'-tetrahydrozeaxanthin**	19		N	.	.	.	.	N	.	.	.	.	N	.	.	.	.	Y	.	.	.	32, 35, 40, 82
**7,8-dihydrozeaxanthin**	20		Y	Y	.	.	59, 97	N	.	.	.	.	N	.	.	.	.	Y	.	.	.	32, 38, 39, 40, 71
	20	20-19	.	N	N	N	.	.	N	N	N	.	.	N	N	N	.	.	Y	Y	N	32, 35, 40, 82
**antheraxanthin**	21		N	.	.	.	.	Y	.	.	.	26, 63, 70	Y	.	.	.	62, 63, 70, 74	Y	.	.	.	9, 71
	21	21-22	.	N	N	N	.	.	Y	Y	N	63	.	Y	Y	N	63, 74	.	N	N	N	.
	21	21-2	.	N	N	N	.	.	Y	Y	N	63	.	Y	Y	N	63, 74, 75	.	N	N	N	.
	21	21-76	.	N	N	N	.	.	?	?	N	.	.	Y	Y	N	71, 115	.	N	N	N	.
**violaxanthin**	22		N	N	.	N	.	Y	.	.	.	26, 63, 70	Y	.	.	.	62, 63, 70, 74	Y	.	.	.	71
	22	22-23	.	N	N	N	.	.	Y	Y	N	26, 63, 70	.	Y	Y	N	62, 63, 70, 74		N	N	.	.
	22	22-21	.	N	N	N	.	.	Y	Y	N	26, 63, 70	.	Y	Y	N	62, 63, 70, 74		N	N	.	.
**neoxanthin**	23		N	.	.	.	.	Y	.	.	.	26, 63	Y	.	.	.	62, 63, 70, 74, 105	Y	.	.	.	49, 71
	23	23-24	.	N	N	N	.	.	N	N	N	.	.	?	?	N	.	.	Y	Y	N	49
**neochrome**	24		N	.	.	.	.	Y	.	.	.	105, 112	Y	.	.	.	70	Y	.	.	.	49
**idoxanthin**	25		N	.	.	.	.	?	.	.	.	111	N	.	.	.	.	Y	.	.	.	9,11, 13, 15, 16, 24, 28, 71
	25	25-34	.	N	N	N	.	.	N	N	N	.	.	N	N	N	.	.	Y	?	N	120, 126, 127
	25	25-32	.	N	N	N	.	.	N	N	N	.	.	.	N	N	.	.	Y	Y	N	11, 13, 15, 16, 35
	25	25-74	.	N	N	N	.	.	N	N	N	.	.	N	N	N	.	.	Y	Y	N	11, 71
**fucoxanthin**	26		N	.	.	.	.	Y	.	.	.	70	N	.	.	.	.	Y	.	.	.	5, 51, 88, 71
	26	26-27	.	N	.	N	.	.	N	N	.	.	.	N	N	N	.		Y	Y	N	5, 51, 88, 122
**fucoxanthinol**	27		N	.	.	.	.	Y	.	.	.	70, 124	N	N	N	N	.	Y	.	.	.	5, 51, 88, 71
	27	27-28	.	N	N	N	.	.	N	N	N	.	.	N	N	N	.	.	Y	Y	N	5, 51
	27	27-29	.	N	N	N	.	.	N			.	.	N	N	N	.	.	?	?	N	122
**amarouciaxanthin**	28		N	.	.	.	.	N	.	.	.	.	N	.	.	.	.	Y	.	.	.	51
	28	28-29	.	N	N	N	.	.	N	N	N	.	.	N	N	N	.		Y	Y	N	5, 51, 88
**paracentrone**	29		N	.	.	.	.	?	.	.	.	112	N	.	.	.	.	Y	.	.	.	5, 88
**7,8 dihydro β-cryptoxanthin**	30		Y	.	.	.	97	?	.	.	.	97	?	.	.	.	97	Y	.			32, 39, 40
**4-hydroxyzeaxanthin**	31		N	.	.	.	.	N	.	.	.	.	N	N	N	N	.	Y	.	.	.	8, 9, 16, 11, 71
	31	31-32	.	N	N	N	.	.	N	N	N	.	.	N	N	N	.	.	Y	?	N	9, 12, 42
	31	31-2	.	N	N	N	.	.	N	N	N	.	.	N	N	N	.	.	Y	Y	N	8, 9, 11
	31	31-74	.	N	N	N	.	.	N	N	N	.	.	N	N	N	.	.	Y	Y	N	42, 120
**adonixanthin**	32		Y	.	.	.	2, 14, 22, 59	Y	.	.	.	20, 61, 63	Y	.	.	.	80, 96, 125	Y	.	.	.	9, 11, 15, 16, 28, 29, 32, 35, 42
	32	32-34	.	Y	Y	N	2, 14, 22, 59	.	Y	Y	N	20, 61, 63	.	N	N	N	.	.	Y	Y	N	9, 12, 35, 104, 118
	32	32-2	.	N	N	N	.	.	N	N	N	.	.	N	N	N	.	.	Y	Y	N	9, 16, 35, 39
	32	32-25	.	N	N	N	.	.	N	N	N	.	.	N	N	N	.	.	Y	Y	N	9, 42, 70
	32	32-31	.	N	N	N	.	.	N	N	N	.	.	N	N	N	.	.	Y	Y	N	8, 9, 11, 16
	32	32-102	.	N	N	N	.	.	N	N	N	.	.	N	N	N	.	.	?	?	N	200
**13 cis-(3R, 3'R) astaxanthin**	33		Y	.	.	.	73	Y	.	.	.	84	?	.	.	.	.	Y	.	.	.	15, 71, 85
	33	33-34	.	Y	N	Y	73	.	Y	N	Y	84	.	?	N	Y	.	.	Y	N	Y	15, 27, 71, 85
**(3'R, 3R) astaxanthin**	34		Y	.	.	.	2, 14, 22, 59, 102	Y	.	.	.	20, 63, 70	Y	.	.	.	74, 96	Y	.	.	.	8, 11, 13, 15, 16, 27, 29, 35, 45, 57, 71
	34	34-32	.	N	N	N	.	.	N	N	N	.	.	N	N	N	.	.	Y	Y	N	8, 9, 11, 15, 29
	34	34-25	.	N	N	N	.	N	N	N	N	.	.	N	N	N	.	.	Y	Y	N	8, 11, 13, 15, 27
	34	34-75	.	Y	N	Y	73	.	Y	N	Y	84	.	?	N	Y	.	.	Y	N	Y	15, 71, 85
	34	34-38	.	N	N	N	.	.	N	N	N	.	.	N	N	N	.	.	Y	Y	N	121
	34	34-33	.	Y	Y	Y	73	.	Y	N	Y	84	.	?	N	Y	.	.	Y	N	Y	15, 27, 71, 85
	34	34-103	.	N	N	N	.	.	N	N	N	.	.	N	N	N	.	.	?	?	N	200
**echinenone**	35		Y	.	.	.	2, 14, 22, 23, 25, 59, 62, 63, 75, 100	Y	.	.	.	61, 63, 70	Y	.	.	.	80, 96	Y	.	.	.	15, 16, 28, 64, 71, 77, 78, 83, 93
	35	35-3	.	N	N	N	.	.	N	N	N	.	.	N	N	N	.	.	Y	Y	N	4, 15, 16
	35	35-39	.	Y	Y	N	81, 100	.	Y	Y	N	.	.	N	N	N	.	.	Y	Y	N	6, 28, 64, 77, 78
	35	35-37	.	Y	Y	N	2, 14, 22, 59, 62, 102	.	Y	Y	N	62, 63	.	N	N	N	.	.	Y	Y	N	2, 9, 57, 64, 93
	35	35-36	.	Y	Y	N	2, 14, 22, 23, 59, 62, 63, 102	.	Y	Y	N	62, 63	.	Y	Y	N	80, 96, 125	.	Y	Y	N	57, 71, 101, 93
	35	35-41	.	N	N	N	.	.	N	N	N	.	.	N	N	N	.	.	Y	Y	N	16
**3'-hydroxyechinenone**	36		Y	.	.	.	2, 14, 22, 59, 63, 102	Y	.	.	.	20, 63, 70	Y	.	.	.	80, 96	Y	.	.	.	15, 17, 57, 71, 93
	36	36-32	.	Y	Y	N	2, 14, 22, 59, 102	.	Y	Y	N	20, 63	.	Y	Y	.	80, 96, 125	.	Y	?	N	57, 71, 93
	36	36-38	.	Y	Y	N	2,14, 59, 102	.	Y	Y	N	20, 63	.	N	N	N	.	.	Y	Y	N	57, 71, 93
	36	36-4	.	N	N	N	.	.	N	N	N	.	.	N	N	N	.	.	Y	Y	N	9, 15
**canthaxanthin**	37		Y	.	.	.	2,14,22, 25, 59, 63, 81	Y	.	.	.	61, 63, 70	Y	.	.	.	.	Y	.	.	.	3, 15, 16, 28, 57, 64, 77, 78, 93
	37	37-35	.	N	N	N	.	.	N	N	N	.	.	N	N	N	.	.	Y	Y	N	4, 8, 15, 121
	37	37-38	.	Y	Y	N	2, 14, 22, 59, 63, 102	.	Y	Y	N	62, 63	.	Y	Y	N	80, 96	.	Y	Y	N	9, 17, 57, 93, 101, 121
	37	37-40	.	N	N	N	.	.	N	N	N	.	.	N	N	N	.	.	Y	Y	N	8, 16
	37	37-39	.	N	N	N	.	.	N	N	N	.	.	N	N	N	.	.	Y	Y	N	15, 16, 31, 82
**adonirubin**	38		Y	.	.	.	2,14, 22, 59, 102	Y	.	.	.	20, 63, 70	Y	.	.	.	96	Y	.	.	.	1, 3, 6, 7, 17, 57, 71, 101
	38	38-34	.	Y	Y	N	2, 14, 22, 59, 102	.	Y	Y	N	63	.	Y	Y	N	74, 80, 96, 125	.	Y	Y	N	9, 17, 57, 93, 101, 71, 77, 103
	38	38-36	.	N	N	N	.	.	N	N	N	.	.	N	N	N	.	.	Y	Y	N	15
	38	38-37	.	Y	Y	N	89	.	Y	Y	N	62, 63	.	Y	Y	N	80, 96	.	Y	Y	N	89, 75
	38	38-107	.	N	N	N	.	.	N	N	N	.	.	N	N	N	.	.	?	?	N	200
**4-hydroxy-echinenone**	39		Y	.	.	.	81, 100	Y	.	.	.	70	N	.	.	.	.	Y	.	.	.	6, 16, 30, 31, 71, 77, 78, 82, 83, 99
	39	39-40	.	N	N	N	.	.	N	N	N	.	.	N	N	N	.	.	Y	Y	N	15, 16, 31
	39	39-35	.	N	N	N	.	.	N	N	N	.	.	N	N	N	.	.	Y	Y	N	16

	39	39-37	.	Y	?	N	81, 100	.	?	?	N	.	.	N	N	N	.	.	Y	Y	N	6, 28, 35, 64, 77, 78, 83
**isozeaxanthin**	40		Y	.	.	.	81	Y	.	.	.	70, 98	Y	.	.	.	80	Y	.	.	.	8, 16, 28, 31, 53, 71, 80, 81
	40	40-41	.	N	N	N	.	.	N	N	.	.	.	N	N	N	.	.	Y	Y	N	8, 15, 16
	40	40-60	.	N	N	N	.	.	N	N	.	.	.	Y	Y	N	80	.	N	N	N	.
	40	40-37	.	Y	Y	N	81	.	Y	Y	N	70, 98	.	N	N	N	.	.	Y	Y	N	28, 64
	40	40-39	.	Y	Y	N	81	.	?	?	N	70	.	N	N	N	.	.	N	N	N	.
**β-isocryptoxanthin**	41		Y	.	.	.	81	Y	.	.	.	79, 98	Y	.	.	.	70	Y	.	.	.	5, 9, 18, 16, 31, 71, 77, 78, 83
	41	41-3	.	N	N	N	.	.	N	.	N	.	.	N	N	N	.		N	Y	N	16
	41	41-40	.	Y	Y	N	81		Y	Y	N	70, 98	.	Y	Y	N	96	.	Y	Y	N	28, 71
	41	41-35	.	?	Y	N	81	.	?	?	N	70, 98	.	N	N	N	.	.	Y	Y	N	18, 28, 64, 77, 78, 83
**α-carotene**	42		Y	N	.	.	62, 63, 70, 76	Y	.	.	.	62, 63, 70, 74	Y	.	.	.	58, 59, 63, 74, 76	Y	.	.	.	52, 53, 71
	42	42-46	.	N	N	N	.	.	N	N	N	.	.	Y	Y	N	131	.	?	?	N	.
	42	42-45	.	?	Y	N	59	.	Y	Y	N	63	.	Y	Y	N	63	.	N	N	.	.
	42	42-43	.	N	N	N	.	.	N	N	N	.	.	?	?	N	96, 125	.	Y	?	.	72
**α-isocryptoxanthin**	43		?	.	.	.	.	?	.	.	.	.	?	.	.	.	.	Y	.	.	.	52, 71, 72
	43	43-44	.	N	N	N	.	.	N	N	N	.	.	N	N	N	.	.	Y	Y	N	52, 72
**phoenicopterone**	44		N	.	.	.	.	Y	.	.	.	70, 113	N	.	.	.	.	Y	.	.	.	53, 71, 72
**zeinoxanthin**	45		N	.	.	.	.	Y	.	.	.	58, 59, 63	Y	.	.	.	58, 59, 63	N	.	.	.	.
	45	45-1	.	N	N	N	.	.	Y	Y	N	63	.	Y	Y	N	63	.	N	N	N	.
	45	45-42	.	N	N	N	.	.	N	N	N	.	.	N	N	N	.	.	N	N	N	.
**α-cryptoxanthin**	46		N	.	.	.	.	Y	.	.	.	63, 70	Y	.	.	.	63, 70	Y	.	.	.	46, 71, 106
	46	46-1	.	Y	Y	N	67	.	N	N	N	.	.	Y	Y	N	67	.	.	.	N	.
	46	46-42	.	N	N	N	.	.	N	N	N	.	.	Y	Y	N	131	.	?	?	N	8
**rubixanthin**	47		Y	.	.	.	70, 94	Y	.	.	.	70, 95	Y	.	.	.	70	Y	.	.	.	55, 57, 93, 71
	47	47-48	.	Y	Y	N	.	.	?	Y	N	.	.	?	?	N	.	.	Y	Y	N	57
	47	47-49	.	?	?	Y	.	.	?	Y	Y	.	.	Y	N	Y	55	.	N	N	N	55, 104, 128
	47	47-2	.	Y	?	N	129	.	.	.	.	.	.	.	.	.	.	.	.	.	.	.
	47	47-4	.	Y	?	N	129	.	.	.	.	.	.	.	.	.	.	.	.	.	.	.
**4-oxo-rubixanthin**	48		?	.	.	.	.	?	.	.	.	.	?	.	.	.	.	Y	.	.	.	57, 93
	48	48-50	.	?	?	?	.	.	?	?	?	.	.	N	N	N	.	.	N	N	N	.
**gazaniaxanthin**	49		?	.	.	.	.	?	.	.	.	.	Y	.	.	.	70	Y	.	.	.	71, 104
	49	49-50	.	?	?	N	.	.	?	?	N	.	.	?	?	.	.	.	Y	Y	N	93, 104
	49	49-47	.	?	?	Y	.	.	?	?	Y	.		Y	Y	Y	55	.	N	N	N	55, 104, 128
**4-oxo-gazaniaxanthin**	50		?	.	.	.	.	?	.	.	.	.	?	.	.	.	.	Y	.	.	.	57, 93, 104
	50	50-48	.	?	?	?	.		?	?	?	.	.	N	N	N	.	.	?	?	Y	93
**(3S,4R,3'S,6'R) 4-hydroxylutein**	51		N	.	.	.	.	N	.	.	.	.	N	.	.	.	.	Y	.	.	.	9, 39, 42
	51	51-13	.	N	N	N	.	.	N	N	N	.	.	N	N	N	.	.	Y	N	N	42, 70, 111
	51	51-12	.	N	N	N	.	.	N	N	N	.	.	N	N	N	.	.	Y	Y	N	9, 12, 39, 42, 118
**cis lutein**	52		N	.	.	.	.	?	.	.	.	.	?	.	.	.	.	Y	.	.	.	34, 91
	52	52-1	.	N	N	N	.	.	?	N	N	.	.	?	N	Y	.	.	Y	N	Y	34, 91, 93
**3,4, 3',4'-tetradehydroisozeanthin**	60		N	.	.	.	.	N	.	.	.	.	Y	.	.	.	80	N	.	.	.	.
	60	60-37	.	N	N	N	.	.	N	N	N	.	.	Y	N	N	80	.	N	N	N	.
**γ-carotene**	61		Y	.	.	.	62, 73, 94	Y	.	.	.	63	Y	.	.	.	58, 62, 63, 70	Y	.	.	.	71, 72
	61	61-3	.	Y	Y	N	62, 73	.	Y	Y		63		Y	Y	N	62, 63, 74		N	N	N	.
	61	61-47		Y	Y	N	73, 94		?	?	N	.	.	?	Y	N	58, 70		?	?	N	.
	61	61-42	.	N	N	N	62	.	Y	Y	.	63	.	Y	Y	N	62, 63, 70	.	N	N	N	.
**δ-carotene**	64		Y	.	.	.	62	Y	.	.	.	63, 70	Y	.	.	.	63, 74	Y	.	.	.	71
	64	64-65	.	N	N	N	63, 76	.	Y	Y	N	62, 63	.	Y	Y	N	62, 63, 87		N	N	N	.
	64	64-42	.	Y	Y	N	62	.	Y	Y	N	62, 63	.	Y	Y	N	62, 63, 87		N	N	N	.
**(6S, 6'S) ε,ε- carotene**	65		N	.	.	.	.	Y	.	.	.	63, 70	Y	.	.	.	48, 63, 74, 87	Y	.	.	.	8, 39, 46, 71
	65	65-66	.	N	N	N	.	.	?	?	N	.	.	Y	Y	N	47	.	?	?	N	.
**ε-carotene 3 diol**	66		N	.	.	.	.	?	.	.	.	.	Y	.	.	.	47	Y	.	.	.	.
	66	66-65	.	N	N	N	.	.	N	.	N	.	.	N	N	N	.	.	?	?	N	8
	66	66-11	.	N	N	N	.	.	N	N	N	.	.	Y	Y	N	47	.	?	?	N	.
**(3R, 3'S) meso-zeaxanthin**	67		N	.	.	.	.	N	.	.	.	.	N	.	.	.	44	Y	.	.	.	16, 33, 44, 46, 54, 86, 34, 93
	67	67-2	.	N	N	N	.	.	N	N	N	.	.	N	N	N	.	.	?	?	Y	33, 44, 54
	67	67-1	N	N	N	N	.	.	N	N	N	.	.	N	N	N	.	.	Y	Y	Y	34, 93
**galloxanthin**	68		N	.	.	.	.	N	.	.	.	.	N	.	.	.	.	Y	.	.	.	29, 46, 54, 71
**β-apo-2'-carotenol**	69		?	Y	.	.	111	?	.	.	.	111	N	.	.	.	111	Y	.	.	.	46
**3'-epilutein**	70		N	.	.	.	.	?	.	.	.	112	?	.	.	.	.	Y	.	.	.	9, 11, 33, 34, 39, 71, 34
	70	70-16	.	N	N	N	.	.	N	N	N	.	.	N	N	N	.	.	?	?	N	33, 46, 34
	70	70-8	.	N	N	N	.	.	N	N	N	.	.	N	N	N	.	.	Y	Y	N	33
	70	70-1	.	N	N	.	.	.	?	?	N	.	.	N	N	N	.	.	?	?	Y	33, 34, 71, 34
	70	70-2	.	N	N	N	.	.	N	N	N	.	.	N	N	N	.	.	Y	Y	Y	33, 34, 34, 91
	70	70-12	.	N	N	N	.	.	N	N	N	.	.	N	N	N	.	.	?	?	N	93
	70	70-51	.	N	N	N	.	.	N	N	N	.	.	N	N	N	.	.	Y	Y	N	9, 12, 39, 42, 118
**resonance stabilized form**	71		?	.	.	.	.	?	.	.	.	.	N	.	.	.	.	Y	.	.	.	36
	71	71-18	.	?	N	N	.	.	?	N	N	.	.	N	N	N	.	.	?	?	N	36
**β-carotene -3,4, 3', 4'- tetrol**	74		N	.	.	.	.	Y	.	.	.	70	N	.	.	.	.	Y	.	.	.	11, 42, 71
	74	74-31	.	N	N	N	.	.	N	N	N	.	.	N	N	N	.	.	Y	Y	N	11
	74	74-25	.	N	N	N	.	.	N	N	N	.	.	N	N	N	.	.	Y	Y	N	11, 42, 120
**9-cis - (3S, 3'S) astaxanthin**	75		?	.	.	.	.	Y	.	.	.	84	?	.	.	.	.	Y	.	.	.	15, 71, 85
	75	75-34	.	Y	N	Y	73	.	Y	N	Y	84	.	?	N	Y	.	.	Y	N	Y	15, 27, 71, 85

**eschscholtzxanthin**	76		N	.	.	.	.	N	.	.	.	.	Y	.			70	Y	.	.	.	.
	76	76-77	.	N	N	N	.	.	N	N	N	.	.	Y	Y	N	111, 116	.	N	N	N	.
**eschscholtzxanthone**	77		N	.	.	.	.	N	.	.	.	.	Y	.	.	.	116	N	.	.	.	.
	77	77-18	.	N	N	N	.	.	N	N	N	.	.	Y	Y	N	116	.	N	N	N	.
**xipholenin (Note 1)**	100		N	.	.	.	.	N	.	.	.	.	N	.	.	.	.	Y	.	.	.	200, 203
	100	100-101	.	N	N	N	.	.	N	N	N	.	.	N	N	N	.	.	?	?	N	200, 203
**2,3-didehydro-xipholenin (Note 2)**	101		N	.	.	.	.	N	.	.	.	.	N	.	.	.	.	Y	.	.	.	200, 203
**rupicolin (Note 3)**	102		N	.	.	.	.	N	.	.	.	.	N	.	.	.	.	Y	.	.	.	200
**3'-hydroxy-3-methoxy-canthaxanthin**	103		N	.	.	.	.	N	.	.	.	.	N	.	.	.	.	?	.	.	.	200
	103	103-104	.	N	N	N	.	.	N	N	N	.	.	N	N	N	.	.	?	?	N	200
**pompadourin (Note 4)**	104		N	.	.	.	.	N	.	.	.	.	N	.	.	.	.	Y	.	.	.	200, 203
	104	104-105	.	N	N	N	.	.	N	N	N	.	.	N	N	N	.	.	?	?	N	200, 203
**2,3-Didehydro-pompadourin (Note 5)**	105		N	.	.	.	.	N	.	.	.	.	N	.	.	.	.	Y	.	.	.	200, 203
	105	105-106	.	N	N	N	.	.	N	N	N	.	.	N	N	N	.	.	?	?	N	200, 203
**cotingin (Note 6)**	106		N	.	.	.	.	N	.	.	.	.	N	.	.	.	.	Y	.	.	.	200, 203
**brittonxanthin (Note 7)**	107		N	.	.	.	.	N	.	.	.	.	N	.	.	.	.	Y	.	.	.	200, 203
**cymbirhynchin (Note 8)**	108		N	.	.	.	.	N	.	.	.	.	N	.	.	.	.	Y	.	.	.	201
**eurylaimin (Note 9)**	109		N	.	.	.	.	N	.	.	.	.	N	.	.	.	.	Y	.	.	.	201
**4-hydroxy-canary xanthophyll A**	110		N	.	.	.	.	N	.	.	.	.	N	.	.	.	.	Y	.	.	.	202

Y: confirmed present, N: confirmed absent, ?: expected, but no experimental evidence

NODE -- carotenoid compound (number refers to network)

PATH -- reaction from origin node to derived node

ENZ -- presence of enzyme

ISO -- evidence of isomerization

Notes:

1: 3-Methoxy-3'-hydroxy-β,ε-carotene-4-one

2: 3'-hydroxy-3-methoxy-2,3-didehydro-β,β-carotene-4-one

3: 3'-hydroxy-3-methoxy-β,β-carotene-4-one

4: 3,3'-Dimethoxy-β,β-carotene-4,4'-dione or 3,3'-dimethoxy-canthaxanthin

5: 3,3'-Dimethoxy-2,3-didehydro-β,β-carotene-4,4'dione

6: 3,3'-Dimethoxy-2,3,2',3'-tetradehydro-β,β-carotene-4,4'dione

7: 3-methoxy-β,β-carotene-4,4'dione or 3-methoxy-canthaxanthin

8: 2,3-didehydro-papilioerythrinone

9: 7,8-dihydro-3'-dehydro-lutein

Literature Sources for Appendix S1:

1. Davies, B. H., W. J. Hsu, and C. O. Chichester. 1970. The mechanism of the conversion of beta-carotene into canthaxanthin by the brine shrimp, Artemia salina L. (Crustacea: Branchiopoda). Comp. Biochem Physiol. 33: 601-615.

2. Fraser, P. D., S. Hiroshi, and M. Norihiko. 1998. Enzymic confirmation of reactions involved in routes to astaxanthin formation, elucidated using a direct substrate *in vitro* assay. Eur. J. Biochem. 252: 229-236.

3. Fox, D. L., A. A. Wolfson, and J. W. McBeth. 1969. Metabolism of □-carotene in the american flamingo, Phoenicopterus rubber. Comp. Biochem. Physiol. 29:1223-1229.

4. Guillou, A., G. Choubert, T. Storebakken, J. De La Noüe, and S. Kaushik. 1989. Bioconversion pathway of astaxanthin into retinol$_2$ in mature rainbow trout (Salmo gairdneri Rich.). Comp. Biochem. Physiol. 94: 484-485.

5. Hallenstvet, M., E. Pyberg, and S. Liaaen-Jensen. 1978. Animal carotenoids - XIV Carotenoids of Psammechinus miliaris (sea-urchin). Comp. Biochem. Physiol. 60: 173-175.

6. Hata, M., and Hata, M. 1969. Carotenoid metabolism in Artemia salina L. Comp. Biochem. Physiol. 29: 985-994.

7. Herring, P. J. 1968. The carotenoid pigments of Daphnia magna Straus. II. Aspects of pigmentary metabolism. Comp. Biochem. Physiol. 24: 205-221.

8. Katsuyama, M., and Matsuno,T. 1988. Carotenoid and vitamin A, and metabolism of carotenoids, □-carotene, canthaxanthin, astaxanthin, zeaxanthin, lutein and tunaxanthin in tilapia *Tilapia nilotica*. Comp. Biochem. Physiol. B 90: 131-139.

9. Matsuno,T. 1991. Xanthophylls as precursors of retinoids. Pure Appl. Chem. 63: 81-88.

10. Matsuno, T., T. Hirono, Y. Ikuno, T. Maoka, M. Shimizu, and T. Komori. 1986. Isolation of three new carotenoids and proposed metabolic pathways of carotenoids in hen's egg yolk. Comp. Biochem. Physiol. 84: 477-481.

11. Matsuno, T., M. Katsuyama, T. Maoka, T. Hirono, and T. Komori. 1985. Reductive metabolic pathways of carotenoids in fish (3S, 3'S)-astaxanthin to tunaxanthin A, B and C. Comp. Biochem. Physiol. 80:779-789.

12. Matsuno,T., H. Matsutaka, and S. Nagata. 1981. Metabolism of lutein and zeaxanthin to ketocarotenoids in goldfish, Carassius auratus. Bull. Jap. Soc. Sci. Fish. 47: 605-611.

13. Miki,W., K. Yamaguchi, S. Konosu, and T. Watanabe. 1984. Metabolism of dietary carotenoids in eggs of red sea bream. Comp. Biochem. Physiol. 77: 665 -668.

14. Misawa, N., Y. Satomi, K. Kondo, A. Yokoyama, S. Kajiwara, T. Saito, T. Ohtani, and W. Miki. 1995. Structure and functional analysis of a marine bacterial carotenoid biosynthesis gene cluster and astaxanthin biosynthetic pathway proposed at the gene level. J Bacteriol. 177: 6575-6584.

15. Schiedt, K., F. J. Leuenberger, M. Vecchi, and E. Glinz. 1985. Absorption, retention and metabolic transformations of carotenoids in rainbow trout, salmon and chicken. Pure Appl. Chem. 57: 685-692.

16. Schiedt, K., M. Vecchi, E. Glinz, and T. Storebakken. 1988. Metabolism of carotenoids in salmonids: metabolism of astaxanthin and canthaxanthin in the skin of atlantic salmon (*Salmo salar, L.*). Helv. Chim. Acta 71:887-896.

17. Stradi, R., G. Celentano, E. Rossi, G. Rovati, and M. Pastore. 1995. Carotenoids in bird plumage: I. The carotenoid pattern in a series of Palearctic Carduelinae. Comp. Biochem. Physiol. 110:131 -143.

18. Tsushima, M., T. Kawakami, and T. Matsuno. 1993. Metabolism of carotenoids in sea-urchin *Pseudocentrotus depressus*. Comp. Biochem. Physiol. 106: 737 -741.

19. Wyss, A., G. Wirtz, W. Woggon, R. Brugger, M. Wyss, A. Friedlein, H. Bachmann, and W. Hunziker. 2000. Cloning and expression of beta,beta-carotene 15,15'-dioxygenase. Biochem Biophys Res Commun. 271:334-336.

20. Liu, B.-H. and Y.-K. Lee 1999. Composition and biosynthetic pathways of carotenoids in the astaxanthin-producing green alga *Chlorococcum sp.* Biotechnology Letters 21(11): 1007-1010.

21. Fraser, P. D. and P. M. Bramley (2004). The biosynthesis and nutritional uses of carotenoids. Progress in Lipid Research 43(3): 228-265.

22. Martin, J., E. Gudina, et al. 2008. Conversion of beta-carotene into astaxanthin: two separate enzymes or a bifunctional hydroxylase-ketolase protein? Microbial Cell Factories 7(1): 3.

23. Punginelli, C., A. Wilson, et al. 2009. Influence of zeaxanthin and echinenone binding on the activity of the orange carotenoid protein. Biochimica et Biophysica Acta - Bioenergetics 1787(4): 280-288.

24. Aas, G. H., B. Bjerkeng, et al. 1997. Idoxanthin, a major carotenoid in the flesh of Arctic charr (*Salvelinus alpinus*) fed diets containing astaxanthin. Aquaculture 150(1-2): 135-142.

25. Schwartzel E.M. and J.J Cooney. 1970 Isolation and identification of echinenone from *Micrococcus roseus*. J Bacteriology. 104: 272-274.

26. Esteban, R., B. Martínez, et al. 2009. Carotenoid composition in Rhodophyta: insights into xanthophyll regulation in *Corallina elongate*. European Journal of Phycology 44(2): 221 - 230.

27. Bjerkeng B., Hatlen B. and M. Jobling 2000 Astaxanthin and its metabolites idoxanthin and crustaxanthinin flesh, skin, and gonads of sexually immature and maturingArctic charr (*Salvelinus alpinus* (L.)). Comp Biochem Physiol 99(3): 395-404.

28. Maoka, T. and T. Matsuno 1989. Metabolism of carotenoids in terrestrial snail Euhadra Callizona amaliae. Comparative Biochemistry and Physiology Part B: Comparative Biochemistry 92(1): 41-43.

29. Schiedt, K., Bischof, S. and Glinz, E. 1991. Recent progress on carotenoid metabolism in animals. Pure Appl. Chem. 63: 89-100

30. Matsuno, T., K. Katagiri, et al. 1985. Novel reductive metabolic pathways of 4-oxo-[beta]-end group in carotenoids of the spindle shell *Fusinus perplexus*. Comparative Biochemistry and Physiology Part B: Comparative Biochemistry 81(4): 905-908.

31. Henmi, H., M. Hata, et al. 1991. Studies on the carotenoids in the muscle of salmon--V. Combination of astaxanthin and canthaxanthin with bovine serum albumin and egg albumin. Comparative Biochemistry and Physiology Part B: Comparative Biochemistry 99(3): 609-612.

32. Stradi, R., J. Hudon, et al. 1998. Carotenoids in bird plumage: the complement of yellow and red pigments in true woodpeckers (Picinae). Comparative Biochemistry and Physiology Part B: Biochemistry and Molecular Biology 120(2): 223-230.

33. Krinsky N.I., Landrum J.T. and R.A. Bone. 2003. Biologic mechanisms of the protective role of lutein and zeaxanthin in the eye. Annu. Rev. Nutr. 23:171–201

34. Khachik F. 2006. Distribution and metabolism of dietary carotenoids in humans as a criterion for development of nutritional supplements. Pure Appl. Chem. 78(8): 1551–1557.

35. Goodwin, T. W. 1986. Metabolism, Nutrition, and Function of Carotenoids. Annual Review of Nutrition 6(1): 273-297.

36. Hudon J., Anciães M., Bertacche V. and R. Stradi 2007. Plumage carotenoids of the Pin-tailed Manakin (*Ilicura militaris*): evidence for the endogenous production of rhodoxanthin from a colour variant. Comp Biochem and Physiol Part B 147: 402–411.

37. McGraw, K. J., E. Adkins-Regan, Parker R.S. 2002. Anhydrolutein in the zebra finch: a new, metabolically derived carotenoid in birds. Comparative Biochemistry and Physiology Part B: Biochemistry and Molecular Biology 132(4): 811-818.

38. McGraw K.J., Hill G.E., Stradi R., Parker R.S. 2002. The effect of dietary carotenoid access on sexual dichromatism and plumage pigment composition in the American goldfinch. Comparative Biochemistry and Physiology *B* 131: 261–269.

39. Matsuno, T. 2001. Aquatic animal carotenoids. Fisheries Science 67(5): 771-783.

40. Tsushima, M., Y. Ikuno, Nagata S., Kodama K. and T. Matsuno. 2002 Comparative biochemical studies of carotenoids in catfishes. Comparative Biochemistry and Physiology Part B: Biochemistry and Molecular Biology 133(3): 331-336.

41. McGraw, K. J. and M.C. Nogare 2004. Carotenoid pigments and the selectivity of psittacofulvin-based coloration systems in parrots. Comp Biochem Physiol Part B: Biochemistry and Molecular Biology 138(3): 229-233.

42. Ohkubo, M., M. Tsushima, et al. 1999. Carotenoids and their metabolism in the goldfish *Carassius auratus* (Hibuna). Comparative Biochemistry and Physiology Part B: Biochemistry and Molecular Biology 124(3): 333-340.

43. Andersson, Staffan, et al. 2007. Carotenoid content and reflectance of yellow and red nuptial plumages in widowbirds (*Euplectes* spp.). Functional Ecology 21:272-281.

44. Maoka, T., A. Arai, et al. 1986. The first isolation of enantiomeric and meso-zeaxanthin in nature. Comparative Biochemistry and Physiology Part B: Comparative Biochemistry 83(1): 121-124.

45. Stradi, R., E. Pini, et al. 2001. Carotenoids in bird plumage: the complement of red pigments in the plumage of wild and captive bullfinch (*Pyrrhula pyrrhula*). Comparative Biochemistry and Physiology Part B: Biochemistry and Molecular Biology 128(3): 529-535.

46. Bhosale, P., B. Serban, et al. 2007. Identification and Metabolic Transformations of Carotenoids in Ocular Tissues of the Japanese Quail *Coturnix japonica* Biochemistry 46(31): 9050-9057.

47. Siefermann-Harms, D., Hertzberg, S., Borch, G. and S. Liaaen-Jensen. 1981 Lactucaxanthin, an ε,ε-carotene-3,3′-diol from *Lactuca sativa*. Phytochemistry 20:85–88.

48. Bai, L., E. H. Kim, et al. 2009. Novel lycopene epsilon cyclase activities in maize revealed through perturbation of carotenoid biosynthesis. The Plant Journal 59(4): 588-599.

49. Asai, A., M. Terasaki, et al. 2004. An epoxide-furanoid rearrangement of spinach neoxanthin occurs in the gastrointestinal tract of mice and in vitro: formation and cytostatic activity of neochrome stereoisomers. J. Nutr. 134(9): 2237-2243.

50. Hallenstvet, M., E. Ryberg, et al. 1978. Animal carotenoids--XIV carotenoids of *Psammechinus miliaris* (sea-urchin). Comparative Biochemistry and Physiology Part B: Comparative Biochemistry 60(2): 173-175.

51. Yonekura, L., M. Kobayashi, Terasaki M., and A. Nagao. 2010 Keto-carotenoids are the major metabolites of dietary lutein and fucoxanthin in mouse tissues. J. Nutr. 140: 1824-1831

52. Tsushima, M. and T. Matsuno 1990. Comparative biochemical studies of carotenoids in sea-urchins-I. Comparative Biochemistry and Physiology Part B: Comparative Biochemistry 96(4): 801-810.

53. Fox, D. L. and T. S. Hopkins 1966. Comparative metabolic fractionation of carotenoids in three flamingo species. Comparative Biochemistry and Physiology 17(3): 841-856.

54. Toyoda, Y., L. R. Thomson, et al. 2002. Effect of Dietary Zeaxanthin on Tissue Distribution of Zeaxanthin and Lutein in Quail. Invest. Ophthalmol. Vis. Sci. 43(4): 1210-1221.

55. Arpin, N. and S. Liaaen-Jensen 1969. Carotenoids of higher plants--II: Rubixanthin and gazaniaxanthin. Phytochemistry 8(1): 185-193.

56. Deviche, P., K. J. McGraw, et al. 2008. Season-, sex-, and age-specific accumulation of plasma carotenoid pigments in free-ranging white-winged crossbills Loxia leucoptera. Journal of Avian Biology 39(3): 283-292.

57. Inouye C.Y., G. E. Hill, Stradi R.D. and R. Montgomerie 2001. Carotenoid pigments in male house finch plumage in relation to age, subspecies, and ornamental coloration. Auk 118(4): 900-915.

58. Valadon, L. R. G. and R. S. Mummery 1969. Changes in Carotenoid Composition of Certain Roses with Age. Annals of Botany 33(4): 671-677.

59. Umeno, D., A. V. Tobias, et al. 2005. Diversifying Carotenoid Biosynthetic Pathways by Directed Evolution. Microbiol. Mol. Biol. Rev. 69(1): 51-78.

60. Niklitschek, M., J. Alcaino, et al. 2008. Genomic organization of the structural genes controlling the astaxanthin biosynthesis pathway of Xanthophyllomyces dendrorhous. Biological Research 41: 93-108.

61. Cunningham, F. X., Jr., H. Lee, et al. 2007. Carotenoid Biosynthesis in the Primitive Red Alga Cyanidioschyzon merolae. Eukaryotic Cell 6(3): 533-545.

62. Sandmann, G. 1994. Carotenoid biosynthesis in microorganisms and plants. European Journal of Biochemistry 223(1): 7-24.

63. Ladygin, V. G. 2000. Biosynthesis of Carotenoids in the Chloroplasts of Algae and Higher Plants. Russian Journal of Plant Physiology 47(6): 796-814.

64. Leuenberger, F., Thommen, H . 1970. Keto-carotenoids in the Colorado beetle Leptinotarsa decemlineata . l. Insect Physiol. 16: 1 855-58.

65. Kayser 1977 Conversion of [C^{14}]β-carotene to its 2-hydroxy and 3-hydroxy metabolites by two moth species. Comp Biochem Physiol. 59: 177-181.

66. Katayama T., Kamata T., Shimaya M., Deshimaru O. and Chichester C. O. (1972) The biosynthesis of astaxanthin--VIII. The conversion of labelled β-carotene-15,15'-$^3H_2$ into astaxanthin in prawn, *Penaeus japonicus* Bat3. *Nippon Suisan Gakkaishi,* 38, 1171-1175.

67. KEGG (reaction R)1851, enzyme 1. 14.13.12 9)

68. Maoka T. 2011 Carotenoids in Marine Animals. 9:278-293293. Mar Drug.

69. Heller K.G., Fleischmann P. and Lutz-Röder A. 2000 Carotenoids in the spermatophores of bushcrickets (Orthoptera: Ephippigerinae). Proceed Royal Soc. 267: 1905-1908.

70. Goodwin T.W. 1984 The biochemistry of the carotenoids. Vol. 1. Plants. Chapman and Hall Eds

71. Goodwin T.W. 1984 The biochemistry of the carotenoids. Vol. 2. Animals. Chapman and Hall Eds.

72. Fox D.L., McBeth, J.W., and G. Mackinney. 1970 Some dietary carotenoids and blood-carotenoid levels in flamingos-II. γ-carotene and α-carotene consumed by the American flamingo. Comparative Biochemistry and Physiology 36:253-262.

73. Choi, S.-K., H. Harada, S. Matsuda, and N. Misawa. 2007. Characterization of two β-carotene ketolases, CrtO and CrtW, by complementation analysis in *Escherichia coli*. Applied Microbiology and Biotechnology 75:1335-1341.

74. Cunningham, F. X., and E. Gantt. 1998. Genes and enzymes of carotenoid biosynthesis in plants. Annu Rev Plant Physiol Mol Biol 49:557 - 583.

75. Fraser, P. D., Y. Miura, and N. Misawa. 1997. In vitro characterization of astaxanthin biosynthetic enzymes. Journal of Biological Chemistry 272:6128-6135.

76. Ravanello, M. P., D. Ke, J. Alvarez, B. Huang, and C. K. Shewmaker. 2003. Coordinate expression of multiple bacterial carotenoid genes in canola leading to altered carotenoid production. Metabolic Engineering 5:255-263.

77. Katayama, T., S. Makoto, S. Muneo, and C.O. Chichester. 1973. The biosynthesis of astaxanthin. XII. The conversion of labelled β-carotene-15, 15 $^3H_2$ into body astaxanthin in the lobster, *Panulirus japonicus*. International Journal of Biochemistry 4:223-226.

78. Katayama, T., Y. Kunisaki, M. Shimaya, K. L. Simpson, and C. O. Chichester. 1973a. The biosynthesis of astaxanthin--XIV. The conversion of labelled [beta]-carotene-15,15'-$^3H_2$ into astaxanthin in the crab, *Portunus trituberculatus*. Comparative Biochemistry and Physiology Part B: Comparative Biochemistry 46:269-272.

79. Withers, N. W., R. S. Alberte, R. A. Lewin, J. P. Thornber, G. Britton, and T. W. Goodwin. 1978. Photosynthetic unit size, carotenoids, and chlorophyll-protein composition of prochloron sp., a prokaryotic green alga. Proceedings of the National Academy of Sciences of the United States of America 75:2301-2305.

80. Cunningham, F. X., and E. Gantt. 2005. A study in scarlet: enzymes of ketocarotenoid biosynthesis in the flowers of Adonis aestivalis. The Plant Journal 41:478-492.

81. Hsieh, L. K., T.-C. Lee, C. O. Chichester, and K. L. Simpson. 1974. Biosynthesis of Carotenoids in Brevibacterium sp. KY-4313. Journal of Bacteriology 118:385-393.

82. Matsuno, T., K. Katagiri, T. Maoka, and T. Komori. 1985. Novel reductive metabolic pathways of 4-oxo-[beta]-end group in carotenoids of the spindle shell Fusinus perplexus. Comparative Biochemistry and Physiology Part B: Comparative Biochemistry 81:905-908.

83. Gilchrist, B. M., and W. L. Lee. 1976. The incorporation of [14C] β-carotene into the marine isopod *Idotea resecata* (Stimpson, 1857) and the biosynthesis of canthaxanthin. Comparative Biochemistry and Physiology Part B: Comparative Biochemistry 54:343-346.

84. Yuan, J.-P., and F. Chen. 1997. Identification of astaxanthin isomers in *Haematococcus lacustris* by HPLC-photodiode array detection. Biotechnology Techniques 11:455-459.

85. Schiedt, K., F. J. Leuenberger, and M. Vecchi. 1981. Natural occurrence of enantiomeric and meso-astaxanthin. 5. Ex wild salmon (*Salmo salar* and *Oncorhynchus*). Helvetica Chimica Acta 64:449-457.

86. Toomey, M. B., and K. J. McGraw. 2010. The effects of dietary carotenoid intake on carotenoid accumulation in the retina of a wild bird, the house finch (Carpodacus mexicanus). Archives of Biochemistry and Biophysics 504:161–168.

87. Cunningham, F. X., and E. Gantt. 2001. One ring or two? Determination of ring number in carotenoids by lycopene epsilon-cyclases. Proceedings of the National Academy of Sciences 98:2905-2910.

88. Hora, J., T. P. Toube, and B. C. L. Weedon. 1970. Carotenoids and related compounds. Part XXVII. Conversion of fucoxanthin into paracentrone. Journal of the Chemical Society C: Organic 2:241-242.

89. KEGG (reaction R07568, enzyme CtlZ)

90. Goodfellow D., Moss G. P. and B.C.L. Weedon. 1970. The Absolute Configuration of Lutein. J. Chem. Soc. D 13:1578-1578.

91. Khachik, F., P. S. Bernstein, and D. L. Garland. 1997. Identification of lutein and zeaxanthin oxidation products in human and monkey retinas. Investigative Ophthalmology & Visual Science 38:1802-11.

92. Hata, M., and M. Hata. 1971. Carotenoid pigments in goldfish (carassius auratus) II. colour change and carotenoid pigment composition. International Journal of Biochemistry 2:182-184.

93. Stradi, R., G. Celentano, M. Boles, and F. Mercato. 1997. Carotenoids in bird plumage: The pattern in a series of red-pigmented carduelinae. Comparative Biochemistry and Physiology Part B: Biochemistry and Molecular Biology 117:85-91.

94. Takaichi, S., K. Shimada, and J. Ishidsu. 1990. Carotenoids from the aerobic photosynthetic bacterium, *Erythrobacter longus*: β-Carotene and its hydroxyl derivatives. Archives of Microbiology 153:118-122.

95. Czeczuga, B. 1985. Carotenoids in representatives of the cladoniaceae. Biochemical Systematics and Ecology 13:83-88.

96. Cunningham, F. X. J., and G. E. 2011. Elucidation of the Pathway to Astaxanthin in the Flowers of *Adonis aestivalis*. The Plant Cell 23:3055-3069.

97. Takaichi, S., G. Sandmann, G. Schnurr, Y. Satomi, A. Suzuki, and N. Misawa. 1996. The carotenoid 7, 8-dihydro-ψ end group can be cyclized by the lycopene cyclases from the bacterium *Erwinia Uredovora* and the higher Plant *Capsicum Annuum*. European Journal of Biochemistry 241:291-296.

98. Gribanovski-Sassu, O. 1972. Effect of diphenylamine on carotenoid synthesis in *Dictyococcus cinnabarinus*. Phytochemistry 11:3195-3198.

99. Hudon, J., and A. H. Brush. 1990. Carotenoids produce flush in the elegant tern plumage. The Condor 92:798-801.

100. Schwartzel, E. M., and J. J. Cooney. 1972. Isolation of 4′-Hydroxyechinenone from Micrococcus roseus. Journal of Bacteriology 112: 1422-1424.

101. McGraw, K. J., P. M. Nolan, and O. L. Crino. 2006. Carotenoid accumulation strategies for becoming a colourful house finch: analyses of plasma and liver pigments in wild moulting birds. Functional Ecology 20:678-688.

102. Makino, T., H. Harada, H. Ikenaga, S. Matsuda, S. Takaichi, K. Shindoet al. (2008). Characterization of Cyanobacterial Carotenoid Ketolase CrtW and Hydroxylase CrtR by Complementation Analysis in Escherichia coli. Plant and Cell Physiology 49: 1867-1878.

103. Teruhisa Katayama, Y. K., Makoto Shimaya, K.L. Simpson and C.O. Chichester 1973. The biosynthesis of astaxanthin—XIV. The conversion of labelled β-carotene- 15,15′-3H2 into astaxanthin in the crab, *Portunus trituberculatus*. Comp Biochem 46: 269-272.

104. Stradi, R., E. Rossi, G. Celentano and B. Bellardi. 1996. Carotenoids in bird plumage: The pattern in three Loxia species and in Pinicola enucleator. Comparative Biochemistry and Physiology Part B: Biochemistry and Molecular Biology 113(2): 427-432.

105. Egeland, E. S., G. Johnsen, W. Eikrem, J. Throndsen, et al.1995. Pigments of *Bathycoccus prasinos* (Prasinophyceae): methodological and chemosystematic implications. J. PhycoL 31: 554-561.

106. Fernández, J. A. and J. Burgos.1981. Carotenoid pigments in the flesh and carapace of *Aristaeomorpha foliacea* and *Heterocarpus dorsalis* (crustacea: decapoda). Comparative Biochemistry and Physiology Part B: Comparative Biochemistry 69: 559-575.

107. Miki, W., K. Yamaguchi, S. Konosu, T. Takane, et al.1985. Origin of tunaxanthin in the integument of yellowtail (Seriola quinqueradiata). Comparative Biochemistry 80(2): 195-201.

108. Juola, F.A,. McGraw K. and Dearborn D.C, Carotenoids and throat pouch coloration in the great frigatebird (*Fregata minor*). Biochemistry and Molecular Biology 149:370-377.

109. Hata and Hata 1971. Carotenoid pigments in goldfish (*Carassius auratus* L.)-III. Metabolism of ingested cynthiaxanthin. Tohoku J. Agri. Res., 21 (19716), pp. 183–188

110. Buchecker, R. 1982 A chemist's view of animal carotenoids in: Carotenoid Chemistry and Biochemistry. G. Britton, T.W. and Goodwin (Eds.), Pergamon Press, Oxford (1982), p. 175-193.

111. Britton, G., Liaaen-Jensen, S and H. Pfander. 2004 Carotenoid. Handbook. Birkhäuser, Basel, Switzerland.

112. Czeczuga, B. and R. D. Worthington.1997. Carotenoids in lichens from the States of New Mexico and Texas in the United States of America. Feddes Repertorium 108: 387-399.

113. Goodwin, T. W. 1974. Algal physiology and biochemistry, Blackwell Scientific Publications Ltd.

114. Schiedt, K., S. Bischof and E. Glinz. 1993. Carotenoids. Part B. Metabolism, genetics, and biosynthesis, Harcourt Brace Jovanovich Publishers.

115. Han, Q., K. Shinohara, Y. Kakubari and Y. Mukai.2003. Photoprotective role of rhodoxanthin during cold acclimation in Cryptomeria japonica. Plant, Cell & Environment 26(5): 715-723.

116. Maoka, T., Y. Ito, Fujiwara and K. Hashimoto.1996. Structures and antioxidative activity of retro Carotenoids from the Berries of the Japanese yew, Taxus cuspidata. J. Jap. Oil Chem. Soc. 45: 641-646.

117. Hsu, W.-J., D. B. Rodriguez and C. O. Chichester. 1972. The biosynthesis of astaxanthin. VI. the conversion of [14C]lutein and [14C] β-carotene in goldfish. International Journal of Biochemistry 3: 333-338.

118. Hata, M. and Hata, M. 1972 Carotenoid pigments in goldfish - IV. Carotenoid metabolism, Bull. Jap. Soc. Sci. Fish., 38. 331-338.

119. Matsuno, T. and Katsuyama, M. 1982 Metabolism of zeaxanthin to rhodoxanthin in tilapia. Nippon Suisan Gakkaishi, 48 (1982), pp. 1491–1493.

120. Matsuno, T., Nagata, S., Iwahashi, M, Koike, T., Okada M. 1979. Intensification of color of fancy red carp with

zeaxanthin and myxoxanthophyll, major carotenoid constituents of spirulina. Bull. Jap. Soc. Scient. Fish., 45, pp. 627–632.

121. Guillou, A., Choubert, G., de la Noüe, J. 1992. Comparative accumulations of labelled carotenoids (14C-astaxanthin, 3H-canthaxanthin and 3H-zeaxanthin) and their metabolic conversions in mature female rainbow trout (*Oncorhynchus mykiss*). Comp. Biochem. Physiol. B. 102: 61-65.

122. Strand, A., O. Herstad and S. Liaaen-Jensen. 1998. Fucoxanthin metabolites in egg yolks of laying hens. Comparative Biochemistry and Physiology 119: 963-974.

123. McGraw, K. J., G. E. Hill, R. Stradi and R. S. Parker. 2002. The effect of dietary carotenoid access on sexual dichromatism and plumage pigment composition in the American goldfinch. Comparative Biochemistry and Physiology Part B: Biochemistry and Molecular Biology 131: 261-269.

124. Nitsche, H. (1974). Neoxanthin and fucoxanthinol in *Fucus vesiculosus*. Biochimica et Biophysica Acta (BBA) - General Subjects 338: 572-576.

125. Tian, L., Magallanes-Lundback, M., Musetti, V. and D. DellaPenna 2003 Functional Analysis of β- and ε-ring carotenoid hydroxylases in *Arabidopsis* The Plant Cell 15: 1320-1332.

126. Czeczuga, B. 1981. Carotenoids in fish. XXVIII. Carotenoids in *Micropterus salmoides* (Lalépéde) Centrarchidae. Hydrobiologia 78: 45-98.

127. Schiedt, K., Foss, P., Trond, S. and S. Liaaen-Jensen. 1989. Metabolism of carotenoids in salmonids-I. Idoxanthin, a metabolite of astaxanthin in the flesh of Atlantic salmon (*Salmo salar*, L.) under varying external conditions. Comparative Biochemistry and Physiology 92B: 277-281.

128. Hepperle, S.S., Li, Q. and A.L.L. East. 2005. Mechanism of cis/trans equilibration of alkenes via iodine catalysis. Journal of Physical Chemistry A 109: 10975-10981.

129. McDermott, J.C.B., Brown, D.J., Britton, G. and T.W. Goodwin. 1974. Alternative pathways of zeaxanthin biosynthesis in a *Flavobacterium* species. Experiments with nicotine as inhibitor. Biochemical Journal 144: 231-243.

130. Buchecker, R., Eugster, C.H. 1978. 183. Absolute konfiguration von α-doradexanthin und von frtischiellaxanthin, einem neuen carotenoid aus *Fritschiella tuberose* IYENG. Helvetica Chimica Acta 61: 1962-1968.

131. KEGG (reaction R07850, enzyme 1. 14. 99. 45).

200. Prum, R. O., A. M. LaFountain, J. Berro, M. C. Stoddard, and H. A. Frank. 2012. Molecular diversity, metabolic transformation, and evolution of carotenoid feather pigments in cotingas (Aves: Cotingidae). J Comp Physiol B 182:1095-1116.

201. Prum, R., A. LaFountain, C. Berg, M. Tauber, and H. Frank. 2014. Mechanism of carotenoid coloration in the brightly colored plumages of broadbills (Eurylaimidae). J Comp Physiol B 184:651-672.

202. LaFountain, A. M., H. A. Frank, and R. O. Prum. 2013. Carotenoids from the crimson and maroon plumages of Old World orioles (Oriolidae). Archives of Biochemistry and Biophysics 539:126-132.

203. LaFountain, A. M., S. Kaligotla, S. Cawley, K. M. Riedl, S. J. Schwartz, H. A. Frank, and R. O. Prum. 2010. Novel methoxy-carotenoids from the burgundy-colored plumage of the Pompadour Cotinga Xipholena punicea. Archives of Biochemistry and Biophysics 504:142-153.
