# Supplementary material for: Tradeoff between robustness and elaboration in carotenoid networks produces cycles of avian color diversification
Source: Biol Direct. 2015 Aug 20;10:45. doi: 10.1186/s13062-015-0073-6 (PMC4545997; doi:10.1186/s13062-015-0073-6)
Supplement: Additional file 3: Appendix S2. — Characteristic of carotenoid metabolic networks for species used in this study. References for Appendix S2. (PDF 205 kb) [file 13062_2015_73_MOESM3_ESM.pdf]

Appendix S2. Characteristics of carotenoid metabolic networks for species used in the study. See Supplementary Material for methods and details.

Common name	Scientific Name	Diet nodes	Nodes	Edges	Diam	Path	Degree	Diet diam	Cluster	Assort	Heter	Centr	Density	Modularity	Modules	Sensit/edge	Sens/node	Method	Main references
African-masked Weaver	Ploceus_velatus	2	2	0	0	0.00	0.00	0.00	0.00	.	.	.	0.00	0.00	2	.	0.50	HPLC	63
American Flamingo	Phoenicopterus_ruber	2	15	30	6	2.50	4.00	5.00	0.40	3.05	0.45	0.20	0.18	0.48	4	0.01	0.08	TLC	20, 24
American Goldfinch	Carduelis_tristis	4	7	8	3	1.42	2.29	3.00	0.24	2.73	0.75	0.30	0.29	0.11	4	0.00	0.14	HPLC	11, 48
American Redstart	Setophaga_ruticilla	2	9	16	2	1.30	3.56	2.00	0.62	2.74	0.28	0.25	0.31	0.50	2	0.01	0.22	OTH	38
Andean Flamingo	Phoenicopterus_andinus	3	13	23	4	1.89	3.54	4.00	0.37	3.02	0.49	0.27	0.19	0.50	4	0.01	0.17	TLC	20
Bananaquit	Coereba_flaveola	1	4	6	2	1.14	3.00	1.00	0.46	2.67	0.20	0.33	0.83	0.00	1	0.06	0.44	TLC	30
Baya Weaver	Ploceus_philippinus	2	2	0	0	0.00	0.00	0.00	0.00	.	.	.	0.00	0.00	2	.	0.50	HPLC	63
Beautiful Rosefinch	Carpodacus_pulcherrimus	4	16	25	6	2.34	3.13	6.00	0.18	2.81	0.53	0.29	0.15	0.50	4	0.03	0.11	HPLC	63, 67
Grey-headed Bullfinch	Pyrrhula_erythaca	3	12	22	5	1.86	3.67	3.00	0.40	2.98	0.38	0.27	0.23	0.50	3	0.01	0.10	HPLC	38, 63
Black Siskin	Carduelis_atrata	1	4	6	2	1.14	3.00	1.00	0.46	2.67	0.20	0.33	0.83	0.00	1	0.06	0.44	HPLC	62, 63
Black-and-yellow Grosbeak	Mycerobas_icteroides	1	1	0	0	0.00	0.00	0.00	0.00	.	.	.	0.00	0.00	1	.	1.00	OTH	38
Black-headed Bunting	Emberiza_melanocephala	2	2	0	0	0.00	0.00	0.00	0.00	.	.	.	0.00	0.00	2	.	0.50	HPLC	62, 63
Black-hooded Oriole	Oriolus_xanthornus	2	2	0	0	0.00	0.00	0.00	0.00	.	.	.	0.00	0.00	2	.	0.50	HPLC	63
Blue Tit	Cyanistes_caeruleus	2	2	0	0	0.00	0.00	0.00	0.00	.	.	.	0.00	0.00	2	.	0.50	HPLC	2, 62
Bohemian Waxwing	Bombycilla_garrulus	2	9	18	3	1.39	4.00	3.00	0.53	2.90	0.25	0.21	0.33	0.41	2	0.00	0.19	HPLC	62, 63
Brambling	Fringilla_montifringilla	1	1	0	0	0.00	0.00	0.00	0.00	.	.	.	0.00	0.00	1	.	1.00	OTH	38
Campo Flicker	Colaptes_campestris	3	3	0	0	0.00	0.00	0.00	0.00	.	.	.	0.00	0.00	3	.	0.33	HPLC	68
Cape Weaver	Ploceus_capensis	2	2	0	0	0.00	0.00	0.00	0.00	.	.	.	0.00	0.00	2	.	0.50	HPLC	63
Capercaillie	Tetrao_urogallus	2	6	10	2	1.38	3.33	2.00	0.63	2.80	0.58	0.60	0.40	0.11	3	0.00	0.31	TLC	16
Cardinal Quelea	Quelea_cardinalis	4	16	32	8	2.91	4.00	6.00	0.37	3.39	0.50	0.26	0.18	0.47	4	0.01	0.07	HPLC	63
Cedar Waxwing	Bombycilla_cedrorum	5	13	20	3	1.57	3.08	3.00	0.36	2.99	0.60	0.28	0.18	0.44	5	0.01	0.09	HPLC2	8, 11, 26, 62
Chaffinch	Fringilla_coelebs	4	12	20	4	1.83	3.33	4.00	0.19	2.61	0.53	0.31	0.20	0.39	4	0.01	0.09	HPLC	62
Chilean Flamingo	Phoenicopterus_chilensis	1	8	17	4	2.00	4.25	4.00	0.37	3.19	0.45	0.48	0.36	0.25	3	0.01	0.33	TLC	22
Citril Finch	Serinus_citrinella	1	4	6	2	1.14	3.00	1.00	0.46	2.67	0.20	0.33	0.83	0.00	1	0.06	0.44	HPLC	62, 65
Coal Tit	Periparus_ater	2	2	0	0	0.00	0.00	0.00	0.00	.	.	.	0.00	0.00	2	.	0.50	HPLC	62
Collared Grosbeak	Mycerobas_affinis	2	2	0	0	0.00	0.00	0.00	0.00	.	.	.	0.00	0.00	2	.	0.50	HPLC	63
Common Canary	Serinus_canaria	1	4	6	2	1.14	3.00	1.00	0.46	2.67	0.20	0.33	0.83	0.00	1	0.06	0.44	HPLC	4, 63
Common Redpoll	Carduelis_flammea	5	17	31	6	2.55	3.65	4.00	0.35	2.86	0.54	0.19	0.15	0.51	5	0.01	0.07	HPLC	62, 67, 69
Common Yellowthroat	Geothlypis_trichas	4	4	0	0	0.00	0.00	0.00	0.00	.	.	.	0.00	0.00	4	.	0.25	HPLC	11, 45
Cream-backed Woodpecker	Campephilus_leucopogon	4	16	32	8	2.91	4.00	6.00	0.37	3.39	0.50	0.26	0.18	0.47	4	0.01	0.07	HPLC	68
Crimson-backed Tanager	Ramphocelus_dimidiatus	4	14	29	6	2.58	4.14	4.00	0.43	3.44	0.52	0.22	0.20	0.43	4	0.00	0.07	HPLC2	11, 28
Dark-breasted Rosefinch	Carpodacus_nipalensis	3	12	24	4	1.80	4.00	4.00	0.33	3.08	0.35	0.26	0.24	0.44	3	0.00	0.08	HPLC	63
Desert Finch	Rhodospiza_obsoleta	4	16	32	8	2.91	4.00	6.00	0.37	3.39	0.50	0.26	0.18	0.47	4	0.01	0.07	HPLC	62, 63
Domestic Chicken	Gallus_gallus_domesticus	4	15	34	6	2.61	4.53	5.00	0.37	3.53	0.47	0.18	0.20	0.44	4	0.00	0.07	HPLC2	12, 72
Egyptian Vulture	Neophron_percnopterus	1	1	0	0	0.00	0.00	0.00	0.00	.	.	.	0.00	0.00	1	.	1.00	HPLC	51
Elegant Tern	Sterna_elegans	4	8	16	5	2.33	4.00	5.00	0.37	2.55	0.37	0.33	0.32	0.36	2	0.00	0.13	TLC	27
Eurasian Blackbird	Turdus_merula	5	5	0	0	0.00	0.00	0.00	0.00	.	.	.	0.00	0.00	5	.	0.20	HPLC	19
Eurasian Bullfinch	Pyrrhula_pyrrhula	4	19	37	8	2.97	3.89	6.00	0.31	3.29	0.47	0.21	0.15	0.54	4	0.02	0.08	HPLC	62, 69
Eurasian Siskin	Carduelis_spinus	1	4	6	2	1.14	3.00	1.00	0.46	2.67	0.20	0.33	0.83	0.00	1	0.06	0.44	HPLC	62, 64, 65
European Goldfinch	Carduelis_carduelis	3	10	18	3	1.39	3.60	3.00	0.48	2.90	0.43	0.22	0.27	0.41	3	0.00	0.16	HPLC2	28, 64, 65
European Greenfinch	Carduelis_chloris	2	5	8	3	1.42	3.20	3.00	0.33	2.73	0.33	0.25	0.60	0.11	2	0.00	0.20	HPLC	54, 60, 64, 65
European Serin	Serinus_serinus	2	9	16	2	1.30	3.56	2.00	0.62	2.74	0.28	0.25	0.31	0.50	2	0.01	0.22	HPLC	62, 64, 65
Evening Grosbeak	Coccothraustes_vespertina	1	1	0	0	0.00	0.00	0.00	0.00	.	.	.	0.00	0.00	1	.	1.00	HPLC	45
Forest Weaver	Ploceus_bicolor	2	2	0	0	0.00	0.00	0.00	0.00	.	.	.	0.00	0.00	2	.	0.50	HPLC	63
Franklin's Gull	Leucophaeus_pipixcan	1	1	0	0	0.00	0.00	0.00	0.00	.	.	.	0.00	0.00	1	.	1.00	HPLC	39
Gilded Flicker	Colaptes_chrysoides	3	11	19	6	2.29	3.45	5.00	0.27	3.15	0.55	0.44	0.24	0.35	3	0.02	0.12	HPLC	62
Goldcrest	Regulus_regulus	3	11	19	6	2.29	3.45	5.00	0.27	3.15	0.55	0.44	0.24	0.35	3	0.02	0.12	HPLC	62
Golden Bush-robin	Tarsiger_chrysaeus	2	3	3	2	1.25	2.00	1.00	0.00	1.67	0.35	1.00	0.67	0.00	1	0.00	0.33	HPLC	63
Golden Oriole	Oriolus_oriolus	2	2	0	0	0.00	0.00	0.00	0.00	.	.	.	0.00	0.00	2	.	0.50	HPLC	63
Golden-crowned Kinglet	Regulus_satrapa	3	18	39	9	3.01	4.33	7.00	0.37	3.48	0.38	0.21	0.17	0.51	3	0.01	0.06	HPLC	10
Golden-headed Manakin	Pipra_erythrocephala	4	16	32	8	2.91	4.00	6.00	0.37	3.39	0.50	0.26	0.18	0.47	4	0.01	0.07	TLC	29
Golden-winged Grosbeak	Rhynchostruthus_socotranus	1	4	6	2	1.14	3.00	1.00	0.46	2.67	0.20	0.33	0.83	0.00	1	0.06	0.44	OTH	38
Golden-winged Manakin	Masius_chrysopterus	2	6	9	3	1.59	3.00	3.00	0.24	3.07	0.47	0.50	0.47	0.12	2	0.03	0.20	HPLC	31
Gold-naped Finch	Pyrrhoplectes_epauletta	1	2	1	1	1.00	1.00	1.00	0.00	1.00	0.00	.	1.00	0.00	1	1.00	0.75	OTH	38
Gouldian Finch	Erythrura_gouldiae	2	12	21	5	2.40	3.50	4.00	0.25	2.84	0.35	0.27	0.23	0.40	3	0.04	0.16	HPLC	63
Great Frigatebird	Fregata_minor	4	5	4	2	1.20	1.60	2.00	0.00	1.40	0.33	0.33	0.30	0.44	2	0.50	0.20	HPLC	36
Great Spotted Woodpecker	Dendrocopos_major	3	11	19	6	2.29	3.45	5.00	0.27	3.15	0.55	0.44	0.24	0.35	3	0.02	0.12	HPLC	62, 68
Great Tit	Parus_major	2	3	1	1	1.00	0.67	1.00	0.00	1.00	0.71	0.50	0.33	0.00	2	1.00	0.44	HPLC3	15, 25, 33, 34, 56, 61, 62
Greater Flamingo	Phoenicopterus_roseus	1	8	17	4	2.00	4.25	4.00	0.37	3.19	0.45	0.48	0.36	0.25	3	0.01	0.33	TLC	22
Green Woodpecker	Picus_viridis	4	19	35	8	2.87	3.68	6.00	0.31	3.10	0.49	0.22	0.14	0.53	4	0.01	0.07	TLC	20
Green-barred Woodpecker	Colaptes_melanochloros	3	13	21	6	2.24	3.23	5.00	0.23	2.97	0.52	0.36	0.19	0.42	3	0.03	0.11	HPLC	62, 68

Grey Partridge	Perdix_perdix	2	8	13	4	1.97	3.25	3.00	0.00	2.81	0.56	0.38	0.29	0.15	4	0.04	0.31	TLC	12
Greylag Goose	Anser_anser	3	3	0	0	0.00	0.00	0.00	0.00	.	.	.	0.00	0.00	3	.	0.33	HPLC2	12, 50
Hairy Woodpecker	Picoides_villosus	4	20	36	8	2.85	3.60	6.00	0.26	3.11	0.50	0.21	0.13	0.52	4	0.02	0.07	HPLC	68
Hepatic Tanager	Piranga_flava	4	17	37	6	2.47	4.35	4.00	0.41	3.33	0.37	0.15	0.18	0.53	4	0.00	0.06	TLC	28
Hihi (Stitchbird)	Notiomystis_cincta	4	6	4	3	1.57	1.33	1.00	0.00	1.75	0.82	0.30	0.20	0.17	4	0.13	0.19	HPLC	17, 18
Hoary Redpoll	Carduelis_hornemanni	5	18	33	8	2.90	3.67	6.00	0.33	3.12	0.55	0.24	0.14	0.51	5	0.01	0.07	HPLC	67
Hooded Grosbeak	Coccothraustes_abeillei	1	1	0	0	0.00	0.00	0.00	0.00	.	.	.	0.00	0.00	1	.	1.00	OTH	38
House Finch	Carpodacus_mexicanus	6	21	46	9	3.04	4.00	8.00	0.27	3.32	0.50	0.17	0.12	0.55	6	0.01	0.06	HPLC2	3, 6, 32, 42, 70, 71
James's Flamingo	Phoenicopterus_jamesi	3	13	23	4	1.89	3.54	4.00	0.37	3.02	0.49	0.27	0.19	0.50	4	0.01	0.17	TLC	20
Japanese Waxwing	Bombycilla_japonica	2	9	18	3	1.39	4.00	3.00	0.53	2.90	0.25	0.21	0.33	0.41	2	0.00	0.19	HPLC	62
Japanese White-eye	Zosterops_japonicus	1	1	0	0	0.00	0.00	0.00	0.00	.	.	.	0.00	0.00	1	.	1.00	OTH	38
Korean Flycatcher	Ficedula_zanthopygia	2	2	0	0	0.00	0.00	0.00	0.00	.	.	.	0.00	0.00	2	.	1.00	HPLC	63
Lesser Flamingo	Phoenicopterus_minor	1	8	17	4	2.00	4.25	4.00	0.37	3.19	0.45	0.48	0.36	0.25	3	0.01	0.33	TLC	22
Lewis's Woodpecker	Melanerpes_lewis	4	16	32	8	2.91	4.00	6.00	0.37	3.39	0.50	0.26	0.18	0.47	4	0.01	0.07	HPLC	68
Linnet	Carduelis_cannabina	6	18	31	6	2.55	3.44	4.00	0.33	2.86	0.61	0.18	0.13	0.51	6	0.01	0.06	HPLC	3, 62, 69
Long-tailed Rosefinch	Uragus_sibiricus	3	13	20	6	2.27	3.08	6.00	0.23	2.82	0.60	0.38	0.18	0.42	4	0.02	0.10	HPLC	62, 63, 67, 69
Long Tailed Tit	Aegithalos_caudatus	1	2	1	1	1.00	1.00	1.00	0.00	1.00	0.00	.	1.00	0.00	1	1.00	0.75	HPLC	62, 63
Mallard	Anas_platyrhynchos	6	8	6	2	1.25	1.50	2.00	0.00	1.67	0.71	0.19	0.14	0.50	4	0.08	0.13	HPLC2	9, 12, 53
Nashville Warbler	Vermivora_ruficapilla	1	1	0	0	0.00	0.00	0.00	0.00	.	.	.	0.00	0.00	1	.	1.00	TLC	5
Nelicourvi Weaver	Ploceus_nelicourvi	2	2	0	0	0.00	0.00	0.00	0.00	.	.	.	0.00	0.00	2	.	0.50	HPLC	63
Northern Cardinal	Cardinalis_cardinalis	5	20	40	9	3.01	4.00	6.00	0.33	3.49	0.48	0.19	0.14	0.51	5	0.01	0.06	HPLC2	11, 28, 43, 46
Northern Oriole	Icterus_galbula	3	18	39	9	3.01	4.33	7.00	0.37	3.48	0.38	0.21	0.17	0.51	3	0.01	0.06	TLC	28
Orange Bullfinch	Pyrrhula_aurantiaca	1	4	6	2	1.14	3.00	1.00	0.46	2.67	0.20	0.33	0.83	0.00	1	0.06	0.44	OTH	38
Oriental Greenfinch	Carduelis_sinica	1	4	6	2	1.14	3.00	1.00	0.46	2.67	0.20	0.33	0.83	0.00	1	0.06	0.44	HPLC	65
Pallas' Rosefinch	Carpodacus_roseus	5	18	26	6	2.33	2.89	6.00	0.16	2.61	0.57	0.26	0.12	0.54	5	0.03	0.09	HPLC	62, 69
Palm Warbler	Dendroica_palmarum	1	1	0	0	0.00	0.00	0.00	0.00	.	.	.	0.00	0.00	1	.	1.00	OTH	38
Red-billed Leiothrix	Leiothrix_lutea	2	9	16	5	2.21	3.56	4.00	0.33	2.91	0.39	0.41	0.31	0.29	3	0.03	0.22	HPLC	62, 63
Pileated Woodpecker	Dryocopus_pileatus	3	15	23	6	2.20	3.07	5.00	0.15	2.84	0.52	0.31	0.16	0.45	3	0.03	0.11	HPLC	68
Pine Grosbeak	Pinicola_enucleator	4	14	20	5	1.89	2.86	3.00	0.21	2.43	0.54	0.27	0.15	0.51	4	0.03	0.10	HPLC	62, 63, 64, 66, 69
Pin-tailed Manakin	Ilicura_militaris	3	9	12	4	2.00	2.67	4.00	0.29	2.82	0.51	0.29	0.28	0.36	3	0.06	0.17	HPLC	31
Red Crossbill	Loxia_curvirostra	4	10	9	2	1.10	1.80	1.00	0.18	1.67	0.50	0.19	0.18	0.56	4	0.07	0.16	HPLC	13, 62, 64, 66, 69
Red Fody	Foudia_madagascariensis	4	16	32	8	2.91	4.00	6.00	0.37	3.39	0.50	0.26	0.18	0.47	4	0.01	0.07	HPLC	63
Red Munia	Amandava_amandava	3	5	4	3	1.57	1.60	1.00	0.00	1.75	0.62	0.33	0.30	0.17	3	0.13	0.24	HPLC	40
Red Siskin	Carduelis_cucullata	4	16	32	8	2.91	4.00	6.00	0.37	3.39	0.50	0.26	0.18	0.47	4	0.01	0.07	HPLC	62, 63
Red-backed Fairy-Wren	Malurus_melanocephalus	4	19	40	9	3.01	4.21	6.00	0.35	3.49	0.42	0.20	0.16	0.51	4	0.01	0.06	HPLC	57
Red-billed Quelea	Quelea_quelea	4	16	32	8	2.91	4.00	6.00	0.37	3.39	0.50	0.26	0.18	0.47	4	0.01	0.07	HPLC	63
Red-collared Widowbird	Euplectes_ardens	4	19	40	9	3.01	4.21	6.00	0.35	3.49	0.42	0.20	0.16	0.51	3	0.01	0.06	HPLC	1
Red-fronted Serin	Serinus_pusillus	2	9	16	2	1.30	3.56	2.00	0.62	2.74	0.28	0.25	0.31	0.50	2	0.01	0.22	HPLC	3, 62, 65
Red-headed Bullfinch	Pyrrhula_erythrocephala	1	4	6	2	1.14	3.00	1.00	0.46	2.67	0.20	0.33	0.83	0.00	1	0.06	0.44	OTH	38
Red-headed Manakin	Pipra_rubrocapilla	4	16	32	8	2.91	4.00	7.00	0.37	3.39	0.50	0.26	0.18	0.47	4	0.01	0.07	TLC	29
Red-headed Parrot Finch	Erythrura_psittacea	2	8	13	4	1.90	3.25	4.00	0.46	2.88	0.48	0.52	0.32	0.29	3	0.04	0.22	HPLC	63
Red-headed Quelea	Quelea_erythrops	4	16	32	8	2.91	4.00	6.00	0.37	3.39	0.50	0.26	0.18	0.47	4	0.01	0.07	HPLC	63
Red-shafted Flicker	Colaptes_auratus_cafer	4	16	32	8	2.91	4.00	6.00	0.37	3.39	0.50	0.26	0.18	0.47	4	0.01	0.07	HPLC	62
Fan-tailed Widowbird	Euplectes_axillaris	2	6	9	3	1.53	3.00	3.00	0.24	3.07	0.47	0.50	0.47	0.12	2	0.03	0.19	HPLC	1
Red-winged Blackbird	Agelaius_phoeniceus	4	17	37	6	2.47	4.35	4.00	0.41	3.33	0.37	0.15	0.18	0.53	4	0.00	0.06	HPLC	11, 38, 47
Ring-billed Gull	Larus_delawarensis	1	1	0	0	0.00	0.00	0.00	0.00	.	.	.	0.00	0.00	1	.	1.00	HPLC	39
Ring-necked Pheasant	Phasianus_colchicus	2	8	13	4	1.97	3.25	3.00	0.00	2.81	0.56	0.38	0.29	0.15	4	0.04	0.34	TLC	4, 12
Robin	Erithacus_rubecula	1	2	1	1	1.00	1.00	1.00	0.00	1.00	0.00	.	1.00	0.00	1	1.00	0.75	HPLC	63
Roseate Spoonbill	Platalea_ajaja	3	3	4	2	1.33	1.33	2.00	0.00	1.67	0.35	1.00	0.67	0.00	1	.	0.33	HPLC2	23, 63
Rose-breasted Grosbeak	Pheucticus_ludovicianus	4	14	29	6	2.58	4.29	4.00	0.43	3.44	0.52	0.22	0.20	0.43	4	0.00	0.07	HPLC2	11, 28
Round-tailed Manakin	Pipra_chloromeros	5	17	32	8	2.91	3.76	7.00	0.35	3.39	0.57	0.25	0.15	0.47	5	0.01	0.07	TLC	29
Saffron Finch	Sicalis_flaveola	1	1	0	0	0.00	0.00	0.00	0.00	.	.	.	0.00	0.00	1	.	1.00	OTH	38
Sakalava Weaver	Ploceus_sakalava	2	2	0	0	0.00	0.00	0.00	0.00	.	.	.	0.00	0.00	2	.	0.50	HPLC	63
Scaly-bellied Woodpecker	Picus_squamatus	1	3	2	2	1.33	1.33	2.00	0.00	1.67	0.35	1.00	0.67	0.00	1	0.75	0.67	HPLC	68
Scarlet Finch	Haematospiza_sipahi	5	18	33	8	2.90	3.67	6.00	0.33	3.12	0.55	0.24	0.14	0.51	5	0.01	0.07	HPLC	63
Scarlet Ibis	Eudocimus_ruber	1	1	0	0	0.00	0.00	0.00	0.00	.	.	.	0.00	0.00	1	.	1.00	TLC	21
Scarlet Minivet	Pericrocotus_flammeus	4	16	32	8	2.91	4.00	6.00	0.37	3.39	0.50	0.26	0.18	0.47	4	0.01	0.07	HPLC	62
Scarlet Tanager	Piranga_olivacea	4	19	40	9	3.01	4.21	6.00	0.35	3.49	0.42	0.20	0.16	0.51	4	0.01	0.06	TLC	11, 28
Siberian Rubythroat	Luscinia_calliope	4	16	32	8	2.91	4.00	6.00	0.37	3.39	0.50	0.26	0.18	0.47	4	0.01	0.07	HPLC	62, 63
Silver-eared Mesia	Leiothrix_argentauris	2	9	16	5	2.21	3.56	4.00	0.33	2.91	0.39	0.41	0.31	0.29	3	0.03	0.19	HPLC	63
Sooty-capped Bush Tanager	Chlorospingus_pileatus	1	1	0	0	0.00	0.00	0.00	0.00	.	.	.	0.00	0.00	1	.	1.00	OTH	35
Southern Red Bishop	Euplectes_orix	4	17	35	9	3.08	4.12	6.00	0.31	3.36	0.45	0.23	0.17	0.47	4	0.01	0.07	HPLC	55, 63
Spot-winged Grosbeak	Mycerobas_melanozanthos	1	1	0	0	0.00	0.00	0.00	0.00	.	.	.	0.00	0.00	1	.	1.00	OTH	38
Star Finch	Neochmia_ruficauda	2	2	0	0	0.00	0.00	0.00	0.00	.	.	.	0.00	0.00	1	.	0.50	HPLC	40

Streaked Rosefinch	Carpodacus_rubicilloides	2	4	2	1	1.00	1.00	1.00	0.00	1.00	0.00	0.00	0.33	0.50	2	0.50	0.38	HPLC	63, 67, 69
Sulfur-breasted Bushrike	Telophorus_sulfureopectus	2	11	21	5	2.10	3.82	4.00	0.40	3.22	0.35	0.28	0.27	0.40	3	0.02	0.14	HPLC	63
Summer Tanager	Piranga_rubra	1	5	10	2	1.38	4.00	2.00	0.75	2.80	0.33	0.67	0.60	0.11	2	0.00	0.44	TLC	28
Three-banded Rosefinch	Carpodacus_trifasciatus	3	10	17	4	1.88	3.40	4.00	0.30	2.71	0.53	0.39	0.24	0.33	4	0.01	0.11	HPLC	63, 67
Three-toed Woodpecker	Picoides_tridactylus	2	2	0	0	0.00	0.00	0.00	0.00	.	.	.	0.00	0.00	2	.	0.50	HPLC	68
Toco Toucan	Ramphastos_toco	1	3	2	2	1.33	1.33	2.00	0.00	1.67	0.35	1.00	0.67	0.00	1	0.75	0.67	HPLC	63
Tristan Bunting	Nesospiza_acunhae	2	6	9	3	1.53	3.00	3.00	0.24	3.07	0.47	0.50	0.47	0.12	2	0.03	0.19	TLC	58
Trumpeter Finch	Bucanetes_githagineus	4	16	32	8	2.91	4.00	6.00	0.37	3.39	0.50	0.26	0.18	0.47	4	0.01	0.07	HPLC	63
Village Weaver	Ploceus_cucullatus	2	2	0	0	0.00	0.00	0.00	0.00	.	.	.	0.00	0.00	2	.	0.50	HPLC	4, 63
Virginia's Warbler	Vermivora_virginiae	1	1	0	0	0.00	0.00	0.00	0.00	.	.	.	0.00	0.00	1	.	1.00	TLC	5
Wallcreeper	Tichodroma_muraria	1	5	10	2	1.38	4.00	2.00	0.75	2.80	0.33	0.67	0.60	0.11	2	0.00	0.44	HPLC	62
Western Tanager	Piranga_ludoviciana	2	5	6	2	1.14	2.40	1.00	0.37	2.67	0.55	0.42	0.50	0.00	2	0.06	0.32	TLC	28
White Stork	Ciconia_ciconia	2	2	0	0	0.00	0.00	0.00	0.00	.	.	.	0.00	0.00	2	.	0.50	TLC	49
White Woodpecker	Melanerpes_candidus	2	6	4	2	1.33	1.64	2.00	0.00	1.97	0.35	0.20	0.27	0.50	2	0.38	0.33	HPLC	68
White-browed Rosefinch	Carpodacus_thura	4	13	19	5	1.91	2.92	3.00	0.23	2.47	0.56	0.30	0.17	0.47	4	0.02	0.10	HPLC	63
White-winged Crossbill	Loxia_leucoptera	6	21	39	6	2.45	3.71	4.00	0.33	2.88	0.48	0.14	0.12	0.58	6	0.00	0.05	HPLC2	14, 28, 62, 66
White-winged Grosbeak	Mycerobas_carnipes	1	1	0	0	0.00	0.00	0.00	0.00	.	.	.	0.00	0.00	1	.	1.00	OTH	38
Wild Turkey	Meleagris_gallopavo	4	9	12	3	1.64	2.67	2.00	0.41	3.00	0.79	0.52	0.22	0.21	5	0.02	0.15	TLC	12
Yellow Bishop	Euplectes_capensis	2	2	0	0	0.00	0.00	0.00	0.00	.	.	.	0.00	0.00	2	.	0.50	HPLC	63
Yellow Wagtail	Motacilla_flava	2	2	0	0	0.00	0.00	0.00	0.00	.	.	.	0.00	0.00	2	.	0.50	HPLC	63
Yellow Warbler	Dendroica_petechia	4	4	0	0	0.00	0.00	0.00	0.00	.	.	.	0.00	0.00	4	.	0.25	HPLC	45
Yellow-bellied Sapsucker	Sphyrapicus_varius	4	18	34	8	2.88	3.78	6.00	0.33	3.23	0.49	0.23	0.15	0.52	4	0.01	0.07	HPLC	68
Yellow-breasted Chat	Icteria_virens	1	1	0	0	0.00	0.00	0.00	0.00	.	.	.	0.00	0.00	1	.	1.00	HPLC	37
Yellow-breasted Greenfinch	Carduelis_spinoides	1	4	6	2	1.14	3.00	1.00	0.46	2.67	0.20	0.33	0.83	0.00	1	0.06	0.44	HPLC	65
Yellow-cheeked Tit	Parus_spilonotus	2	2	0	0	0.00	0.00	0.00	0.00	.	.	.	0.00	0.00	2	.	0.50	HPLC	63
Yellow-crowned Bishop	Euplectes_afer	2	3	3	2	1.25	2.00	1.00	0.00	1.67	0.35	1.00	0.67	0.00	1	0.00	0.33	HPLC	55, 63
Yellow-fronted Canary	Serinus_mozambicus	1	4	6	2	1.14	3.00	1.00	0.46	2.67	0.20	0.33	0.83	0.00	1	0.06	0.44	HPLC	62, 63
Yellowhammer	Emberiza_citrinella	2	2	0	0	0.00	0.00	0.00	0.00	.	.	.	0.00	0.00	2	.	0.50	HPLC	62, 63
Yellow-legged Gull	Larus_michahellis	6	10	13	5	2.21	2.60	4.00	0.00	1.89	0.42	0.06	0.18	0.41	4	0.00	0.10	HPLC	52, 59
Yellow-mantled Widowbird	Euplectes_macrourus	2	5	8	3	1.42	3.20	3.00	0.33	2.73	0.33	0.25	0.60	0.11	2	0.04	0.20	HPLC	1
Yellow-rumped Warbler	Dendroica_coronata	1	1	0	0	0.00	0.00	0.00	0.00	.	.	.	0.00	0.00	1	.	1.00	OTH	38
Yellow-shafted Flicker	Colaptes_auratus	4	18	34	8	2.88	3.78	6.00	0.33	3.23	0.49	0.23	0.15	0.52	4	0.01	0.07	HPLC	11, 62, 68
Zebra Finch	Taeniopygia_guttata	4	18	36	9	3.06	4.00	6.00	0.29	3.38	0.47	0.22	0.16	0.48	4	0.01	0.07	HPLC	41, 44
Zebra Waxbill	Amandava_subflava	3	5	4	3	1.57	1.60	1.00	0.00	1.75	0.62	0.33	0.30	0.17	3	0.13	0.24	HPLC	40
**FULL AVIAN NETWORK**	.	.	44	78	11	4.64	3.55	8.00	0.15	3.33	0.61	0.13	0.06	0.65	9	0.01	0.04	.	.

Method:

HPLC High-performance liquid chromatography
TLC Thin layer chromatography
HPLC2 HPLC and TLC combined
HPLC3 HPLC and Mass spectrometry
OTH Mass spectrometry, others

Literature Sources for Appendix S2:

1 Andersson, S., Prager, M. & Johansson, E. I. A. Carotenoid content and reflectance of yellow and red nuptial plumages in widowbirds (Euplectes spp.). Functional Ecology 21, 272-281 (2007).

2 Arnold, K. E., Ramsay, S. L., Henderson, L. & Larcombe, S. D. Seasonal variation in diet quality: antioxidants, invertebrates and blue tits Cyanistes caeruleus. Biological Journal of the Linnean Society 99, 708-717 (2010).

3 Badyaev, A. V., Belloni, V., Kennedy, L. & Delaney, R. (unpubl. data).

4 Brockmann, H. & Völker, O. Der gelbe Federfarbstoff des Kanarienvogels [Serinus canaria canaria (L.)] und das Vorkommen von Carotinoiden bei Vögeln. Hoppe-Seyler's Zeitschrift für physiologische Chemie 224, 193-215 (1934).

5 Brush, A. H. & Johnson, N. K. The Evolution of Color Differences between Nashville and Virginia's Warblers. Condor 78, 412-414 (1976).

6 Brush, A. H. & Power, D. M. House finch pigmentation: carotenoid metabolism and the effect of diet. Auk 93, 725-739 (1976).

7 Brush, A. H. Pigmentation in the scarlet tanager, Piranga olivacea. Condor 69, 549-559 (1967).

8 Brush, A. H. & Allen, K. Astaxanthin in the Cedar Waxwing. Science 142, 47-48 (1963).

9 Butler, M. W. & McGraw, K. J. Relationships between dietary carotenoids, body tissue carotenoids, parasite burden, and health state in wild mallard (Anas platyrhynchos) ducklings. Arch Biochem Biophys 504, 154-160, doi:10.1016/j.abb.2010.07.003 (2010).

10 Chui, C. K. S., McGraw, K. J. & Doucet, S. M. Carotenoid-based plumage coloration in golden-crowned kinglets Regulus satrapa: pigment characterization and relationships with migratory timing and condition. Journal of Avian Biology 42, 309-322 (2011).

11 Cohen, A. A., McGraw, K. J. & Robinson, W. D. Serum antioxidant levels in wild birds vary in relation to diet, season, life history strategy, and species. Oecologia 161, 673-683, doi:10.1007/s00442-009-1423-9 (2009).

12 Czeczuga, B. Carotenoids in the skin of certain species of birds. Comparative Biochemistry and Physiology Part B: Comparative Biochemistry 62, 107-109 (1979).

13 del Val, E. et al. The liver but not the skin is the site for conversion of a red carotenoid in a passerine bird. Naturwissenschaften 96, 797-801, doi:10.1007/s00114-009-0534-9 (2009).

14 Deviche, P., McGraw, K. J. & Underwood, J. Season-, sex-, and age-specific accumulation of plasma carotenoid pigments in free-ranging white-winged crossbills Loxia leucoptera. Journal of Avian Biology 39, 283-292 (2008).

15 Eeva, T., Sillanpää, S. & Salminen, J. P. The effects of diet quality and quantity on plumage colour and growth of great tit Parus major nestlings: a food manipulation experiment along a pollution gradient. Journal of Avian Biology 40, 491-499 (2009).

16 Egeland, E. S., Parker, H. & Liaaenjensen, S. Carotenoids in combs of Capercaillie (Tetrao urogallus) fed defined diets. Poultry Science 72, 747-751 (1993).

17 Ewen, J. G. et al. Carotenoids, colour and conservation in an endangered passerine, the hihi or stitchbird (Notiomystis cincta). Anim Conserv 9, 229-235 (2006).

18 Ewen, J. G., Thorogood, R., Karadas, F., Pappas, A. C. & Surai, P. F. Influences of carotenoid supplementation on the integrated antioxidant system of a free living endangered passerine, the hihi (Notiomystis cincta). Comparative Biochemistry and Physiology - Part A: Molecular & Integrative Physiology 143, 149-154 (2006).

19 Faivre, B., Gregoire, A., Preault, M., Cezilly, F. & Sorci, G. Immune activation rapidly mirrored in a secondary sexual trait. Science 300, 103 (2003).

20 Fox, D. L. & Hopkins, T. S. Comparative metabolic fractionation of carotenoids in three flamingo species. Comparative Biochemistry and Physiology 17, 841-856 (1966).

21 Fox, D. L. Carotenoids of the scarlet ibis. Comparative Biochemistry and Physiology 5, 31-43 (1962).

22 Fox, D. L., Smith, V. E. & Wolfson, A. A. Carotenoid selectivity in blood and feathers of lesser (African), chilean and greater (European) flamingos. Comparative Biochemistry and Physiology 23, 225-232 (1967).

23 Fox, D. L., Hopkins, T. S. & Zilversmit, D. B. Blood carotenoids of the roseate spoonbill. Comparative Biochemistry and Physiology 14, 641-649 (1965).

24 Fox, D. L., Wolfson, A. A. & McBeth, J. W. Metabolism of b-carotene in the American flamingo, Phoenicopterus ruber. Comparative Biochemistry and Physiology 29, 1223-1229 (1969).

25 Hõrak, P., Surai, P. F., Ots, I. & Møller, A. P. Fat soluble antioxidants in brood-rearing great tits Parus major: relations to health and appearance. Journal of Avian Biology 35, 63-70 (2004).

26 Hudon, J. & Brush, A. H. Probably dietary basis of a color variant of the cedar waxwing. Journal of Field Ornithology 60, 361-368 (1989).

27 Hudon, J. & Brush, A. H. Carotenoids produce flush in the elegant tern plumage. Condor 92, 798-801 (1990).

28 Hudon, J. Unusual carotenoid use by western tanager (Piranga ludoviviana) and its evolutionary implications. Canadian Journal of Zoology 69, 2311-2320 (1991).

29 Hudon, J., Capparella, A. P. & Brush, A. H. Plumage pigment differences in manakins of the Pipra erythrocephala superspecies Auk 106, 34-41 (1989).

30 Hudon, J., Ouellet, H., Bénito-Espinal, É. & Brush, A. H. Characterization of an Orange Variant of the Bananaquit (Coereba flaveola) on La Désirade, Guadeloupe, French West Indies. Auk 113, 715-718 (1996).

31 Hudon, J., Anciaes, M., Bertacche, V. & Stradi, R. Plumage carotenoids of the Pin-tailed Manakin (Ilicura militaris): evidence for the endogenous production of rhodoxanthin from a colour variant. Comparative biochemistry and physiology. Part B, Biochemistry & molecular biology 147, 402-411, doi:10.1016/j.cbpb.2007.02.004 (2007).

32 Inouye, C. Y., Hill, G. E., Stradi, R. D., Montgomerie, R. & Bosque, C. Carotenoid pigments in male house finch plumage in relation to age, subspecies, and ornamental coloration. Auk 118, 900-915 (2001).

33 Isaksson, C., Ornborg, J., Prager, M. & Andersson, S. Sex and age differences in reflectance and biochemistry of carotenoid-based colour variation in the great tit Parus major. Biological Journal of the Linnean Society 95, 758-765 (2008).

34 Isaksson, C., Sturve, J., Almroth, B. C. & Andersson, S. The impact of urban environment on oxidative damage (TBARS) and antioxidant systems in lungs and liver of great tits, Parus major. Environ Res 109, 46-50 (2009).

35 Johnson, N. K. & Brush, A. H. Analysis of Polymorphism in the Sooty-Capped Bush Tanager. Systematic Zoology 21, 245-262 (1972).

36 Juola, F. A., McGraw, K. J. & Dearborn, D. C. Carotenoids and throat pouch coloration in the great frigatebird (Fregata minor). Comparative Biochemistry and Physiology Part B: Biochemistry and Molecular Biology 149, 370-377 (2008).

37 Mays Jr, H. L. et al. Sexual dichromatism in the yellow-breasted chat Icteria virens: spectrophotometric analysis and biochemical basis. Journal of Avian Biology 35, 125-134 (2004).

38 McGraw, K. J. in Bird Coloration. I. Mechanisms and Measurements (eds G.E. Hill & K. J. McGraw) 177-242 (Harvard University Press, 2006).
39 McGraw, K. J. & Hardy, L. S. Astaxanthin is responsible for the pink plumage flush in Franklin's and Ring-billed gulls. Journal of Field Ornithology 77, 29-33 (2006).
40 McGraw, K. J. & Schuetz, J. G. The evolution of carotenoid coloration in estrildid finches: a biochemical analysis Comp. Biochem. Physiol. B 139, 45-51 (2004).
41 McGraw, Kevin J. & Toomey, Matthew B. Carotenoid Accumulation in the Tissues of Zebra Finches: Predictors of Integumentary Pigmentation and Implications for Carotenoid Allocation Strategies. Physiological and Biochemical Zoology 83, 97-109, doi:doi:10.1086/648396 (2010).
42 McGraw, K. J., Nolan, P. M. & Crino, O. L. Carotenoid accumulation strategies for becoming a colourful House Finch: analyses of plasma and liver pigments in wild moulting birds. Functional Ecology 20, 678-688 (2006).
43 McGraw, K. J., Hill, G. E., Stradi, R. & Parker, R. S. The Influence of Carotenoid Acquisition and Utilization on the Maintenance of Species-Typical Plumage Pigmentation in Male American Goldfinches (Carduelis tristis) and Northern Cardinals (Cardinalis cardinalis). Physiological and Biochemical Zoology 74, 843-852, doi:doi:10.1086/323797 (2001).
44 McGraw, K. J., Adkins-Regan, E. & Parker, R. S. Anhydrolutein in the zebra finch: a new, metabolically derived carotenoid in birds. Comparative Biochemistry and Physiology Part B: Biochemistry and Molecular Biology 132, 811-818 (2002).
45 McGraw, K. J., Beebee, M. D., Hill, G. E. & Parker, R. S. Lutein-based plumage coloration in songbirds is a consequence of selective pigment incorporation into feathers. Comparative Biochemistry and Physiology Part B: Biochemistry and Molecular Biology 135, 689-696 (2003).
46 McGraw, K. J., Hill, G. E. & Parker, R. S. Carotenoid pigments in a mutant cardinal: Implications for the genetic and enzymatic control mechanisms of carotenoid metabolism in birds. Condor 105, 587-592 (2003).
47 McGraw, K. J., Wakamatsu, K., Clark, A. B. & Yasukawa, K. Red-winged blackbirds Agelaius phoeniceus use carotenoid and melanin pigments to color their epaulets. Journal of Avian Biology 35, 543-550 (2004).
48 McGraw, K. J., Hill, G. E. & Parker, R. S. The physiological costs of being colourful: nutritional control of carotenoid utilization in the American goldfinch, Carduelis tristis. Animal Behaviour 69, 653-660, doi:10.1016/j.anbehav.2004.05.018 (2005).
49 Negro, J. J. & Garrido-Fernández, J. Astaxanthin is the major carotenoid in tissues of white storks (Ciconia ciconia) feeding on introduced crayfish (Procambarus clarkii). Comparative Biochemistry and Physiology Part B: Biochemistry and Molecular Biology 126, 347-352 (2000).
50 Negro, J. J., Tella, J. L., Hiraldo, F., Bortolotti, G. R. & Prieto, P. Sex- and age-related variation in plasma carotenoids despite a constant diet in the red-legged partridge (Alectoris rufa). Ardea 89, 275-279 (2001).
51 Negro, J. J. et al. Coprophagy: An unusual source of essential carotenoids. Nature 416, 807-808 (2002).
52 Pérez, C., Lores, M. & Velando, A. Availability of nonpigmentary antioxidant affects red coloration in gulls. Behavioral Ecology 19, 967-973, doi:10.1093/beheco/arn053 (2008).
53 Peters, A., Delhey, K., Denk, A. G. & Kempenaers, B. Tarde-offs between immune investment and sexual signaling in male mallards. American Naturalist 164, 51-59 (2004).
54 Peters, A., Delhey, K., Andersson, S., Van Noordwijk, H. & Förschler, M. I. Condition-dependence of multiple carotenoid-based plumage traits: an experimental study. Functional Ecology 22, 831-839 (2008).
55 Prager, M., Johansson, E. I. & Andersson, S. Differential ability of carotenoid C4-oxygenation in yellow and red bishop species (Euplectes spp.). Comparative biochemistry and physiology. Part B, Biochemistry & molecular biology 154, 373-380, doi:10.1016/j.cbpb.2009.06.015 (2009).
56 Quesada, J. & Senar, J. C. Comparing plumage colour measurements obtained directly from live birds and from collected feathers: the case of the great tit Parus major. Journal of Avian Biology 37, 609-616 (2006).
57 Rowe, M. & McGraw, K. J. Carotenoids in the Seminal Fluid of Wild Birds: Interspecific Variation in Fairy-Wrens. The Condor 110, 694-700, doi:10.1525/cond.2008.8604 (2008).
58 Ryan, P. G., Moloney, C. L. & Hudon, J. Color variation and hybridization among Nesospiza Buntings on inaccessible islands, Tristan da Cunha. Auk 111, 314-327 (1994).
59 Saino, N., Bertacche, V., Bonisoli-Alquati, A., Romano, M. & Rubolini, D. Phenotypic Correlates of Yolk and Plasma Carotenoid Concentration in Yellow-Legged Gull Chicks. Physiological and Biochemical Zoology 81, 211-225, doi:doi:10.1086/527454 (2008).
60 Saks, L., McGraw, K. & Hõrak, P. How feather colour reflects its carotenoid content. Functional Ecology 17, 555-561 (2003).
61 Sillanpää, S., Salminen, J.-P. & Eeva, T. Breeding success and lutein availability in great tit (Parus major). Acta Oecologica 35, 805-810 (2009).
62 Stradi, R. The Colour of Flight. (Solei Gruppos Editoriale Informatico, 1998).
63 Stradi, R. in Colori in volo - il piumaggio degli uccelli (eds l. Brambilla, G. Canali, Mannucci E., & et al.) 117-146 (Università degli Studi di Milano 1999).
64 Stradi, R., Celentano, G. & Nava, D. Separation and identification of carotenoids in bird's plumage by high-performance liquid chromatography-diode-array detection. Journal of Chromatography B: Biomedical Sciences and Applications 670, 337-348 (1995).
65 Stradi, R., Celentano, G., Rossi, E., Rovati, G. & Pastore, M. Carotenoids in bird plumage: I. The carotenoid pattern in a series of Palearctic Carduelinae Comparative Biochemestry Physiology Part B: Comparative Biochemistry and Physiology 110, 131 -143 (1995).
66 Stradi, R., Rossi, E., Celentano, G. & Bellardi, B. Carotenoids in bird plumage: the pattern in three Loxia species and in Pinicola enucleator. Comparative Biochemistry and Physiology Part B: Biochemistry and Molecular Biology 113, 427-432 (1996).
67 Stradi, R., Celentano, G., Boles, M. & Mercato, F. Carotenoids in Bird Plumage: The Pattern in a Series of Red-Pigmented Carduelinae. Comparative Biochemistry and Physiology Part B: Biochemistry and Molecular Biology 117, 85-91 (1997).
68 Stradi, R., Hudon, J., Celentano, G. & Pini, E. Carotenoids in bird plumage: the complement of yellow and red pigments in true woodpeckers (Picinae). Comparative Biochemistry and Physiology Part B: Biochemistry and Molecular Biology 120, 223-230 (1998).
69 Stradi, R., Pini, E. & Celentano, G. Carotenoids in bird plumage: the complement of red pigments in the plumage of wild and captive bullfinch (Pyrrhula pyrrhula). Comparative Biochemistry and Physiology Part B 128, 529-535 (2001).
70 Toomey, M. B. & McGraw, K. J. Seasonal, sexual, and quality related variation in retinal carotenoid accumulation in the house finch (Carpodacus mexicanus). Functional Ecology 23, 321-329 (2009).

71 Toomey, M. B. & McGraw, K. J. The effects of dietary carotenoid intake on carotenoid accumulation in the retina of a wild bird, the house finch (Carpodacus mexicanus). Arch Biochem Biophys 504, 161-168 (2010).
72 Tyczkowski, J. K., Yagen, B. & Hamilton, P. B. Metabolism of canthaxanthin, a red diketocarotenoid, by chickens. Poultry Science 67, 787-793 (1988).
