# Supplementary material for: Tradeoff between robustness and elaboration in carotenoid networks produces cycles of avian color diversification
Source: Biol Direct. 2015 Aug 20;10:45. doi: 10.1186/s13062-015-0073-6 (PMC4545997; doi:10.1186/s13062-015-0073-6)
Supplement: Additional file 5: Figure S3. — Distribution of Robinson-Foulds distances between 10,000 random trees and the molecular-based supertree. (PDF 128 kb) [file 13062_2015_73_MOESM5_ESM.pdf]

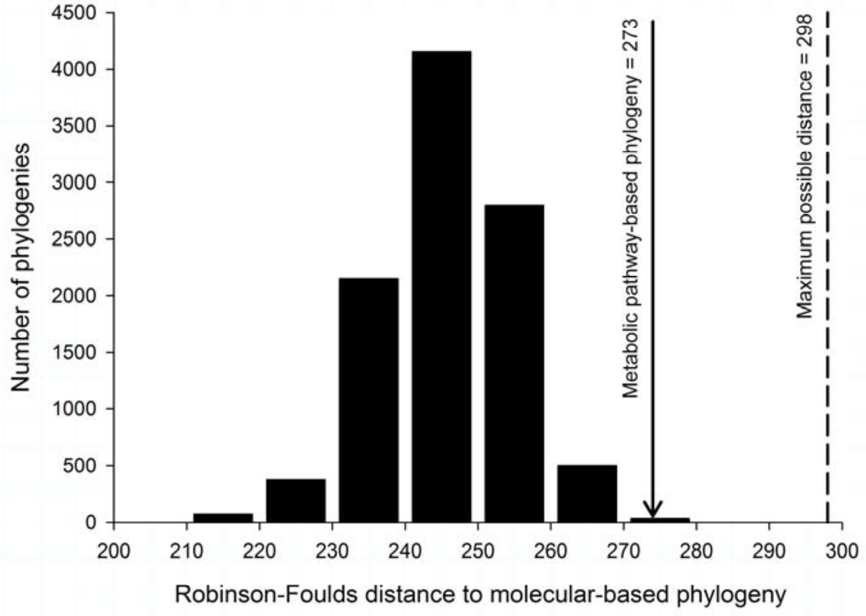

Figure S3. Distribution of Robinson-Foulds (RF) distances between 10,000 random trees and the molecular-based supertree. Arrows show the RF distance (=273) between the metabolic-pathway based phylogeny and the molecular-based supertree and the maximum possible distance between two trees with n = 152 species (=298) RF distances between the molecular-based supertree and each of the 10,000 simulated trees ranged from 206 to 277 (mean = 245.79); only 0.12% of the trees had distances ≥ 270.
