# Supplementary material for: Tradeoff between robustness and elaboration in carotenoid networks produces cycles of avian color diversification
Source: Biol Direct. 2015 Aug 20;10:45. doi: 10.1186/s13062-015-0073-6 (PMC4545997; doi:10.1186/s13062-015-0073-6)
Supplement: Additional file 6: Figure S4. — Diversity of characteristics of metabolic networks producing plumage carotenoid across bird species under this study. (PDF 110 kb) [file 13062_2015_73_MOESM6_ESM.pdf]

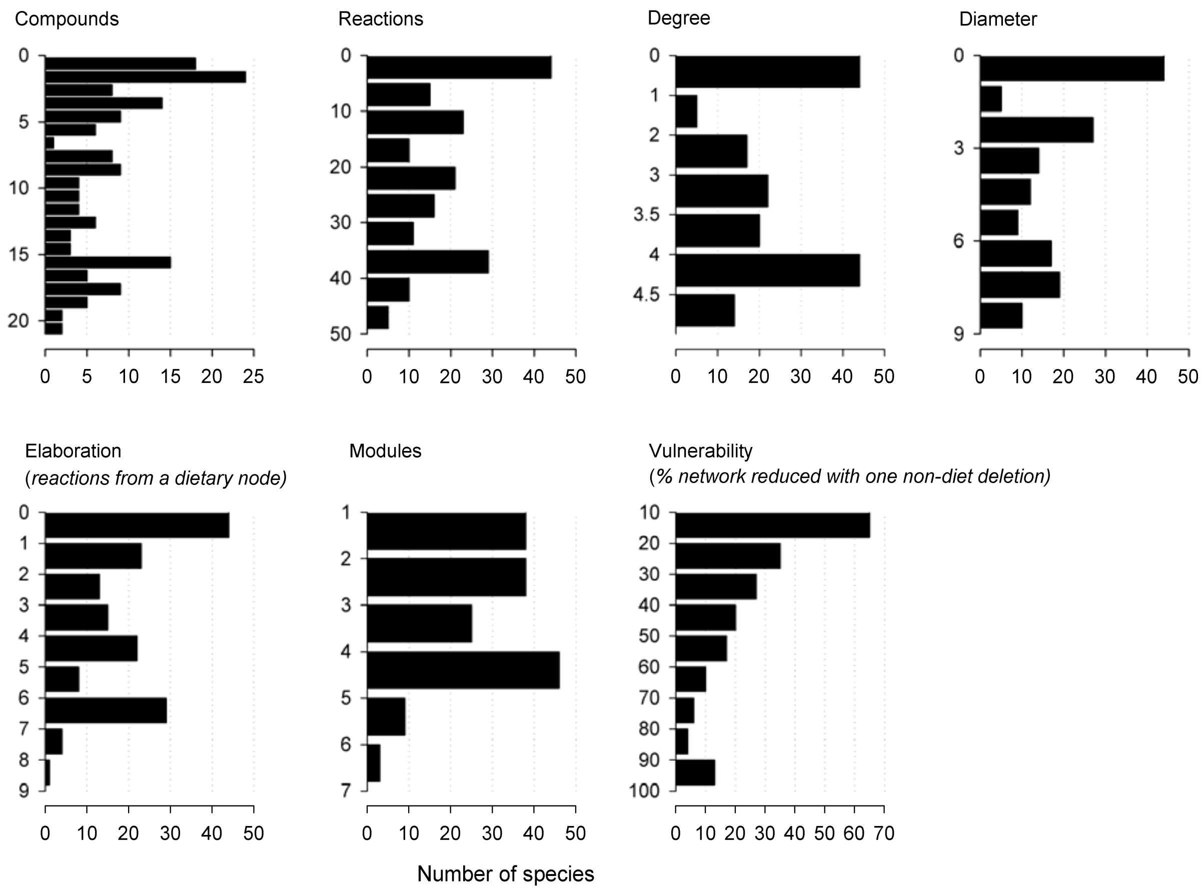

Figure S4. Diversity of characteristics of metabolic networks producing plumage carotenoid across bird species under this study. Additional File: Table S1 lists definitions and calculation details.
