# Supplementary material for: Tradeoff between robustness and elaboration in carotenoid networks produces cycles of avian color diversification
Source: Biol Direct. 2015 Aug 20;10:45. doi: 10.1186/s13062-015-0073-6 (PMC4545997; doi:10.1186/s13062-015-0073-6)
Supplement: Additional file 7: Table S2. — Estimated gains and losses of carotenoid compound in relation to their structural position in the network. (PDF 156 kb) [file 13062_2015_73_MOESM7_ESM.pdf]

Additional File: Table S2. Estimated gains and losses of carotenoid compound in relation to their position within the network. Only values from the models with the lower AIC values ("accepted rates") are shown.

Node/reaction (Fig. S2)	Model	k	s	r	log *L*	AIC
1-5	binary 2	2	0.001256	0.052124	-14.694843	33.389686
1-8	binary 2	2	0.007916	0.061918	-68.600022	141.200045
1-9	binary 2	2	0.007916	0.061918	-68.600022	141.200045
9-8	binary 2	2	0.009364	0.061599	-70.567786	145.135573
8-9	binary 2	2	0.009364	0.061599	-70.567786	145.135573
1-16	binary 2	2	0.009364	0.061599	-70.567786	145.135573
16-1	binary 2	2	0.017694	0.057185	-78.461588	160.923176
16-9	binary 2	2	0.009364	0.061599	-70.567786	145.135573
1-6	binary 2	2	0.000542	0.037642	-16.758114	37.516228
6-7	binary 2	2	0.000542	0.037642	-16.758114	37.516228
1-52	binary 1	1	0.000609	0.000609	-11.180567	24.361134
1-46	binary 2	2	0.002054	0.299154	-5.997449	15.994898
1-51	binary 2	2	0.014654	0.038923	-86.349569	176.699137
51-13	binary 2	2	0.006116	0.237839	-18.345958	40.691917
13-51	binary 2	2	0.006116	0.237839	-18.345958	40.691917
51-12	binary 2	2	0.014654	0.038923	-86.349569	176.699137
13-14	binary 2	2	0.006116	0.237839	-18.345958	40.691917
14-15	binary 2	2	0.006116	0.237839	-18.345958	40.691917
12-15	binary 2	2	0.006116	0.237839	-18.345958	40.691917
12-32	binary 2	2	0.01659	0.042966	-85.43238	174.864761
8-10	binary 1	1	0	0	-0.693147	3.386294
10-11	binary 1	1	0.000301	0.000301	-5.614873	13.229747
42-46	binary 1	1	0	0	-0.693147	3.386294
42-43	binary 1	1	0.000301	0.000301	-6.40325	14.806501
43-44	binary 1	1	0.000301	0.000301	-6.40325	14.806501
2-20	binary 2	2	0.00148	0.101496	-14.115386	32.230772
20-19	binary 2	2	0.00148	0.101496	-14.115386	32.230772
2-16	binary 2	2	0.017694	0.057185	-78.461588	160.923176
9-17	binary 1	1	0	0	-0.693147	3.386294
17-18	binary 1	1	0	0	-0.693147	3.386294
17-71	binary 1	1	0	0	-0.693147	3.386294
71-18	binary 1	1	0	0	-0.693147	3.386294
2-31	binary 2	2	0.03545	0.055402	-99.712303	203.424606
31-32	binary 2	2	0.03545	0.055402	-99.712303	203.424606
32-31	binary 2	2	0.03545	0.055402	-99.712303	203.424606
32-25	binary 2	2	0.03545	0.055402	-99.712303	203.424606
25-32	binary 2	2	0.03545	0.055402	-99.712303	203.424606

32-34	binary 1	1	0.042816	0.042816	-102.526693	207.053386	
34-32	binary 1	1	0.042816	0.042816	-102.526693	207.053386	
34-25	binary 2	2	0.03545	0.055402	-99.712303	203.424606	
25-34	binary 2	2	0.03545	0.055402	-99.712303	203.424606	
2-32	binary 1	1	0.042906	0.042906	-102.528957	207.057913	
4-30	binary 2	2	0.001087	0.142672	-5.941979	15.883958	
3-41	binary 2	2	0.373213	0.771898	-95.757592	195.515184	
41-40	binary 1	1	0.000609	0.000609	-11.149068	24.298136	
40-41	binary 1	1	0.000609	0.000609	-11.149068	24.298136	
41-35	binary 2	2	0.373213	0.771898	-95.757592	195.515184	
35-41	binary 2	2	0.373213	0.771898	-95.757592	195.515184	
3-35	binary 2	2	0.391696	0.763102	-97.151098	198.302196	
37-40	binary 1	1	0.000927	0.000927	-14.11583	30.231661	
40-37	binary 1	1	0.000927	0.000927	-14.11583	30.231661	
39-40	binary 1	1	0.000927	0.000927	-14.11583	30.231661	
35-39	binary 2	2	0.382243	0.767171	-96.469241	196.938483	
39-35	binary 2	2	0.382243	0.767171	-96.469241	196.938483	
35-37	binary 2	2	0.419132	0.748141	-99.019841	202.039681	
37-35	binary 2	2	0.419132	0.748141	-99.019841	202.039681	
39-37	binary 2	2	0.382243	0.767171	-96.469241	196.938483	
37-39	binary 2	2	0.382243	0.767171	-96.469241	196.938483	
35-36	binary 2	2	0.347252	0.763468	-94.23918	192.47836	
4-36	binary 2	2	0.020037	0.043602	-93.736519	191.473037	
36-32	binary 2	2	0.028681	0.058294	-95.55437	195.10874	
36-38	binary 2	2	0.021445	0.038088	-98.133397	200.266794	
38-36	binary 2	2	0.021445	0.038088	-98.133397	200.266794	
37-38	binary 2	2	0.3738	0.750255	-96.465328	196.930656	
38-37	binary 2	2	0.3738	0.750255	-96.465328	196.930656	
38-34	binary 1	1	0.027004	0.027004	-99.706393	201.412786	
34-38	binary 1	1	0.027004	0.027004	-99.706393	201.412786	
47-48	binary 2	2	0.001044	0.072314	-27.783897	59.567794	
49-50	binary 2	2	0.003153	0.207478	-18.835849	41.671698	
1-1	binary 2	2	0.010112	0.023223	-74.09632	152.19264	
2-2	binary 1	1	0.005602	0.005602	-57.290498	116.580996	
3-3	binary 2	2	0.005423	0.068351	-31.108907	66.217813	
4-4	binary 2	2	0.009067	0.098219	-39.208708	82.417416	
18-18	binary 2	2	0.016667	0.482582	-21.935132	47.870263	
42-42	binary 1	1	0.000924	0.000924	-14.627803	31.255606	
34-34	binary 2	2	0.001444	0.015289	-17.997361	39.994722	
37-37	binary 1	1	0.000302	0.000302	-5.721967	13.443935	
38-38	binary 1	1	0	0	-0.693147	3.386294	
26-26	binary 2	2	0.000607	0.032941	-8.830885	21.66177	
lutein	binary 1	1	0.004532	0.004532	-47.055494	96.110987	

zeaxanthin	binary 2	2	0.070636	0.025585	-94.795191	193.590381	
B-carotene	binary 2	2	0.466445	0.722713	-101.56072	207.12144	
β-cryptoxanthin	binary 1	1	0.039539	0.039539	-102.74	207.479837	
anhydrolutein	binary 2	2	0.001256	0.052124	-14.694843	33.389686	
7,8-dihydro-lutein	binary 2	2	0.000542	0.037642	-16.758114	37.516228	
9-Z-7,8-dihydro-lutein	binary 2	2	0.000542	0.037642	-16.758114	37.516228	
canary xanthophyll A	binary 2	2	0.007916	0.061918	-68.600022	141.200045	
canary xanthophyll B	binary 2	2	0.007916	0.061918	-68.600022	141.200045	
tunaxanthin A	binary 1	1	0.000301	0.000301	-5.614873	13.229747	
tunaxanthin F	binary 1	1	0.000301	0.000301	-5.614873	13.229747	
α-doradexanthin	binary 2	2	0.014654	0.038923	-86.349569	176.699137	
(3S,4R,3'R,6'R) 4-hydroxylutein	binary 2	2	0.006116	0.237839	-18.345958	40.691917	
fritschiellaxanthin	binary 2	2	0.006116	0.237839	-18.345958	40.691917	
papilioerythrinone	binary 2	2	0.006116	0.237839	-18.345958	40.691917	
3'-dehydrolutein	binary 2	2	0.008429	0.037618	-83.873187	171.746373	
piprixanthin	binary 1	1	0	0	-0.693147	3.386294	
rhodoxanthin	binary 2	2	0.016667	0.482582	-21.935132	47.870263	
7,8,7',8'-tetrahydro-zeaxanthin	binary 2	2	0.00148	0.101496	-14.115386	32.230772	
7,8-dihydro-zeaxanthin	binary 2	2	0.00148	0.101496	-14.115386	32.230772	
idoxanthin	binary 2	2	0.033445	0.05119	-98.9591	201.9182	
fucoxanthin	binary 2	2	0.000607	0.032941	-8.830885	21.66177	
7,8 dihydro β-cryptoxanthin	binary 2	2	0.001087	0.142672	-5.941979	15.883958	
4-hydroxyzeaxanthin	binary 2	2	0.033445	0.05119	-98.9591	201.9182	
(3S, 3'R)-adonixanthin	binary 1	1	0.042816	0.042816	-102.526693	207.053386	
(3S, 3'S)-astaxanthin	binary 2	2	0.033683	0.018476	-99.441058	202.882115	
echinenone	binary 2	2	0.419132	0.748141	-99.019841	202.039681	
3'-hydroxy-echinenone	binary 1	1	0.022709	0.022709	-97.026602	196.053205	
canthaxanthin	binary 2	2	0.437806	0.738043	-100.121134	204.242268	
adonirubin	binary 1	1	0.027293	0.027293	-101.580647	205.161294	
4-hydroxy-echinenone	binary 2	2	0.382243	0.767171	-96.469241	196.938483	
isozeaxanthin	binary 1	1	0.000927	0.000927	-14.11583	30.231661	
β-isocryptoxanthin	binary 2	2	0.373213	0.771898	-95.757592	195.515184	
α-carotene	binary 1	1	0.001244	0.001244	-18.058104	38.116208	
α-isocryptoxanthin	binary 1	1	0.000301	0.000301	-6.40325	14.806501	
phoenicopterone	binary 1	1	0.000301	0.000301	-6.40325	14.806501	
α-cryptoxanthin	binary 2	2	0.002054	0.299154	-5.997449	15.994898	
rubixanthin	binary 2	2	0.001044	0.072314	-27.783897	59.567794	
4-oxo-rubixanthin	binary 2	2	0.001044	0.072314	-27.783897	59.567794	
gazaniaxanthin	binary 2	2	0.003153	0.207478	-18.835849	41.671698	
4-oxo-gazaniaxanthin	binary 2	2	0.003153	0.207478	-18.835849	41.671698	

(3S,4R,3'S,6'R) 4-hydroxylutein	binary 2	2	0.014654	0.038923	-86.349569	176.699137	
cis-lutein	binary 1	1	0.000609	0.000609	-11.180567	24.361134	
resonance structure	binary 1	1	0	0	-0.693147	3.386294	
**model:**	binary-1 (gain/loss rates are equal) or binary-2 (gain/loss rates differ)						
**k**	number of parameters in the model						
**s**	rate of gain (in MYA)						
**r**	rate of loss (in MYA)						
**log L**	likelihood of the model using joint (conditional) ML reconstruction						
**AIC**	Akaike Information Criterion: AIC=2k-2logL						
