# Supplementary material for: Tradeoff between robustness and elaboration in carotenoid networks produces cycles of avian color diversification
Source: Biol Direct. 2015 Aug 20;10:45. doi: 10.1186/s13062-015-0073-6 (PMC4545997; doi:10.1186/s13062-015-0073-6)
Supplement: Additional file 8: Table S1. — Measures of carotenoid metabolic network used in the study. (PDF 157 kb) [file 13062_2015_73_MOESM8_ESM.pdf]

Additional File: Table S1. Measures of carotenoid metabolic network used in the study

Network Measure	Definition
**# Dietary compounds**	Number of carotenoid compounds that are dietary for a particular species
**Diameter**	The longest shortest distance (in reactions) between any two compounds in a species' network.
**Avg. shortest pathway**	The average length (in reactions) of the shortest paths between all of the pairs of compounds in a species' network.
**Avg. degree**	The average total degree (total number of incoming and outgoing reactions) of all of the compounds in the species' network.
**Dietary diameter**	The length (in reactions) of the longest shortest pathway between a dietary compound and an expressed compound in the species' network.
**Clustering**	Average clustering coefficient (C_i) of all of the compounds in a species' network. Measure of interconnectivity in the neighborhood of a compound. For each compound (i): $C_i = 2n/(k_i(k_i-1))$ Where n is the number of direct reactions connecting the k_i nearest neighbors (1 reaction away) from compound i. When C_i =1, a compound is at the center of a fully interlinked cluster. When C_i =0, a compound is part of a loosely connected group (Watts and Strogatz 1998).
**Modularity, Q**	Fraction of reactions in the network that form a module minus the expected value of in a network that has the same assignment of compounds into modules, but with random connections between compounds (Guimerà and Amaral 2005; Kashtan and Alon 2005; Olesen et al. 2007; Tamames et al. 2007).
**Edge sensitivity, ξ**	Measure of robustness. Fraction by which a network's scope is reduced on average when one reaction is deleted (Ebenhöh et al. 2005). The scopes of species networks are defined as all compounds that can be synthesized from the dietary compounds in the species networks, so the dietary compounds are not included in these calculations, but only the compounds that can be synthesized from them: $\xi=(1/\omega\sigma)\sum r\rho(r)$, where ω=number of reactions in the network, σ=original scope (total number of non-dietary compounds); $\rho(r)$=number of reactions whose deletion results in a reduction of scope r The sensitivity ranges $0\leq\xi\leq1$, when $\xi=0$ the network is completely robust (i.e., completely insensitive, such that any one reaction deletions do not affect the network's

	scope). When ξ=1 the network is not robust (i.e., maximally sensitive, such that removal of any reaction will cause the scope to disappear).
**Node sensitivity**	The average of the proportion of compounds lost in a species' network when a compound is removed from the species' network. The proportion of lost compounds includes the removed focal compound.
**Network OD**	Organismal degree (OD) is the number of species that have the compound in their species-specific network (Bernhardsson et al. 2011)
**In degree**	The number of incoming reactions for a node
**Out degree**	The number of outgoing reactions for a node
**Degree**	The sum of the in-degree and out-degree of a compound (the total number of incoming and outgoing reactions a compound has).
**Betweeness centrality**	Frequency with which a compound falls in the shortest path between any pairs of compounds within a network (Cohn and Marriott 1958; Freeman 1977; Brandes 2001).
**Closeness centrality (efficiency)**	Reciprocal of the average shortest path length. It is a measure of how fast information spreads from a given compound to other reachable compounds in the network. The closeness centrality of an isolated compound is 0 (Latora and Marchiori 2001; Newman 2003; Latora and Massimo 2004; Costa et al. 2007).
**Node clustering**	Clustering coefficient of a node. This is a measure of the degree of interconnectivity in the neighborhood of a compound. For each compound (*i*): $C_i = 2n/(k_i(k_i-1))$ Where n is the number of direct reactions connecting k_i nearest neighbors (1 reaction away) from compound *i*. When $C_i = 1$, a compound is at the center of a fully inter-linked cluster. When $C_i = 0$, a compound is part of a loosely connected group (Watts and Strogatz 1998).
**Vulnerability (V)**	$V_i = (CC-CCi)/CC$ where CC=global closeness centrality and CC_i = global closeness centrality when node *i* is removed. Where CC= (average of all of the nodes' closeness centrality)/(n-1) where n = number of nodes in network (Gol'dshtein et al. 2004; Latora and Massimo 2004).

References:

Bernhardsson, S., P. Gerlee, and L. Lizana. 2011. Structural correlations in bacterial metabolic networks. BMC Evolutionary Biology 11:20.

Brandes, U. 2001. A faster algorithm for betweenness centrality. The Journal of Mathematical Sociology 25:163 - 177.

Cohn, B. S. and M. Marriott. 1958. Networks and centres of integration in Indian civilization. Journal of Social Research 1:1-9.

Costa, L. d. F., F. A. Rodrigues, G. Travieso, and P. R. Villas Boas. 2007. Characterization of complex networks: a survey of measurements. Advances in Physics 56:167-242.

Ebenhöh, O., T. Handorf, and R. Heinrich. 2005. A cross species comparison of metabolic network functions. Genome Informatics 16:203-213.

Freeman, L. C. 1977. A set of measures of centrality based on betweeness. Sociometry 40:35-41.

Gol'dshtein, V., G. A. Koganov, and G. I. Surdutovich. 2004. Vulnerability and hierarchy of complex networks. Condensed Matter:arXiv:cond-mat/0409298v0409291.

Guimerà, R. and L. A. N. Amaral. 2005. Functional cartography of complex metabolic networks. Nature 433:895-900.

Kashtan, N. and U. Alon. 2005. Spontaneous evolution of modularity and network motifs. Proceedings of the National Academy of Sciences of the United States of America 102:13773-13778.

Latora, V. and M. Marchiori. 2001. Efficient behavior of small-world networks. Physical Review Letters 87:198701.

Latora, V. and M. Massimo. 2004. Vulnerability and protection of critical infrastructures. Condensed Matter:arXiv:cond-mat/0407491.

Newman, M. 2003. The structure and function of complex networks. SIAM Review 45:167-256.

Olesen, J. M., J. Bascompte, Y. L. Dupont, and P. Jordano. 2007. The modularity of pollination networks. Proceedings of the National Academy of Sciences of the United States of America 104:19891-19896.

Tamames, J., A. Moya, and A. Valencia. 2007. Modular organization in the reductive evolution of protein-protein interaction networks. Genome Biology 2007:R94.

Watts, D. J. and S. H. Strogatz. 1998. Collective dynamics of 'small-world' networks. Nature 393:440-442.
