# Supplementary material for: Tradeoff between robustness and elaboration in carotenoid networks produces cycles of avian color diversification
Source: Biol Direct. 2015 Aug 20;10:45. doi: 10.1186/s13062-015-0073-6 (PMC4545997; doi:10.1186/s13062-015-0073-6)
Supplement: Additional file 9: Table S3. — GenBank (GI) and taxonomy (TI) sequence identification numbers for study species. (PDF 80 kb) [file 13062_2015_73_MOESM9_ESM.pdf]

Additional File: Table S3. GenBank (GI) and taxonomy (TI) sequence identification numbers for species used in the study

Species name	GI number	TI number
Aegithalos caudatus	gi47550598	ti73327
Agelaius phoeniceus	gi9972115	ti39638
Amandava amandava	gi186703238	ti247729
Amandava subflava	gi186703180	ti187413
Anas platyrhynchos	gi3088713	ti8839
Anser anser	gi40795943	ti8843
Bombycilla cedrorum	gi15428351	ti161648
Bombycilla garrulus	gi15428359	ti125297
Bombycilla japonica	gi15428361	ti127872
Bucanetes githagineus	gi319890681	ti589648
Campephilus leucopogon	gi100965222	ti367949
Cardinalis cardinalis	gi17222094	ti98964
Carduelis atrata	gi46562098	ti160755
Carduelis cannabina	gi46562094	ti160757
Carduelis carduelis	gi17222088	ti37600
Carduelis chloris	gi17222091	ti37601
Carduelis cucullata	gi50295098	ti273489
Carduelis flammea	gi76364047	ti54769
Carduelis hornemanni	gi9230598	ti90719
Carduelis sinica	gi111034884	ti36253
Carduelis spinoides	gi33337421	ti54770
Carduelis spinus	gi111034880	ti160760
Carduelis tristis	gi197365483	ti54773
Carpodacus mexicanus	gi21616567	ti30427
Carpodacus nipalensis	gi18483314	ti175932
Carpodacus pulcherrimus	gi18378167	ti179409
Carpodacus roseus	gi111034874	ti175933
Carpodacus rubicilloides	gi18483318	ti175934
Carpodacus thura	gi18483320	ti64796
Carpodacus trifasciatus	gi18483322	ti175935
Chlorospingus pileatus	gi226511055	ti62171
Ciconia ciconia	gi3201653	ti8928
Coccothraustes coccothraustes	gi111034882	ti37609
Coereba flaveola	gi38606562	ti87177
Colaptes auratus	gi17834111	ti51355
Colaptes campestris	gi313191971	ti555237
Colaptes melanochloros	gi94421667	ti367944
Cyanistes caeruleus	gi342991	ti156563
Dendrocopos major	gi94421655	ti137523
Dendroica coronata	gi14581511	ti111975

Dendroica palmarum	gi206573691	ti231567
Dendroica petechia	gi206573695	ti123631
Dryocopus pileatus	gi2062438	ti51359
Emberiza citrinella	gi17222121	ti37595
Emberiza melanocephala	gi182893952	ti357696
Erithacus rubecula	gi40362743	ti37610
Erythrura prasina	gi186703178	ti194944
Erythrura trichroa	gi45685560	ti247731
Erythrura tricolor	gi186703268	ti522418
Eudocimus ruber	gi298371626	ti335483
Euplectes afer	gi45685568	ti229097
Euplectes ardens	gi165929803	ti247667
Euplectes axillaris	gi165929827	ti441676
Euplectes capensis	gi165929829	ti441675
Euplectes macrourus	gi165929853	ti187429
Euplectes orix	gi165929863	ti229098
Ficedula zanthopygia	gi126361499	ti369970
Foudia madagascariensis	gi165929871	ti441683
Fregata minor	gi306921149	ti57241
Fringilla coelebs	gi17222085	ti37598
Fringilla montifringilla	gi45685532	ti36255
Gallus gallus gallus	gi13516927	ti9031
Gallus lafayetii	gi13516935	ti9032
Gallus sonneratii	gi13516933	ti9033
Gallus varius	gi13516931	ti9034
Geothlypis trichas	gi22085456	ti135433
Haematospiza sipahi	gi18483328	ti175943
Icteria virens	gi22085506	ti135437
Icterus galbula	gi49615590	ti105513
Larus delawarensis	gi199601282	ti126683
Larus michahellis	gi34787063	ti119627
Leiothrix argentauris	gi183585463	ti201335
Leiothrix lutea	gi5712267	ti36275
Leucophaeus scoresbii	gi199601312	ti328019
Loxia curvirostra	gi45685524	ti64802
Loxia leucoptera bifasciata	gi18483334	ti128012
Loxia leucoptera leucoptera	gi9230586	ti128009
Luscinia calliope	gi306959216	ti229100
Malurus amabilis	gi34501174	ti165193
Malurus cyaneus	gi6469746	ti55807
Malurus lamberti	gi45385683	ti228352
Malurus leucopterus	gi169412226	ti228326
Malurus splendens	gi45385685	ti222595
Melanerpes aurifrons	gi94421647	ti371916
Melanerpes carolinus	gi4099763	ti56083

Melanerpes erythrocephalus	gi60281155	ti279962
Melanerpes formicivorus formicivorus	gi209916505	ti561549
Melanerpes lewis	gi209916585	ti372297
Melanerpes pucherani	gi209916587	ti372301
Melanerpes pygmaeus	gi209916599	ti372306
Melanerpes striatus	gi17834113	ti177297
Melanerpes uropygialis	gi209916589	ti177296
Meleagris gallopavo	gi343475	ti9103
Motacilla flava	gi24899290	ti180448
Mycerobas affinis	gi18483336	ti175945
Mycerobas carnipes	gi18483338	ti175949
Neochmia modesta	gi45685554	ti267974
Neochmia ruficauda clarescens	gi186703276	ti522433
Neochmia temporalis	gi186703262	ti247735
Neophron percnopterus	gi187475659	ti33608
Nesospiza acunhae acunhae	gi117647530	ti410743
Nesospiza wilkinsi wilkinsi	gi117647562	ti410746
Notiomystis cincta	gi357063853	ti366454
Oriolus oriolus	gi357063821	ti181099
Oriolus xanthornus	gi5712225	ti292210
Parus major	gi111034896	ti9157
Parus spilonotus	gi34596618	ti245580
Perdix perdix	gi212717305	ti9052
Pericrocotus divaricatus	gi357063801	ti424830
Periparus ater	gi323108157	ti156567
Phasianus colchicus	gi293632233	ti9054
Pheucticus ludovicianus	gi17222097	ti135444
Phoenicopterus andinus	gi482855	ti704177
Phoenicopterus ruber	gi33621777	ti9217
Picoides tridactylus	gi15216802	ti165751
Picoides villosus	gi2062454	ti51356
Picus awokera	gi190335690	ti187860
Picus canus	gi60281163	ti301969
Picus canus jessoensis	gi56785721	ti301971
Picus chlorolophus	gi190335580	ti367952
Picus erythropygius	gi190335534	ti535305
Picus flavinucha	gi190335564	ti367953
Picus mentalis	gi33520953	ti240710
Picus miniaceus	gi60281169	ti315366
Picus puniceus	gi190335532	ti535306
Picus rabieri	gi190335548	ti535304
Picus vaillantii	gi301154045	ti752243
Picus viridanus	gi190335552	ti535307
Picus viridis	gi190335566	ti100825
Picus vittatus	gi190335546	ti535309

Picus xanthopygaeus	gi190335538	ti535310
Pinicola enucleator	gi159136921	ti175947
Pipra aureola	gi156857562	ti467134
Pipra erythrocephala	gi156857564	ti456422
Pipra fasciicauda	gi25988543	ti114356
Pipra mentalis	gi83031302	ti360202
Pipra pipra	gi33867726	ti88179
Pipra rubrocapilla	gi156857556	ti467133
Piranga flava	gi156072848	ti63518
Piranga ludoviciana	gi24895117	ti63520
Piranga olivacea	gi156072842	ti62086
Piranga rubra	gi55794053	ti36733
Platalea ajaja	gi298371622	ti371920
Ploceus benghalensis	gi28191279	ti213159
Ploceus cucullatus	gi9972051	ti135448
Ploceus intermedius	gi45685570	ti267978
Ploceus luteolus	gi165929875	ti441695
Ploceus melanocephalus	gi165929877	ti441723
Ploceus taeniopterus	gi165929879	ti441712
Ploceus velatus	gi34501154	ti181103
Pyrrhoplectes epauletta	gi319890655	ti552619
Pyrrhula aurantiaca	gi319890441	ti928653
Pyrrhula erythaca	gi18483310	ti175815
Pyrrhula erythrocephala	gi319890565	ti552620
Pyrrhula pyrrhula	gi76364031	ti37607
Quelea cardinalis	gi165929881	ti158618
Quelea quelea	gi165929883	ti187445
Ramphastos toco	gi11036684	ti95723
Ramphocelus dimidiatus	gi156072776	ti460214
Regulus regulus	gi62942303	ti68468
Regulus satrapa	gi24817665	ti13245
Serinus canaria	gi66734390	ti9135
Serinus citrinella	gi46562122	ti163851
Serinus mozambicus	gi45685522	ti37603
Serinus pusillus	gi46562385	ti163852
Serinus serinus	gi17467189	ti37602
Setophaga ruticilla	gi22085466	ti182949
Sicalis flaveola	gi40362733	ti163868
Sphyrapicus varius	gi4099765	ti56079
Sterna elegans	gi55419851	ti297817
Taeniopygia guttata	gi40362727	ti59729
Tarsiger chrysaeus	gi306959355	ti358821
Telophorus bocagei	gi343477	ti9204
Tetrao urogallus	gi42557324	ti100830
Turdus merula	gi32825661	ti9187

Uragus sibiricus	gi111034890	ti179411
Vermivora ruficapilla	gi308224964	ti125952
Vermivora virginiae	gi308224966	ti190671
Zosterops japonicus	gi113171371	ti36299
