# Supplementary material for: Tradeoff between robustness and elaboration in carotenoid networks produces cycles of avian color diversification
Source: Biol Direct. 2015 Aug 20;10:45. doi: 10.1186/s13062-015-0073-6 (PMC4545997; doi:10.1186/s13062-015-0073-6)
Supplement: Additional file 10: Table S4. — References to phylogenies used to hierarchically build the topology of the supertree. (PDF 150 kb) [file 13062_2015_73_MOESM10_ESM.pdf]

Additional File: Table S4. References to phylogenies used to hierarchically build the topology of the supertree to which the cytB sequences were fitted. Dots indicate that tree hierarchy level was not needed to insert the species into the backbone tree.

Common name	Species name	Tree1	Tree2	Tree3	Tree4	Tree5
Long-tailed Tit	*Aegithalos caudatus*	(1)	(2)	(3)	.	.
Red-winged Blackbird	*Agelaius phoeniceus*	(4)	(2)	(3)	.	.
Red Munia	*Amandava amandava*	(5)	(6)	(2)	(3)	.
Zebra Waxbill	*Amandava subflava*	(5)	(6)	(2)	(3)	.
Mallard	*Anas platyrhynchos*	(3)	.	.	.	.
Greylag Goose	*Anser anser*	(3)	.	.	.	.
Cedar Waxwing	*Bombycilla cedrorum*	(7)	(2)	(3)	.	.
Bohemian Waxwing	*Bombycilla garrulus*	(7)	(2)	(3)	.	.
Japanese Waxwing	*Bombycilla japonica*	(7)	(2)	(3)	.	.
Trumpeter Finch	*Bucanetes githagineus*	(8)	(2)	(3)	.	.
Cream-backed Woodpecker	*Campephilus leucopogon*	(9)	(3)	.	.	.
Northern Cardinal	*Cardinalis cardinalis*	(4)	(2)	(3)	.	.
Black Siskin	*Carduelis atrata*	(8)	(2)	(3)	.	.
Linnet	*Carduelis cannabina*	(8)	(2)	(3)	.	.
European Goldfinch	*Carduelis carduelis*	(8)	(2)	(3)	.	.
European Greenfinch	*Carduelis chloris*	(8)	(2)	(3)	.	.
Red Siskin	*Carduelis cucullata*	(8)	(2)	(3)	.	.
Common Redpoll	*Carduelis flammea*	(8)	(2)	(3)	.	.
Hoary Redpoll	*Carduelis hornemanni*	(8)	(2)	(3)	.	.
Oriental Greenfinch	*Carduelis sinica*	(8)	(2)	(3)	.	.
Yellow-breasted Greenfinch	*Carduelis spinoides*	(8)	(2)	(3)	.	.
Eurasian Siskin	*Carduelis spinus*	(8)	(2)	(3)	.	.
American Goldfinch	*Carduelis tristis*	(8)	(2)	(3)	.	.
House Finch	*Carpodacus mexicanus*	(8)	(2)	(3)	.	.
Dark-breasted Rosefinch	*Carpodacus nipalensis*	(8)	(2)	(3)	.	.
Beautiful Rosefinch	*Carpodacus pulcherrimus*	(8)	(2)	(3)	.	.
Pallas' Rosefinch	*Carpodacus roseus*	(8)	(2)	(3)	.	.
Streaked Rosefinch	*Carpodacus rubicilloides*	(8)	(2)	(3)	.	.
White-browed Rosefinch	*Carpodacus thura*	(8)	(2)	(3)	.	.
Three-banded Rosefinch	*Carpodacus trifasciatus*	(10)	(8)	(2)	(3)	.
Sooty-capped Bush Tanager	*Chlorospingus pileatus*	(4)	(2)	(3)	.	.
White Stork	*Ciconia ciconia*	(3)	.	.	.	.
Hooded Grosbeak	*Coccothraustes abeillei*	(8)	(2)	(3)	.	.
Evening Grosbeak	*Coccothraustes vespertina*	(8)	(2)	(3)	.	.
Bananaquit	*Coereba flaveola*	(11)	(4)	(2)	(3)	.
Northern Flicker	*Colaptes auratus*	(12)	(9)	(3)	.	.
Campo Flicker	*Colaptes campestris*	(12)	(9)	(3)	.	.
Green-barred Woodpecker	*Colaptes melanochloros*	(12)	(9)	(3)	.	.
Blue Tit	*Cyanistes caeruleus*	(13)	(2)	(3)	.	.
Great Spotted Woodpecker	*Dendrocopos major*	(9)	(3)	.	.	.
Yellow-rumped Warbler	*Dendroica coronata*	(14)	(4)	(2)	(3)	.
Palm Warbler	*Dendroica palmarum*	(14)	(4)	(2)	(3)	.
Yellow Warbler	*Dendroica petechia*	(14)	(4)	(2)	(3)	.
Pileated Woodpecker	*Dryocopus pileatus*	(9)	(3)	.	.	.
Yellowhammer	*Emberiza citrinella*	(15)	(4)	(2)	(3)	.
Black-headed Bunting	*Emberiza melanocephala*	(15)	(4)	(2)	(3)	.

Robin	*Erithacus rubecula*	(16)	(2)	(3)	.	.
Gouldian Finch	*Erythrura gouldiae*	(5)	(6)	(2)	(3)	.
Red-headed Parrotfinch	*Erythrura psittacea*	.	.	.	.	.
Scarlet Ibis	*Eudocimus ruber*	(17)	(3)	.	.	.
Yellow-crowned Bishop	*Euplectes afer*	(18)	(2)	(3)	.	.
Red-collared Widowbird	*Euplectes ardens*	(16)	(2)	(3)	.	.
Red-shouldered Widowbird	*Euplectes axillaris*	(16)	(2)	(3)	.	.
Yellow Bishop	*Euplectes capensis*	(16)	(2)	(3)	.	.
Yellow-mantled Widowbird	*Euplectes macrourus*	(16)	(2)	(3)	.	.
Southern Red Bishop	*Euplectes orix*	(16)	(2)	(3)	.	.
Korean Flycatcher	*Ficedula zanthopygia*	(19)	(2)	(3)	.	.
Red Fody	*Foudia madagascariensis*	(16)	(2)	(3)	.	.
Great Frigatebird	*Fregata minor*	(3)	.	.	.	.
Chaffinch	*Fringilla coelebs*	(8)	(2)	(3)	.	.
Brambling	*Fringilla montifringilla*	(8)	(2)	(3)	.	.
Domestic Chicken	*Gallus gallus domesticus*	(20)	(3)	.	.	.
Common Yellowthroat	*Geothlypis trichas*	(14)	(4)	(2)	(3)	.
Scarlet Finch	*Haematospiza sipahi*	(8)	(2)	(3)	.	.
Yellow-breasted Chat	*Icteria virens*	(4)	(2)	(3)	.	.
Northern Oriole	*Icterus galbula*	(4)	(2)	(3)	.	.
Ring-billed Gull	*Larus delawarensis*	(21)	(3)	.	.	.
Yellow-legged Gull	*Larus michahellis*	(21)	(3)	.	.	.
Silver-eared Mesia	*Leiothrix argentauris*	(22)	(1)	(2)	(3)	.
Pekin Robin	*Leiothrix lutea*	(22)	(22)	(2)	(3)	.
Franklin's Gull	*Leucophaeus pipixcan*	(21)	(3)	.	.	.
Red Crossbill	*Loxia curvirostra*	(8)	(2)	(3)	.	.
White-winged Crossbill	*Loxia leucoptera*	(8)	(2)	(3)	.	.
Siberian Rubythroat	*Luscinia calliope*	(19)	(2)	(3)	.	.
Red-backed Fairywren	*Malurus melanocephalus*	(2)	(3)	.	.	.
White Woodpecker	*Melanerpes candidus*	(11)	(9)	(3)	.	.
Lewis' Woodpecker	*Melanerpes lewis*	(11)	(9)	(3)	.	.
Wild Turkey	*Meleagris gallopavo*	(23)	(20)	(3)	.	.
Yellow Wagtail	*Motacilla flava*	(2)	(3)	.	.	.
Collared Grosbeak	*Mycerobas affinis*	(10)	(8)	(2)	(3)	.
White-winged Grosbeak	*Mycerobas carnipes*	(10)	(8)	(2)	(3)	.
Black-and-yellow Grosbeak	*Mycerobas icteroides*	(24)	(10)	(8)	(2)	(3)
Spot-winged Grosbeak	*Mycerobas melanozanthos*	(24)	(10)	(8)	(2)	(3)
Star Finch	*Neochmia ruficauda*	(5)	(6)	(2)	(3)	.
Egyptian Vulture	*Neophron percnopterus*	(3)	.	.	.	.
Tristan Bunting	*Nesospiza acunhae*	(11)	(4)	(2)	(3)	.
Hihi	*Notiomystis cincta*	(25)	(2)	(3)	.	.
Golden Oriole	*Oriolus oriolus*	(26)	(2)	(3)	.	.
Black-hooded Oriole	*Oriolus xanthornus*	(26)	(2)	(3)	.	.
Great Tit	*Parus major*	(13)	(2)	(3)	.	.
Yellow-cheeked Tit	*Parus spilonotus*	(13)	(2)	(3)	.	.
Grey Partridge	*Perdix perdix*	(23)	(20)	(3)	.	.
Scarlet Minivet	*Pericrocotus flammeus*	(2)	(3)	.	.	.
Coal Tit	*Periparus ater*	(13)	(2)	(3)	.	.
Ring-necked Pheasant	*Phasianus colchicus*	(23)	(20)	(3)	.	.
Rose-breasted Grosbeak	*Pheucticus ludovicianus*	(4)	(2)	(3)	.	.
Andean Flamingo	*Phoenicopterus andinus*	(6)	(3)	.	.	.

American Flamingo	*Phoenicopterus ruber*	(6)	(3)	.	.	.
Lesser Flamingo	*Phoenicopterus minor*	(6)	(3)	.	.	.
James' Flamingo	*Phoenicopterus jamesi*	(6)	(3)	.	.	.
Chilean Flamingo	*Phoenicopterus chilensis*	(6)	(3)	.	.	.
Greater Flamingo	*Phoenicopterus roseus*	(6)	(3)	.	.	.
Three-toed Woodpecker	*Picoides tridactylus*	(9)	(3)	.	.	.
Hairy Woodpecker	*Picoides villosus*	(9)	(3)	.	.	.
Scaly-bellied Woodpecker	*Picus squamatus*	(27)	(9)	(3)	.	.
Green Woodpecker	*Picus viridis*	(27)	(9)	(3)	.	.
Pine Grosbeak	*Pinicola enucleator*	(8)	(2)	(3)	.	.
Round-tailed Manakin	*Pipra chloromeros*	(28)	(2)	(3)	.	.
Golden-headed Manakin	*Pipra erythrocephala*	(28)	(2)	(3)	.	.
Red-headed Manakin	*Pipra rubrocapilla*	(28)	(2)	(3)	.	.
Hepatic Tanager	*Piranga flava*	(4)	(2)	(3)	.	.
Western Tanager	*Piranga ludoviciana*	(4)	(2)	(3)	.	.
Scarlet Tanager	*Piranga olivacea*	(4)	(2)	(3)	.	.
Summer Tanager	*Piranga rubra*	(4)	(2)	(3)	.	.
Roseate Spoonbill	*Platalea ajaja*	(17)	(3)	.	.	.
Forest Weaver	*Ploceus bicolor*	.	.	.	.	.
Cape Weaver	*Ploceus capensis*	(11)	(16)	(2)	(3)	.
Village Weaver	*Ploceus cucullatus*	(11)	(16)	(2)	(3)	.
Nelicourvi Weaver	*Ploceus nelicourvi*	.	.	.	.	.
Baya Weaver	*Ploceus philippinus*	.	.	.	.	.
Sakalava Weaver	*Ploceus sakalava*	.	.	.	.	.
African Masked Weaver	*Ploceus velatus*	(11)	(16)	(2)	(3)	.
Gold-naped Finch	*Pyrrhoplectes epauletta*	(8)	(2)	(3)	.	.
Orange Bullfinch	*Pyrrhula aurantiaca*	(19)	(8)	(2)	(3)	.
Beavan's Bullfinch	*Pyrrhula erythaca*	(8)	(2)	(3)	.	.
Red-headed Bullfinch	*Pyrrhula erythrocephala*	(19)	(8)	(2)	(3)	.
Eurasian Bullfinch	*Pyrrhula pyrrhula*	(8)	(2)	(3)	.	.
Cardinal Quelea	*Quelea cardinalis*	(16)	(2)	(3)	.	.
Red-headed Quelea	*Quelea erythrops*	.	.	.	.	.
Red-billed Quelea	*Quelea quelea*	(16)	(2)	(3)	.	.
Toco Toucan	*Ramphastos toco*	(3)	.	.	.	.
Crimson-backed Tanager	*Ramphocelus dimidiatus*	(11)	(4)	(2)	(3)	.
Goldcrest	*Regulus regulus*	(29)	(2)	(3)	.	.
Golden-crowned Kinglet	*Regulus satrapa*	(29)	(2)	(3)	.	.
Common Canary	*Serinus canaria*	(8)	(2)	(3)	.	.
Citril Finch	*Serinus citrinella*	(8)	(2)	(3)	.	.
Yellow-fronted Canary	*Serinus mozambicus*	(8)	(2)	(3)	.	.
Red-fronted Serin	*Serinus pusillus*	(8)	(2)	(3)	.	.
European Serin	*Serinus serinus*	(8)	(2)	(3)	.	.
American Redstart	*Setophaga ruticilla*	(14)	(4)	(2)	(3)	.
Saffron Finch	*Sicalis flaveola*	(11)	(4)	(2)	(3)	.
Yellow-bellied Sapsucker	*Sphyrapicus varius*	(9)	(3)	.	.	.
Elegant Tern	*Sterna elegans*	(21)	(3)	.	.	.
Zebra Finch	*Taeniopygia guttata*	(5)	(6)	(2)	(3)	.
Golden Bush-robin	*Tarsiger chrysaeus*	(19)	(2)	(3)	.	.
Sulfur-breasted Bushrike	*Telophorus sulfureopectus*	(2)	(3)	.	.	.
Capercaillie	*Tetrao urogallus*	(20)	(3)	.	.	.
Eurasian Blackbird	*Turdus merula*	(19)	(2)	(3)	.	.

Long-tailed Rosefinch	*Uragus sibiricus*	(8)	(2)	(3)	.	.
Nashville Warbler	*Vermivora ruficapilla*	(14)	(4)	(2)	(3)	.
Virginia's Warbler	*Vermivora virginiae*	(14)	(4)	(2)	(3)	.
Japanese White-eye	*Zosterops japonicus*	(22)	(2)	(3)	.	.

Sources:

1: (Gelang et al. 2009); 2: (Barker et al. 2004); 3: (Hackett et al. 2008); 4: (Klicka et al. 2007); 5: (Arnaiz-Villena et al. 2009); 6: (Sibley and Ahlquist 1990); 7:(Spellman et al. 2008); 8: (Zuccon et al. 2012); 9: (Fuchs et al. 2007); 10: (Arnaiz-Villena et al. 2001); 11: (Sanderson et al. 2008); 12: (Moore et al. 2011); 13: (Gill et al. 2005); 14:(Lovette et al. 2010); 15: (Alström et al. 2008); 16: (Sangster et al. 2010); 17: (Chesser et al. 2010); 18: (Prager et al. 2008); 19: (Töpfer et al. 2011); 20: (Pereira and Baker 2006); 21:(Pons et al. 2005); 22: (Cibois 2003); 23: (Bonilla et al. 2010); 24: (Badyaev 1997); 25: (Driskell et al. 2007); 26: (Jønsson et al. 2010); 27: (Fuchs et al. 2008); 28: (Prum 1992); 29: (Päckert et al. 2009).

References

Alström, P., U. Olsson, F. Lei, H.-t. Wang, W. Gao, and P. Sundberg. 2008. Phylogeny and classification of the Old World Emberizini (Aves, Passeriformes). Molecular Phylogenetics and Evolution 47:960-973.

Arnaiz-Villena, A., J. Guillén, V. Ruiz-del-Valle, E. Lowy, J. Zamora, P. Varela, D. Stefani, and L. M. Allende. 2001. Phylogeography of crossbills, bullfinches, grosbeaks, and rosefinches. Cellular and Molecular Life Sciences 58:1159-1166.

Arnaiz-Villena, A., V. Ruiz-del-Valle, P. Gomez-Prieto, R. Reguera, C. Parga-Lozano, and I. Serrano-Vela. 2009. *Estrildinae* Finches (*Aves, Passeriformes*) from Africa, South Asia and Australia: a molecular phylogeographic study. The Open Ornithology Journal 2:29-36.

Badyaev, A. V. 1997. Altitudinal variation in sexual dimorphism: A new pattern and alternative hypotheses. Behavioral Ecology 8:675-690.

Barker, F. K., A. Cibois, P. Schikler, J. Feinstein, J. Cracraft, and D. B. Wake. 2004. Phylogeny and diversification of the largest avian radiation. Proceedings of the National Academy of Sciences of the United States of America 101:11040-11045.

Bonilla, A. J., E. L. Braun, and R. T. Kimball. 2010. Comparative molecular evolution and phylogenetic utility of 3'-UTRs and introns in Galliformes. Molecular Phylogenetics and Evolution 56:536-542.

Chesser, R. T., C. K. L. Yeung, C.-T. Yao, X.-H. Tian, and S.-H. Li. 2010. Molecular phylogeny of spoonbills (Aves: Threskiornithidae) based on mitochondrial DNA. Zootaxa 2603:53-60.

Cibois, A. 2003. Mitochondrial DNA phylogeny of babblers (Timalidae). The Auk 120:35-54.

Driskell, A., L. Christids, B. J. Gill, W. E. Boles, F. K. Barker, and N. W. Longmore. 2007. A new endemic family of New Zealand passerine birds: adding heat to a biodiversity hotspot. Australian Journal of Zoology 55:73-78.

Fuchs, J., J. I. Ohlson, P. G. P. Ericson, and E. Pasquet. 2007. Synchronous intercontinetal splits between assemblages of woodpeckers suggested by molecular data. Zool Scr 36:11-25.

Fuchs, J., J.-M. Pons, P. G. P. Ericson, C. Bonillo, A. Couloux, and E. Pasquet. 2008. Molecular support for rapid cladogenesis of the woodpecker clade Malarpicini, with further insights into the genus *Picus* (Piciformes: Picinae). Molecular Phylogenetics and Evolution 48:34-46.

Gelang, M., A. Cibois, E. Pasquet, U. Olsson, P. Alström, and P. G. P. Ericson. 2009. Phylogeny of babblers (Aves, Passeriformes): major lineages, family limits and classification. Zool Scr 38:225-236.

Gill, F. B., B. Slikas, and F. H. Sheldon. 2005. Phylogeny of titmice (Paridae): II. Species relationships based on sequences of the mitochondrial cytochrome-*b* gene. The Auk 122:121-143.

Hackett, S. J., R. T. Kimball, S. Reddy, R. C. K. Bowie, E. L. Braun, M. J. Braun, J. L. Chojnowski, W. A. Cox, K. Han, J. Harshman, C. J. Huddleston, B. D. Marks, K. J. Miglia, W. S. Moore, F. H. Sheldon, D. W. Steadman, C. C. Witt, and T. Yuri. 2008. A phylogenomic study of birds reveals their evolutionary history. Science 320:1763-1768.

Jønsson, K. A., R. C. K. Bowie, R. G. Moyle, M. Irestedt, L. Christidis, J. A. Norman, and J. Fjeldså. 2010. Phylogeny and biogeography of Oriolidae (Aves: Passeriformes). Ecography 33:232-241.

Klicka, J., K. Burns, and G. M. Spellman. 2007. Defining a monophyletic Cardinalini: a molecular perspective. Molecular Phylogenetics and Evolution 45:1014-1032.

Lovette, I. J., J. L. Pérez-Emán, J. P. Sullivan, R. C. Banks, I. Fiorentino, S. Córdoba-Córdoba, M. Echeverry-Galvis, F. K. Barker, K. J. Burns, J. Klicka, S. M. Lanyon, and E. Bermingham. 2010. A comprehensive multilocus phylogeny for the wood-warblers and a revised classification of the Parulidae (Aves). Molecular Phylogenetics and Evolution 57:753-770.

Moore, W. S., L. C. Overton, and K. J. Miglia. 2011. Mitochondrial DNA based phylogeny of the woodpecker genera *Colaptes* and *Piculus*, and implications for the history of woodpecker diversification in South America. Molecular Phylogenetics and Evolution 58:76-84.

Päckert, M., J. Martens, and L. L. Severinghaus. 2009. The Taiwan Firecrest (*Regulus goodfellowi*) belongs to the Goldcrest assemblage (*Regulus regulus* s. l.): evidence from mitochondrial DNA and the territorial song of the Regulidae. Journal of Ornithology 150:205-220.

Pereira, S. L. and A. J. Baker. 2006. A molecular timescale for galliform birds accounting for uncertainty in tim eestimates and heterogeneity of rates of DNA substitutions across lineages and sites. Molecular Phylogenetics and Evolution 38:499-509.

Pons, J. M., A. Hassanin, and P. A. Crochet. 2005. Phylogenetic relationships within the Laridae (Charadriiformes: Aves) inferred from mitochondrial markers. Molecular Phylogenetics and Evolution 37:686-699.

Prager, M., E. I. A. Johansson, and S. Andersson. 2008. A molecular phylogeny of the African widowbirds and bishops *Euplectes* spp. (Aves: Passeridae: Ploceinae). Molecular Phylogenetics and Evolution 46:290-302.

Prum, R. O. 1992. Syringeal morphology, phylogeny, and evolution of the Neotropical manakins (Aves: Pipridae). American Museum of Natural History Novitates 3043:1-65.

Sanderson, M. J., D. Boss, D. Chen, K. A. Cranston, and A. Wehe. 2008. The PhyLoTA browser: processing GenBank for molecular phylogenetics research. Systematic Biology 57:335-346.

Sangster, G., P. Alström, E. Forsmark, and U. Olsson. 2010. Multi-locus phylogenetic analysis of Old World chats and flycatchers reveals extensive paraphyly at family, subfamily and genus level (Aves: Muscicapidae). Molecular Phylogenetics and Evolution 57:380-392.

Sibley, C. G. and J. E. Ahlquist. 1990. Phylogeny and Classification of Birds. Yale University Press, New Haven.

Spellman, G. M., A. Cibois, R. G. Moyle, K. Winker, and F. K. Barker. 2008. Clarifying the systematics of an enigmatic avian lineage: What is a bombycillid? Molecular Phylogenetics and Evolution 49:1036-1040.

Töpfer, T., E. Haring, T. R. Birkhead, R. J. Lopes, L. L. Severinghaus, J. Martens, and M. Päckert. 2011. A molecular phylogeny of bullfinches *Pyrrhula* Brisson, 1760 (Aves: Fringillidae). Molecular Phylogenetics and Evolution 58:271-282.

Zuccon, D., R. Prŷs-Jones, P. C. Rasmussen, and P. G. P. Ericson. 2012. The phylogenetic relationships and generic limits of finches (Fringillidae). Molecular Phylogenetics and Evolution 62:581-596.
