# Supplementary material for: Tradeoff between robustness and elaboration in carotenoid networks produces cycles of avian color diversification
Source: Biol Direct. 2015 Aug 20;10:45. doi: 10.1186/s13062-015-0073-6 (PMC4545997; doi:10.1186/s13062-015-0073-6)
Supplement: Additional file 2: Figure S2. — Avian “subset” of global biochemical network of carotenoid metabolism. (PDF 225 kb) [file 13062_2015_73_MOESM2_ESM.pdf]

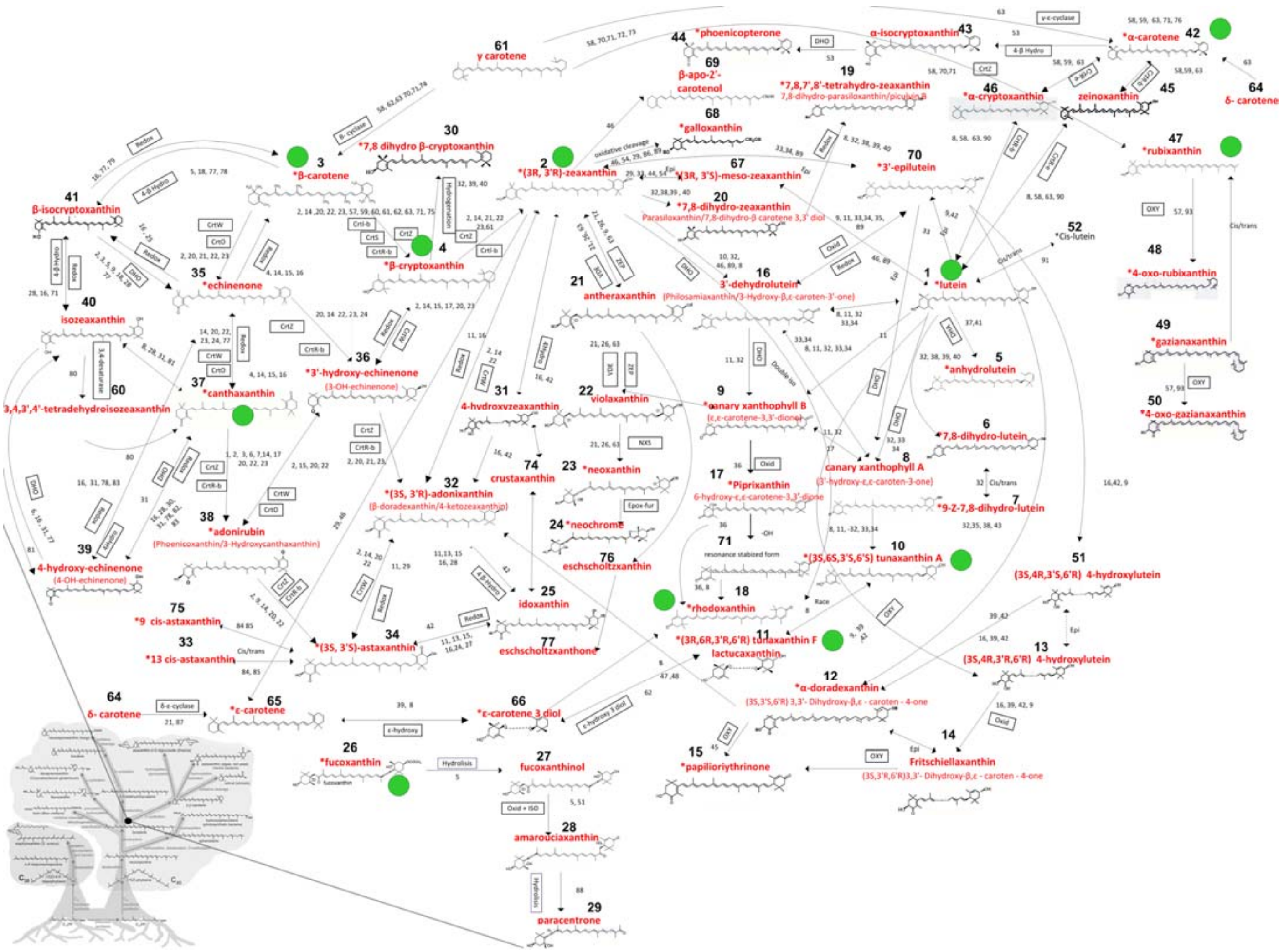

Figure S2. Avian “subset” of global biochemical network of carotenoid metabolism (Appendix S1). Green circles show dietary carotenoids, compound numbers and references (numbers over reactions) refer to those in Additional File: Appendix S1.
